# Perioperative faecal microbiome dynamics in patients undergoing rectal cancer surgery in the IMARI-trial

**DOI:** 10.1093/bjsopen/zrag046

**Published:** 2026-05-29

**Authors:** Kiedo Wienholts, Anne E Petersen, Claire P M van Helsdingen, Kevin Talboom, Mark Davids, Johannes H W de Wilt, Wouter J de Jonge, Pieter J Tanis, Roel Hompes, Joep P M Derikx, IMARI-study group, M D Slooter, K Talboom, C P M van Helsdingen, A E Petersen, S van Dieren, C Y Ponsioen, E C J Consten, P M Verheijen, D J Sikkenk, J P M Derikx, G D Musters, J D W van der Bilt, A W H van de Ven, J G Bloemen, J W A Burger, I Faneyte, T Verhagen, M F Lutke Holzik, I Masselink, L Morsink, M Gerhards, T M Karsten, S Festen, S van Dijk, W J de Jonge, W van der Meij, B J van Wely, S J Oosterling, J Scholten, L P S Stassen, J Melenhorst, C M J Gielen, K M M W Reynders, Y T van Loon, K P Wevers, E G G Verdaasdonk, W J A Brokelman, H L van Westreenen, E J A Steller, A D van Dalsen, J H W de Wilt, L Garms, E H J Belgers, E Ancion, G H E J Vijgen, J Heemskerk, J W A Leijtens, W Laméris, J B Tuynman

**Affiliations:** Department of Surgery, Amsterdam UMC, University of Amsterdam, Amsterdam, The Netherlands; Treatment and Quality of Life, Cancer Center Amsterdam, Amsterdam, The Netherlands; Imaging and Biomarkers, Cancer Center Amsterdam, Amsterdam, The Netherlands; Department of Paediatric Surgery, Emma Children’s Hospital, Amsterdam UMC, University of Amsterdam, Amsterdam, The Netherlands; Tytgat Institute for Liver and Intestinal Research, Amsterdam UMC, University of Amsterdam, Amsterdam, The Netherlands; Amsterdam Gastroenterology Endocrinology Metabolism Institute, Amsterdam UMC Locatie AMC, Amsterdam, The Netherlands; Amsterdam Reproduction and Development Institute, Amsterdam UMC Locatie AMC, Amsterdam, The Netherlands; Department of Paediatric Surgery, Emma Children’s Hospital, Amsterdam UMC, University of Amsterdam, Amsterdam, The Netherlands; Tytgat Institute for Liver and Intestinal Research, Amsterdam UMC, University of Amsterdam, Amsterdam, The Netherlands; Amsterdam Gastroenterology Endocrinology Metabolism Institute, Amsterdam UMC Locatie AMC, Amsterdam, The Netherlands; Amsterdam Reproduction and Development Institute, Amsterdam UMC Locatie AMC, Amsterdam, The Netherlands; Department of Surgery, Amsterdam UMC, University of Amsterdam, Amsterdam, The Netherlands; Treatment and Quality of Life, Cancer Center Amsterdam, Amsterdam, The Netherlands; Imaging and Biomarkers, Cancer Center Amsterdam, Amsterdam, The Netherlands; Department of Experimental Vascular Medicine, Amsterdam UMC, University of Amsterdam, Amsterdam, The Netherlands; Department of Surgery, Radboud University Medical Centre, Radboud Institute for Health Sciences, Nijmegen, The Netherlands; Tytgat Institute for Liver and Intestinal Research, Amsterdam UMC, University of Amsterdam, Amsterdam, The Netherlands; Amsterdam Gastroenterology Endocrinology Metabolism Institute, Amsterdam UMC Locatie AMC, Amsterdam, The Netherlands; Department of Surgery, University Hospital Bonn, Bonn, Germany; Department of Surgery, Amsterdam UMC, University of Amsterdam, Amsterdam, The Netherlands; Treatment and Quality of Life, Cancer Center Amsterdam, Amsterdam, The Netherlands; Imaging and Biomarkers, Cancer Center Amsterdam, Amsterdam, The Netherlands; Department of Surgical Oncology and Gastrointestinal Surgery, Erasmus MC, Rotterdam, The Netherlands; Department of Surgery, Amsterdam UMC, University of Amsterdam, Amsterdam, The Netherlands; Treatment and Quality of Life, Cancer Center Amsterdam, Amsterdam, The Netherlands; Imaging and Biomarkers, Cancer Center Amsterdam, Amsterdam, The Netherlands; Department of Paediatric Surgery, Emma Children’s Hospital, Amsterdam UMC, University of Amsterdam, Amsterdam, The Netherlands; Amsterdam Gastroenterology Endocrinology Metabolism Institute, Amsterdam UMC Locatie AMC, Amsterdam, The Netherlands; Amsterdam Reproduction and Development Institute, Amsterdam UMC Locatie AMC, Amsterdam, The Netherlands

## Abstract

**Background:**

The gut microbiome influences health by regulating metabolism, modulating the immune system, and protecting against pathogens. Surgical interventions, antibiotics, and dietary changes can disrupt this balance. In rectal cancer surgery, the perioperative microbiome’s role in selective digestive decontamination and ileostomy formation remains underexplored. This manuscript aimed to investigate perioperative microbiome dynamics in patients undergoing rectal cancer surgery, focusing on the impact of selective digestive decontamination and ileostomy.

**Methods:**

This study was part of the IMARI-trial. Faecal samples were collected before surgery and faecal or ileostomy samples on postoperative day 4. Microbial composition and diversity were assessed using 16S ribosomal RNA sequencing; alpha and beta diversity analyses were stratified by time point, ileostomy status, and selective digestive decontamination administration.

**Results:**

Of the 246 enrolled in the IMARI-trial, 214 patients were analysed. Postoperative samples showed significantly lower alpha diversity than preoperative samples, indicating reduced microbial richness and evenness across groups. Beta diversity analyses revealed distinct clustering between pre- and postoperative samples, with surgery explaining the largest variance in microbial composition (R^2^ = 15.6%; *P* < 0.001). Ileostomy status (R^2^ = 9.6%; *P* < 0.001) and selective digestive decontamination administration (R^2^ = 3.1%; *P* = 0.002) also contributed significantly. Notable taxonomic shifts included increased postoperative abundances of *Enterococcus* and *Klebsiella*, alongside reduced Firmicutes genera, particularly in ileostomy samples.

**Conclusion:**

Surgery and perioperative factors induce significant, early microbiome alterations, favouring facultative anaerobes over obligate anaerobes. Although selective digestive decontamination selectively modulates specific taxa, its overall impact on diversity appears less pronounced than surgery and ileostomy status. These findings underscore the need for studies linking perioperative microbiome trajectories to clinical endpoints and for evaluating microbiome-informed perioperative strategies, including antibiotic stewardship, diet, or microbial therapeutics, in rectal cancer care.

## Teaser

In a multicentre prospective cohort, the authors longitudinally profiled the faecal microbiome before and on postoperative day 4 after rectal cancer surgery, stratified by selective digestive decontamination and ileostomy status. Surgery was the dominant driver of change, with uniform postoperative loss of alpha diversity and rises in facultative anaerobes (for example *Enterococcus*, *Klebsiella*); ileostomy and SDD contributed smaller, selective effects. These data define early perioperative disruption and provide a foundation for linking microbial trajectories to clinical outcomes and for evaluating microbiome-informed perioperative strategies.

## Introduction

The gut microbiome is increasingly recognized for its significant contributions to human health, including roles in metabolic regulation, immune system modulation, and defence against pathogenic organisms^1^. Its composition is highly sensitive to perioperative influences such as surgical stress, tissue damage, dietary modifications, and antibiotics^2^. These factors can disrupt microbial balance, potentially affecting recovery after surgery and increasing the risk of complications^3^.

Rectal cancer surgery, which involves resection of the rectum and often the creation of an anastomosis, presents unique challenges in this context. The procedure disrupts the full thickness of the intestinal wall, initiating a complex healing response in the presence of a dynamic and diverse gut microbiome. This microbiome plays a key role in tissue repair and immune modulation, potentially influencing the healing process and postoperative outcomes in rodent studies^4^. For example, butyrate-producing bacteria are associated with improved anastomotic healing^5^, whereas collagenase-producing *Enterococcus faecalis* has been shown to induce anastomotic leakage (AL) in preclinical models^6^.

Despite growing interest in the microbiome's role in surgical outcomes, knowledge gaps persist, particularly regarding its dynamic response to rectal cancer resection. Despite an increasing use of selective digestive decontamination (SDD) to mitigate the microbiota's potential adverse effects on healing, the consequences on microbiome composition and dynamics remain poorly understood. Furthermore, it is largely unexplored to what extent faecal samples from a diverting ileostomy differ from faecal samples taken from a patient with a non-diverted anastomosis. Much of the current evidence on microbiome composition in the perioperative setting for rectal cancer comes from animal models, with limited longitudinal human data primarily from a pilot study. Moreover, most clinical studies^7,8^ evaluate the microbiome in the context of surgery at a single time point, thereby failing to capture its dynamic, rapidly evolving nature during the perioperative period.

Therefore, the primary objective of this study, conducted as part of the ongoing prospective clinical IMARI-trial^9^, was to investigate the dynamics of the perioperative faecal human microbiome in patients undergoing rectal cancer surgery, with specific focus on the impact of SDD and stoma formation. This will increase knowledge of key determinants that may influence ultimate analyses of the microbiome's role in postoperative complications, such as AL.

## Methods

### Population

This study utilized samples collected from patients enrolled in the IMARI-trial^9^. This is a multicentre prospective clinical effectiveness trial designed to evaluate the efficacy of a multi-interventional programme in reducing AL and improving its management. The trial began recruiting participants in January 2020 and the primary endpoint of this study is the 1-year anastomotic integrity rate. Inclusion criteria are (1) planned to undergo low anterior resection (LAR) for primary rectal cancer as defined by the international consensus definition for rectal cancer^10^, regrowth of rectal cancer in a watch and wait protocol, or completion and/or salvage surgery after local excision for rectal cancer; (2) willing to complete quality-of-life questionnaires and comply with schedule of outpatient follow-up visits; and (3) ≥ 18 years old. Exclusion criteria are (1) LAR without colorectal or coloanal anastomosis; (2) locally advanced rectal cancer, expected to require beyond-total mesorectal excision or multivisceral resection; and (3) synchronous colonic resections. The patients included in this study were from the control cohort, in which the multi-interventional programme was not yet implemented, thereby reflecting routine perioperative care at the participating hospitals. As this microbiome substudy focused on perioperative microbial patterns within the control cohort, it was neither powered nor prespecified to assess associations with clinical outcomes such as AL or infectious complications. SDD was not uniformly applied across participating centres but was administered in centres where it was part of standard perioperative care. In these centres, SDD was given according to the IMARI protocol or a comparable local protocol. SDD consisted of an oral suspension administered for 3 consecutive days before surgery at a dose of 10 ml four times daily. Each 10 ml dose contained colistin (100 mg), tobramycin (80 mg), and either amphotericin B (500 mg) or nystatin (2 000 000 IU).

### Sample collection

Samples analysed in this study were obtained from patients who provided additional consent for storage in the IMARI biobank. The samples were collected at two predefined time points: (1) stool sample before surgery before initiation of mechanical bowel preparation, SDD, or prophylactic intravenous antibiotics; and (2) stool sample at postoperative day 4 or an ileostomy sample at postoperative day 4 if the patient had received an ileostomy during surgery. These samples were stored at −80°C without buffer in a sterile Eppendorf tube as soon as possible, after a maximum of 24 hours in a refrigerator. Bacterial DNA was extracted from these samples and subsequently analysed by sequencing the amplified 16S ribosomal (r)RNA genes.

### DNA isolation, 16S gene amplification, purification, and MiSeq sequencing

Total genomic DNA was isolated using an adapted repeated bead-beating method^11^. 16S genes were amplified with Index primers^12^ with an adapted polymerase chain reaction (PCR) method. After confirmation of PCR products by agarose gel electrophoresis, the samples were purified and equal-molar pooled before being sequenced on an Illumina MiSeq^®^ (V3,600) sequencing (Illumina, Inc., San Diego, CA, USA)^12,13^.

### Bioinformatic analysis

Amplicon sequences were parsed using a vsearch^14^ (v2.15.2)-based pipeline. Paired-end reads were merged, with a maximum difference of 100 and allowing for staggered overlap. Amplicon sequence variants (ASVs) were inferred from reads with an expected error rate below 1.5 using the cluster_unoise with centroids algorithm and a minsize of 4, after which chimeras were removed using the uchime3 denovo method. For each sample, ASV abundances were determined by mapping the merged reads against ASV sequences using usearch_global with a 0.97 distance cutoff. Taxonomy was assigned using R (4.2.0) and the dada2^15^ assign taxonomy function using the SILVA (v132)^16^ reference database. A phylogenetic tree was generated using mafft (v7.310)^17^ and Fasttree (2.1.11)^18^.

Before statistical analysis, count tables were subsampled to 10 000 counts to correct for sampling bias. To assess compositional differences, the authors employed a customized permutational multivariate analysis of variance (PERMANOVA) analysis using the vegan adonis2 function, which supports nested permutations with missing data. Instead of randomly shuffling data with missing values onto the existing labels in the data set, a complete design-permutation framework with subject-specific strata was implemented. Labels corresponding to missing data were excluded, and the F-statistic for the permuted model was calculated. This process was repeated 10 000 times and compared with the unpermuted model. Effects of the longitudinal time point, SDD administration, and ileostomy status on individual genera were tested using a linear mixed model with random intercepts for the individual patients. Effect sizes were scaled by the mean relative abundance, and *P* values were adjusted for multiple comparisons using the Benjamini–Hochberg procedure.

### Outcomes of interest

The primary outcomes of this microbiome substudy were perioperative changes in microbial diversity and composition between the preoperative sample and the postoperative day 4 sample. Alpha diversity was assessed using the Shannon index and Faith’s Phylogenetic Diversity, and was compared according to time point, SDD administration, and ileostomy status. Beta diversity was assessed using weighted UniFrac distances and visualized with principal coordinates analysis (PCoA); differences in overall microbial community composition were formally tested using PERMANOVA.

Secondary outcomes were differences in the relative abundance of individual bacterial genera according to time point, SDD administration, and ileostomy status. These were assessed using linear mixed models with random intercepts for individual patients, with effect sizes scaled by mean relative abundance and *P* values adjusted for multiple testing using the Benjamini–Hochberg procedure. In addition, descriptive analyses of the most prevalent genera were performed to characterize perioperative microbiome profiles across subgroups.

### Statistics

Baseline characteristics of patients were reported using descriptive statistics. Categorical data were compared using the χ^2^ test or Fisher’s exact test when appropriate, and were presented as total counts with percentages, unless otherwise indicated. Numerical data were compared using the Mann–Whitney *U* test due to non-normal distributions of all numerical variables and were reported as medians with interquartile ranges (i.q.r), unless otherwise indicated. The statistical significance level was set at *P* < 0.050. IBM SPSS Statistics for Windows (v.28.0.1.1, IBM Corp., Armonk, New York, USA) was used for the statistical analyses.

This study was approved by the Medical Ethical Committee and Biobank committee of the Amsterdam UMC, University of Amsterdam. All participants provided informed consent for study participation and a separate consent for sample storage in the IMARI biobank.

The trial has been registered with the Dutch Central Committee on Research Involving Human Subjects (registration number: NL67600.018.18); and *Onderzoek-met-Mensen* database (registration numbers: NL-OMON26456; NL-OMON55903 (www.onderzoekmetmensen.nl/en)). Furthermore, the protocol has been published in *BMC Surgery*^9^. The preregistered protocol did not include a predefined analysis plan for the microbiome research.

## Results

### Study population

**Fig. 1:**
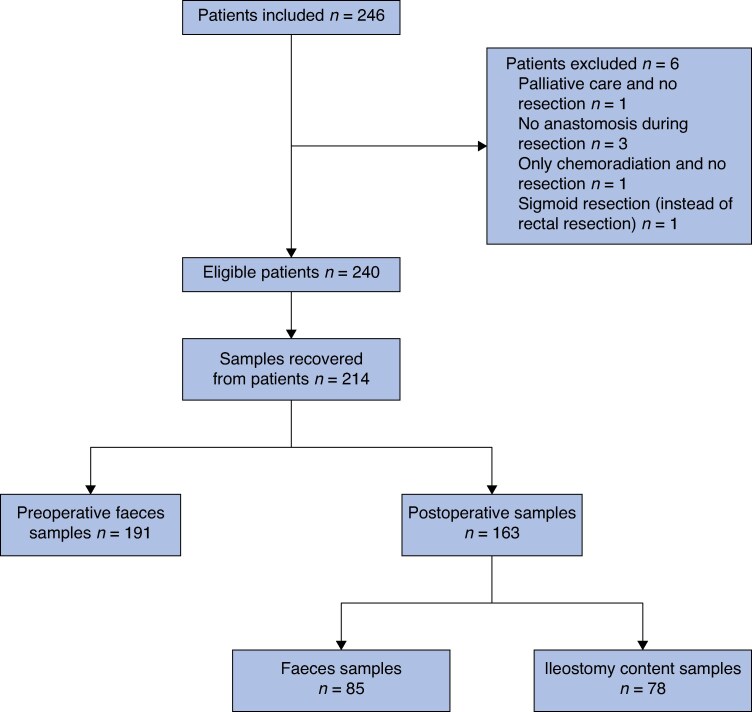
Flowchart depicting the inclusion process for the control cohort of the IMARI-trial The chart shows patient screening, exclusions, and the final numbers of patients included in the study, stratified by the availability of preoperative and postoperative samples.

The inclusion process for the clinical trial is outlined in the flowchart (*Fig. 1*). A total of 246 patients were initially included in the control cohort of the IMARI-trial. Of these, six patients were excluded due to various reasons. Among 240 eligible patients, samples were successfully recovered from 214 patients, including 191 preoperative faecal samples and 163 postoperative samples. The postoperative samples comprised 85 faecal samples and 78 ileostomy content samples. The baseline characteristics of the 214 included patients are summarized in *Table 1*. The majority (69.2%) of patients were male, and the median age at the time of index surgery was 63 years (i.q.r. 55–68). The median body mass index (BMI) was 25.2 kg/m^2^ (i.q.r. 23.0–27.7). Patients were stratified by SDD administration and ileostomy status, with comparisons provided for each subgroup.

**Table 1:** Baseline characteristics

	Total *n* = 214	SDD *n* = 85	No SDD *n* = 129	P*	Ileostomy *n* = 78	No ileostomy *n* = 136	P*
**Sex**							
Male	148 (69.2%)	56 (66%)	92 (71.3%)	0.400	61 (78%)	87 (64.0%)	0.030
Female	66 (30.8%)	29 (34%)	37 (28.7)	0.400	17 (22%)	49 (36.0%)	0.030
Age at time of surgery (years), median (i.q.r.)	63 (55–68)	63 (58–68)	61 (53–69)	0.168†	61 (51–67)	63 (57–70)	0.008†
BMI (kg/m^2^), median (i.q.r.)	25.2 (23.0–27.7)	25.3 (23.0–27.5)	25.2 (22.9–28.0)	0.836†	24.9 (23.3–27.8)	25.3 (22.5–27.7)	0.727†
Tumour from anorectal junction on MRI (mm), median (i.q.r.)	50 (24–66)	50 (26–70)	48 (20–63)	0.247†	35 (15–52)	55 (35–80)	<0.001†
**Clinical T category on MRI**							
T1	9 (4.2%)	3 (4%)	6 (4.7%)	0.693	0	9 (6.6%)	0.020
T2	53 (24.8%)	18 (21%)	35 (27.1%)	0.331	19 (24%)	34 (25.0%)	0.917
T3	132 (61.7%)	55 (65%)	77 (59.7%)	0.434	53 (68%)	79 (58.1%)	0.153
T4	10 (4.7%)	5 (6%)	5 (3.9%)	0.492	3 (4%)	7 (5.1%)	0.664
Tx	8 (3.7%)	3 (4%)	5 (3.9%)	0.896	3 (4%)	5 (3.7%)	0.950
Missing	2 (0.9%)	1 (1%)	1 (0.8%)	0.765	0	2 (1.5%)	0.282
**Clinical N category on MRI**							
N0	109 (50.9%)	45 (53%)	64 (49.6%)	0.737	36 (46%)	73 (53.7%)	0.289
N1	64 (29.9%)	29 (34%)	35 (27.1%)	0.316	29 (37%)	35 (25.7%)	0.078
N2	34 (15.9%)	9 (11%)	25 (19.4%)	0.075	12 (15%)	22 (16.2%)	0.879
Nx	2 (0.9%)	1 (1%)	1 (0.8%)	0.776	0	2 (1.5%)	0.282
Missing	5 (2.3%)	1 (1%)	4 (3.1%)	0.362	1 (1%)	4 (2.9%)	0.439
**ASA classification**							
ASA I	38 (17.8%)	16 (19%)	22 (17.1%)	0.740	15 (19%)	23 (16.9%)	0.669
ASA II	146 (68.2%)	57 (67%)	89 (69.0%)	0.766	51 (65%)	95 (69.9%)	0.499
ASA III	30 (14.0%)	12 (14%)	18 (14.0%)	0.973	12 (15%)	18 (13.2%)	0.663
**Comorbidities**‡							
No comorbidity	99 (46.3%)	41 (48%)	58 (45.0%)	0.638	36 (46%)	63 (46.3%)	0.981
Cardiac	29 (13.6%)	14 (16%)	15 (11.6%)	0.311	12 (15%)	17 (12.5%)	0.553
Peripheral vascular disease	40 (18.7%)	24 (28%)	16 (12.4%)	0.004	11 (14%)	29 (21.3%)	0.192
Pulmonary	29 (13.6%)	10 (12%)	19 (14.7%)	0.535	14 (18%)	15 (11.0%)	0.155
Diabetes	15 (7.0%)	9 (11%)	6 (4.7%)	0.096	8 (10%)	7 (5.1%)	0.159
Other	64 (29.9%)	21 (25%)	43 (33.3%)	0.177	23 (29%)	41 (30.1)	0.919
**Neoadjuvant therapy**	96 (44.9%)	37 (44%)	59 (45.7%)	0.751	43 (55%)	53 (39.0%)	0.022
Short-course radiotherapy (5 × 5 Gy)	43 (20.1%)	20 (54%)	23 (39.0%)	0.148	17 (40%)	26 (49.1%)	0.351
Short-course radiotherapy (5 × 5 Gy) followed by chemotherapy	7 (3.3%)	0	7 (11.9%)	0.030	4 (9%)	3 (5.7%)	0.495
Chemoradiotherapy	45 (21.0%)	16 (43%)	29 (49.2)	0.572	21 (49%)	24 (45.3%)	0.729
Chemoradiotherapy followed by chemotherapy	1 (0.5%)	1 (3%)	0	0.204	1 (2%)	0	0.264
Attempted endoluminal excision of tumour	35 (16.4%)	13 (15%)	22 (17.1%)	0.733	12 (15%)	23 (16.9%)	0.771
**Bowel preparation**‡							
Oral	198 (92.5%)	74 (87%)	124 (96.1%)	0.014	74 (95%)	124 (91.2%)	0.323
Rectal enema	30 (14.0%)	9 (11%)	21 (16.3%)	0.214	9 (12%)	21 (15.4%)	0.429
None	2 (0.9%)	2 (2%)	0	0.08	0	2 (1.5%)	0.282
**Prehabilitation programme**‡							
Smoking cessation	1 (0.5%)	1 (1%)	0	0.217	0	1 (0.7%)	0.448
Alcohol reduction	4 (1.9%)	3 (4%)	1 (0.8%)	0.145	0	4 (2.9%)	0.126
Exercise programme	15 (7.0%)	4 (5%)	11 (8.5%)	0.284	2 (3%)	13 (9.6%)	0.054
Diet programme	10 (4.7%)	5 (6%)	5 (3.9%)	0.496	0	10 (7.4%)	0.014
Other	3 (1.4%)	0	3 (2.3%)	0.157	0	3 (2.2%)	0.187
None	197 (92.1%)	80 (94%)	117 (90.7%)	0.365	76 (97%)	121 (89.0%)	0.028
Operative time (minutes), median (i.q.r.)	238 (186–283)	240 (182–320)	234 (189–272)	0.495†	268 (229–343)	204 (179–260)	<0.001†
**Surgical technique**							
Laparoscopy	144 (67.3%)	41 (48%)	103 (79.8%)	<0.001	59 (76%)	85 (62.5%)	0.049
Robot	68 (31.8%)	43 (51%)	25 (19.4%)	<0.001	18 (23%)	50 (36.8%)	0.038
Open	2 (0.9%)	1 (1%)	1 (0.8%)	0.765	1 (1%)	1 (0.7%)	0.689
+ Transanal	83 (38.8%)	26 (31%)	57 (44.2%)	0.037	47 (62%)	36 (26.5%)	<0.001
Preoperative intravenous antibiotics	207 (96.7%)	81 (95%)	126 (97.7%)	0.338	74 (95%)	133 (97.8%)	0.247
Repeated intraoperative intravenous antibiotics after 4 hours	71 (33.2%)	36 (42%)	35 (27.1%)	0.010	35 (47%)	36 (27.7%)	0.006
Continued intravenous antibiotics prophylaxis after surgery	6 (2.8%)	2 (2%)	4 (3.1%)	0.746	5 (6%)	1 (0.7%)	0.016

Values are *n* (%) unless otherwise stated. SDD, selective digestive decontamination; i.q.r., interquartile range; BMI, body mass index; MRI, magnetic resonance imaging; ASA, American Society of Anesthesiologists. *χ^2^ test or Fisher’s exact test for categorical variables with counts < 5, except †Mann–Whitney *U* test for numerical variables (due to non-normal distribution for all numerical variables); ‡multiple symptoms possible per patient.

Patients in the SDD group (85) were given oral bowel preparation significantly less often (87% *versus* 96.1%; *P* = 0.014). Patients with a diverting ileostomy (78) were younger (61 *versus* 63 years; *P* = 0.008) and more frequently male (78% *versus* 64.0%; *P* = 0.030) as compared with those without an ileostomy. Furthermore, patients with an ileostomy had more distal tumours (distance to the anorectal junction: 35 mm *versus* 55 mm; *P* < 0.001) and were more frequently treated with neoadjuvant therapy (55.1% *versus* 39.0%; *P* = 0.022).

Preoperative intravenous antibiotics were administered to nearly all patients (96.7%), with additional intraoperative antibiotics after 4 hours of surgery in 33.2% of cases. This was more common in the SDD group (42% *versus* 27.1%; *P* = 0.010) and in patients with ileostomies (47% *versus* 27.7%; *P* = 0.006). Postoperative continuation of intravenous antibiotics was also more frequent in the ileostomy group (6% *versus* 0.7%; *P* = 0.016).

### Impact of SDD administration and ileostomy status on alpha diversity of the microbiome

**Fig. 2:**
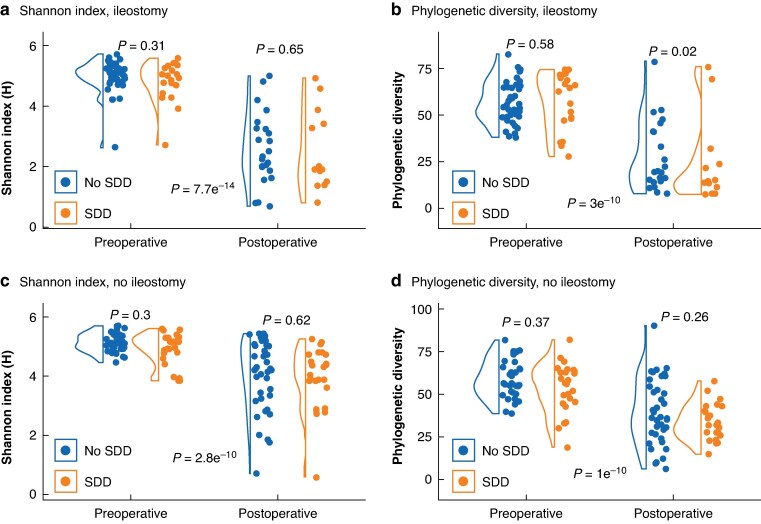
Alpha diversity analyses using Shannon index and Phylogenetic Diversity in preoperative and postoperative samples stratified by ileostomy status and SDD administration **a** Shannon index, ileostomy, **b** Phylogenetic Diversity, ileostomy, **c** Shannon index, no ileostomy, **d** Phylogenetic Diversity, no ileostomy. *P* values obtained using Wilcoxon rank-sum test for unpaired data (No SDD *versus* SDD) and Wilcoxon signed-rank test for paired data (preoperative *versus* postoperatieve). SDD, selective digestive decontamination.

Alpha diversity was analysed using the Shannon index and Phylogenetic Diversity, stratified by ileostomy status (faeces or ileostomy sample), SDD administration, and time points (preoperative and postoperative) (*Fig. 2*). Significant reductions in alpha diversity were observed after surgery in both the ileostomy and no ileostomy groups. For the Shannon index, the analysis revealed a significant reduction after surgery for patients who had an ileostomy (*P* = 7.700 × 10^−14^) and patients without an ileostomy (*P* = 2.800 × 10^−10^). Similarly, for Phylogenetic Diversity, postoperative reductions were significant in both patients who had an ileostomy (*P* = 3.000 × 10^−10^) and patients without an ileostomy (*P* = 1.000 × 10^−10^).

Regarding SDD administration, the authors compared alpha diversity between the SDD and no-SDD groups separately in the preoperative and postoperative samples using Kruskal–Wallis tests. No significant differences were observed for either the Shannon index or Phylogenetic Diversity.

### Impact of SDD administration and ileostomy status on beta diversity of the microbiome

**Fig. 3:**
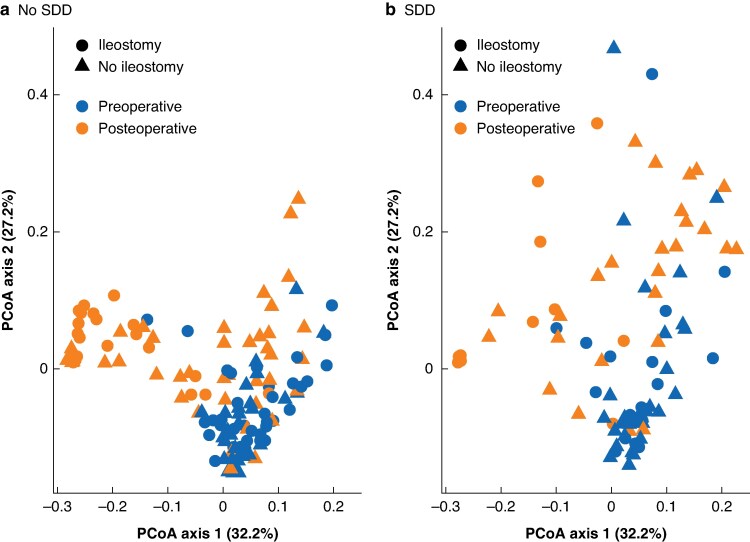
Beta diversity analysis using weighted UniFrac distances PCoA plots illustrating beta diversity changes in preoperative and postoperative samples. **a** No SDD, **b** SDD. PCoA, principal coordinates analysis; SDD, selective digestive decontamination.

Beta diversity was analysed using Weighted UniFrac distances and visualized in PCoA plots (*Fig. 3*). This analysis included samples stratified by ileostomy status, SDD administration, and time points (preoperative and postoperative). The PCoA plots revealed distinct separation between preoperative and postoperative samples along both axes (PC1 and PC2). Permutation analysis confirmed significant shifts in microbial composition over time (R^2^ = 15.6%; *P* < 0.001), with additional effects of ileostomy status (R^2^ = 9.6%; *P* < 0.001) and SDD administration (R^2^ = 3.1%; *P* = 0.002).

Beta diversity was further analysed using multilevel principal components analysis plots (*Figs S1* and *S2*) to compare microbial composition across different groups stratified by SDD administration, ileostomy status, and time points, only for those patients for whom both samples were available for analysis. These supplementary figures supported the findings from *Fig. 3*, demonstrating that surgery (preoperative *versus* postoperative samples) accounted for the largest variance in microbial composition, followed by ileostomy status. The influence of SDD administration, while significant, was less pronounced than that of the other factors.

### Dynamics in the perioperative microbiome

**Fig. 4:**
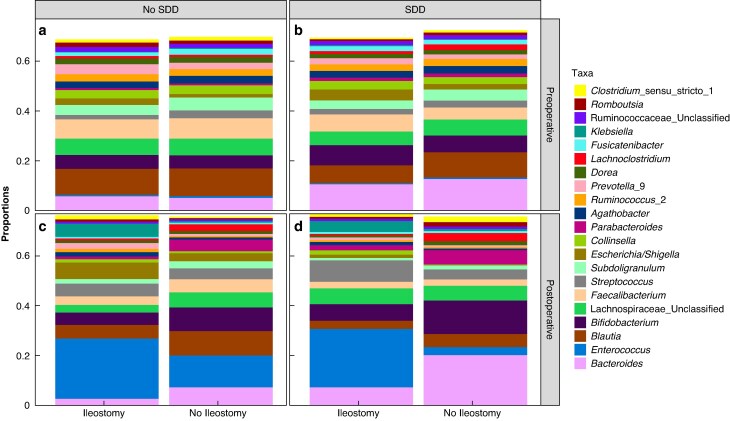
Microbiome composition of the 21 most prevalent genera Preoperative and postoperative relative abundances of the 21 most prevalent bacterial genera, stratified by ileostomy status and SDD administration. Relative abundances are displayed for preoperative faecal samples and postoperative faecal or ileostomy effluent samples. **a** Preoperative, no SDD, **b** preoperative, SDD, **c** postoperative, no SDD, **d** postoperative, SDD. SDD, selective digestive decontamination.

The microbiome composition of the 21 most prevalent genera is shown in *Fig. 4*. Relative abundances were assessed before and after surgery, stratified by postoperative sample (ileostomy content or faeces) in patients with bowel continuity and preoperative SDD administration.

### Preoperative microbiome composition

In patients without both SDD administration and ileostomy, the most abundant genus before surgery was *Blautia* (10.9%), followed by *Faecalibacterium* (8.6%) and *Lachnospiraceae* (6.6%). Similarly, in patients without SDD but with an ileostomy, *Blautia* was also the most abundant genus (10.8%), followed by *Faecalibacterium* (8.0%) and *Lachnospiraceae* (6.5%).

In patients receiving SDD without an ileostomy, *Bacteroides* was the most dominant genus (13.0%), followed by *Blautia* (10.5%) and *Bifidobacterium* (6.8%). Among patients with both an ileostomy and SDD administration, *Bacteroides* remained the most abundant genus (10.8%), followed by *Bifidobacterium* (8.4%) and *Blautia* (7.4%).

### Postoperative microbiome composition

After surgery, in patients without both SDD administration and ileostomy, *Enterococcus* became the most abundant genus (12.9%), followed by *Blautia* (9.8%) and *Bifidobacterium* (9.4%). In patients with an ileostomy but without SDD, *Enterococcus* was the most common genus (24.3%), followed by *Escherichia/Shigella* (6.8%) and *Blautia* (5.7%).

In patients receiving SDD but without an ileostomy, *Bacteroides* remained the most abundant genus after surgery (20.5%), followed by *Bifidobacterium* (13.6%) and *Parabacteroides* (5.9%). In patients with both an ileostomy and SDD, *Enterococcus* was the most abundant genus (23.5%), followed by *Streptococcus* (8.8%) and *Bacteroides* (7.4%).

### Comparative analyses of bacterial genera

**Fig. 5:**
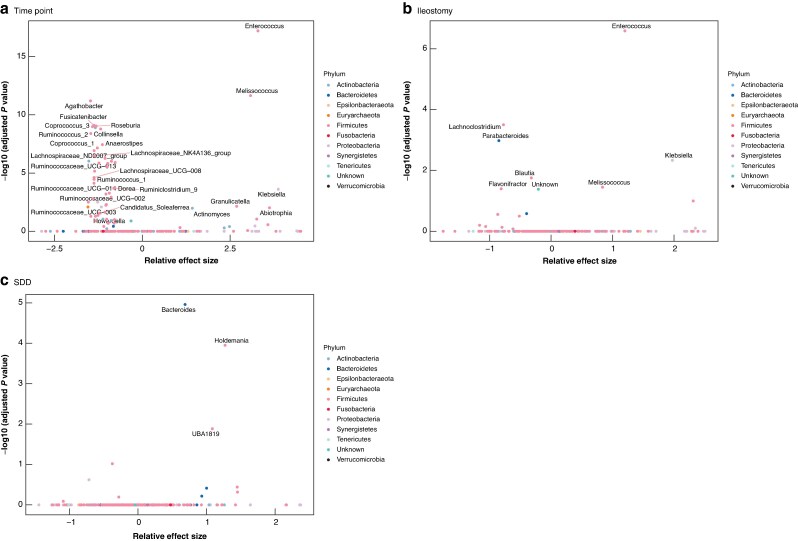
Differential abundance of bacterial genera in volcano plots **a** Preoperative *versus* postoperative genera changes, **b** ileostomy *versus* no ileostomy postoperative genera, **c** SDD *versus* no SDD postoperative genera. SDD, selective digestive decontamination.

Linear mixed models were used to test changes in the abundances of various bacterial genera associated with longitudinal time points, SDD administration, and ileostomy status (*Supplementary File  S1*). The comparison of preoperative and postoperative samples is visualized using a volcano plot (*Fig. 5*). This analysis revealed statistically significant increases in the abundances of several genera, including *Enterococcus* (adjusted *P* < 0.001), *Melissococcus* (adjusted *P* < 0.001), and *Klebsiella* (adjusted *P* < 0.001), with the highest significance values. In contrast, many genera were significantly decreased after surgery, with the majority belonging to the phylum Firmicutes. In the comparison by ileostomy status, statistically significant increases in the abundances of *Enterococcus* (adjusted *P* < 0.001), *Melissococcus* (adjusted *P* = 0.003), and *Klebsiella* (adjusted *P* < 0.001) were observed in the ileostomy group (*Fig. 5*). Conversely, significant decreases were observed for *Parabacteroides* (adjusted *P* < 0.001), *Lachnoclostridium* (adjusted *P* < 0.001), and *Flavonifractor* (adjusted *P* = 0.004) in the ileostomy group. The comparison of patients treated with SDD and those who were not treated with SDD (*Fig. 5*) showed significant increases in the abundances of *Bacteroides* (adjusted *P* < 0.001), *UBA1819* (adjusted *P* = 0.003), and *Holdemania* (adjusted *P* < 0.001) in the SDD-treated group. No significant decreases in bacterial genera were observed in this comparison.

## Discussion

This study provides a comprehensive analysis of perioperative dynamics in the human faecal microbiome in patients with rectal cancer, with stratification based on ileostomy status and SDD administration. Significant microbial shifts were observed between preoperative and postoperative samples, characterized by increases in *Enterococcus*, *Melissococcus*, and *Klebsiella,* and decreases in Firmicutes. Although SDD administration and ileostomy status also contributed, surgery itself had the largest impact on microbiome changes (that is, the pre- to postoperative comparison). Alpha diversity decreased after surgery, indicating a loss of microbial richness and evenness, and beta diversity analyses confirmed significant shifts influenced by surgery, SDD, and ileostomy status. These findings underscore the profound impact of perioperative factors on the gut microbiome, which should be considered in research on the role of microbiome in postoperative complications.

The postoperative increase in genera such as *Enterococcus* and *Klebsiella*, alongside the decrease in Firmicutes, indicates disruption of anaerobic gut bacteria, which has been described by earlier studies^19,20^. Anaerobes play a critical role in intestinal health, and their reduction after surgery may allow facultative anaerobes, such as *Enterococcus,* to proliferate. This disruption could be driven by surgery-induced inflammation, oxidative stress, and perioperative antibiotic use, and has been described by earlier studies^21^. The dominance of *Enterococcus* in both the ileostomy and the no-ileostomy groups after surgery highlights its adaptability to altered gut environments. *Enterococcus*’s ability to thrive in these environments has raised concerns about its potential to contribute to postoperative complications. Although *Enterococcus* has been associated with AL in some preclinical studies^6,22^, its presence alone likely does not directly correlate with adverse outcomes. Instead, its pathogenic potential likely depends on specific triggers, such as shifts in microbial composition, host immune responses, or other perioperative factors, which may promote pathogenic transformation in susceptible individuals^23^. Clinical efforts should focus on these interactions, not just on monitoring individual genera such as *Enterococcus*. Future studies, incorporating metagenomic and metabolomic analyses, could provide deeper insights into postoperative microbial dynamics and their impact on clinical outcomes.

The availability of both faecal and ileostomy samples in this study enabled a site-specific exploration of microbiome dynamics along the gastrointestinal tract. Whereas faecal samples predominantly reflect the distal colonic microbiota, ileostomy effluent provides access to microbial communities of the distal ileum, a region that remains underrepresented in human microbiome research. In addition to the pronounced differences observed between preoperative and postoperative samples, ileal samples also showed a clear distinction from faecal samples, reflecting distinct microbial ecosystems along the intestinal tract. In the present study, ileostomy samples were characterized by a relative dominance of the facultative anaerobe *Enterococcus* and a depletion of obligate anaerobic genera such as *Parabacteroides* and *Lachnoclostridium*. This microbial profile is consistent with the physiological characteristics of the ileal environment, including higher oxygen availability and exposure to primary bile acids that are less extensively metabolized in the absence of the colon^24^. These findings align with previous research^24,25^ showing that faecal stream diversion leads to significant shifts in microbial composition, favouring facultative anaerobes in the ileum. It should be noted, however, that ileostomy output does not fully represent the ileal microbiome under physiological conditions. Surgical diversion alters intestinal anatomy and oxygen exposure, which may further shape microbial composition. This concept is further supported by studies^26^ in small-bowel transplant recipients, in whom long-term sampling of ileal effluent via an ileostomy revealed a marked shift towards facultative anaerobes, including Enterobacteriaceae and *Lactobacilli*, with depletion of obligate anaerobic taxa. Importantly, similar microbial profiles were observed in patients with ileostomies who had not undergone a transplant, and restoration of intestinal continuity led to a reversion to a more typical anaerobe-dominated community, as assessed by colonoscopic sampling. These findings suggest that the presence of an ileostomy itself constitutes a major ecological determinant of ileal microbiome composition.

The reduction in alpha diversity after surgery indicates a loss of microbial richness and dysbiosis and was consistent across all groups, suggesting surgery as the dominant factor, with minimal additional impact from SDD. The absence of significant differences in alpha diversity between SDD and non-SDD groups contrasts with earlier findings by others^7^, which demonstrated a significant decrease in preoperative alpha diversity in patients receiving SDD. However, their analysis was limited to preoperative samples, and to the authors’ knowledge no other studies have systematically compared postoperative alpha diversity between SDD and non-SDD groups, making this a novel insight. Nonetheless, selective increases in genera such as *Bacteroides* and *Holdemania* indicate SDD-induced shifts towards opportunistic or resilient taxa^27^. Although SDD targets specific pathogens, it does not entirely preserve beneficial microbial populations, raising concerns about its long-term effects on microbial balance and resilience. These observations underscore the complexity of perioperative microbial dynamics and the need for further studies on how targeted interventions, such as SDD, interact with broader surgical and microbiome-related processes over extended postoperative periods.

Beta diversity analyses revealed distinct clustering patterns between preoperative and postoperative samples, highlighting once more the substantial impact of surgery on gut microbial composition. Permutation tests confirmed these findings with additional contributions from ileostomy status and SDD administration, though the effect of SDD was less pronounced. Whereas SDD is specifically intended to modulate gut microbial composition, the combined impact of surgery, anaesthesia, intravenous prophylactic antibiotics, fasting, and early postoperative physiology outweighs the additional perturbation induced by SDD, as assessed by 16S rRNA-based relative abundances. These findings are consistent with previous studies^28^ demonstrating extensive microbial restructuring after surgery. Interestingly, the impact of SDD administration on beta diversity, while statistically significant, mirrored its effect on alpha diversity, indicating a less pronounced influence than surgery and ileostomy status. However, this study was not designed or powered to assess clinical outcomes, and no conclusions regarding its clinical efficacy can be drawn. The modest effect on faecal community structure observed on postoperative day 4 is compatible with, but does not prove, a limited effect on clinical outcomes. The clinical efficacy of SDD should be judged primarily by clinical outcome data from adequately powered trials.

Several limitations of this study must be acknowledged. Although a large cohort of patients with rectal cancer was included, key variables such as age, sex, and comorbidities were not matched between groups, introducing potential confounding factors. Moreover, SDD exposure was associated with a higher proportion of robotic procedures (*Table 1*). The authors did not formally analyse centre characteristics, surgeon preferences, or hospital volume, and therefore cannot determine whether this reflects centre-level clustering or case selection. Residual confounding by centre or surgical approach may have thus contributed to the observed differences between the SDD and non-SDD groups. Additionally, this study did not account for other microbiome-influencing factors, such as dietary patterns or neoadjuvant radiotherapy, which may have contributed to variability in the results. Although stratifying patients into SDD and non-SDD, and ileostomy and no-ileostomy groups was insightful, the lack of randomization may have introduced selection bias. Furthermore, although the sample size was substantial, it was not sufficient to explore fully associations between microbiome changes and clinical outcomes, such as AL and infectious complications. Future analyses will utilize larger cohorts from the IMARI-trial to investigate potential links between microbiome alterations and postoperative complications in more detail. Finally, the focus on early postoperative changes leaves the long-term recovery of the microbiome and the durability of observed shifts unexplored.

In conclusion, this study provides a detailed characterization of perioperative faecal microbiome dynamics in patients with rectal cancer, highlighting significant shifts in microbial composition and diversity primarily influenced by surgery, with additional contributions from ileostomy status and SDD administration. Although the findings of this study are not directly actionable for immediate clinical decision-making, they provide an important insight into the extent of perioperative microbiome disruption following rectal surgery. The pronounced shifts in both alpha and beta diversity underscore surgery as the dominant ecological stressor, outweighing other perioperative factors. By characterizing normal postoperative microbiome dynamics in a well defined surgical cohort, these data establish a necessary baseline against which future interventions—such as dietary modulation or targeted microbial therapies—can be evaluated. Moreover, understanding these dynamics is a prerequisite for future studies that aim to link specific microbial patterns to adverse outcomes, including AL or infectious complications. The present findings should be viewed as hypothesis-generating, informing the design of adequately powered trials that can translate microbiome insights into clinically meaningful strategies. To facilitate reproducibility and further discovery, the microbiome data generated in this study are made openly available.

## Supplementary Material

zrag046_Supplementary_Data

## Data Availability

To further stimulate research in this field, the unique data set from this study will be made publicly available. Data are available at the European Nucleotide Archive, reference PRJEB83824. The study protocol is available in the supplementary material of this manuscript. The other data, including individual deidentified participant data and a data dictionary defining each field in the set, analytic methods, and study materials that support the findings of this study, are available from the corresponding author upon reasonable request. These are not publicly available due to privacy or ethical restrictions.

## Collaborators

Members of the IMARI-study group: M.D. Slooter, K. Talboom, C.P.M. van Helsdingen, A.E. Petersen, S. van Dieren, C.Y. Ponsioen (Amsterdam UMC, Amsterdam, The Netherlands); E.C.J. Consten (University Medical Centre Groningen, Groningen, the Netherlands); P.M. Verheijen, D.J. Sikkenk (Meander Medical Center, Amersfoort, The Netherlands); J.P.M. Derikx (Amsterdam UMC, Amsterdam, The Netherlands); G.D. Musters (Zaans Medical Centre, Zaandam, The Netherlands); J.D.W. van der Bilt (Flevoziekenhuis, Almere, Netherlands); A.W.H. van de Ven (Flevoziekenhuis, Almere, The Netherlands); J.G. Bloemen, J.W.A. Burger (Catharina Hospital Eindhoven, Eindhoven, The Netherlands); I. Faneyte, T. Verhagen, M.F. Lutke Holzik, I. Masselink, L. Morsink (Hospital Group Twente, Almelo, Netherlands); M. Gerhards, T.M. Karsten, S. Festen (OLVG, Amsterdam, the Netherlands); S. van Dijk (Academic Medical Center, Amsterdam, The Netherlands); W.J. de Jonge (Amsterdam University Medical Centers, Amsterdam, the Netherlands); W. van der Meij, B.J. van Wely (Bernhoven Hospital, Uden, the Netherlands); S.J. Oosterling, J. Scholten (Spaarne Gasthuis, Haarlem, the Netherlands); L.P.S. Stassen, J. Melenhorst, C.M.J. Gielen, K.M.M.W. Reynders; Y.T. van Loon, K.P. Wevers (Maastricht University Medical Center, Maastricht, the Netherlands); E.G.G. Verdaasdonk, W.J.A. Brokelman (Jeroen Bosch Hospital, 's-Hertogenbosch, the Netherlands); H.L. van Westreenen, E.J.A. Steller, A.D. van Dalsen (Isala, Zwolle, the Netherlands); J.H.W. de Wilt, L. Garms (Radboud University Medical Center, Nijmegen, the Netherlands); E.H.J. Belgers, E. Ancion (Zuyderland Medical Centre, Sittard-Geleen and Heerlen, The Netherlands); G.H.E.J. Vijgen; J. Heemskerk, J.W.A. Leijtens (St. Laurentius Hospital, Roermond, The Netherlands); W. Laméris and J.B. Tuynman (Amsterdam UMC, Amsterdam, The Netherlands)
