# Supplementary material for: Perioperative faecal microbiome dynamics in patients undergoing rectal cancer surgery in the IMARI-trial
Source: BJS Open. 2026 May 29;10(3):zrag046. doi: 10.1093/bjsopen/zrag046 (PMC13220757; doi:10.1093/bjsopen/zrag046)

**Perioperative fecal microbiome dynamics in patients undergoing rectal cancer surgery in the IMARI-trial** Kiedo Wienholts^1,2,3^, Anne E. Petersen^4,5,6,7^, Claire P.M. van Helsdingen^4,5,6,7^, Kevin Talboom^1,2,3^, Mark Davids^8^, Johannes H.W. de Wilt^9^, Wouter J. de Jonge^5,6,10^, Pieter J. Tanis^1,2,3,11^, Roel Hompes^1,2,3^, Joep P.M. Derikx^4,6,7^ on behalf of the IMARI-study group

## Affiliations:

^1.^ Department of Surgery, Amsterdam UMC location University of Amsterdam, Meibergdreef 9, Amsterdam, The Netherlands

^2.^ Cancer Center Amsterdam, Treatment and Quality of Life, Amsterdam, The Netherlands

^3.^ Cancer Center Amsterdam, Imaging and Biomarkers, Amsterdam, The Netherlands

^4.^ Department of Paediatric Surgery, Emma Children’s Hospital, Amsterdam UMC, location University of Amsterdam, Meibergdreef 9, Amsterdam, The Netherlands

^5.^ Tytgat Institute for Liver and Intestinal Research, Amsterdam UMC, location University of Amsterdam, Meibergdreef 9, Amsterdam, The Netherlands

^6.^ Amsterdam Gastroenterology Endocrinology Metabolism, Amsterdam, The Netherlands

^7.^ Amsterdam Reproduction and Development, Amsterdam, The Netherlands

^8.^ Department of Experimental Vascular Medicine, Amsterdam UMC location University of Amsterdam, Meibergdreef 9, Amsterdam, The Netherlands.

^9.^ Department of Surgery, Radboud university medical centre, Radboud Institute for Health Sciences, Nijmegen, the Netherlands

^10.^ Department of Surgery, University Hospital Bonn, Bonn, Germany

^11.^ Department of Surgical Oncology and Gastrointestinal Surgery, Erasmus MC, Rotterdam, the Netherlands

**Corresponding author:** Drs. K. Wienholts M.D., [k.r.wienholts@amsterdamumc.nl.](mailto:k.r.wienholts@amsterdamumc.nl) Department of Surgery, Amsterdam UMC location University of Amsterdam, Meibergdreef 9, 1105 AZ, Amsterdam, the Netherlands. PO Box 22660, 1100 DD Amsterdam, Phone: +31 20 566 9111. ORCID-ID: 0000-0002-3898-

1663

**Supplementary Materials – Index**

**Supplementary material**

| **Supplementary figures** |  |
| --- | --- |
| Supplementary figure S1 | *page 3* |
| Supplementary figure S2 | *page 4* |
| **Supplementary files** |  |
| Supplementary file S1 | *page 5* |
| **Study protocol (published version)** | *page 6* |
| **Study protocol (complete version)** | *page 16* |

**Supplementary figures**

## Supplementary figure S1. Multilevel PCA – SDD vs. no SDD groups

Beta diversity differences between SDD-treated and non-SDD-treated groups visualized using multilevel Principal Components Analysis (PCA).


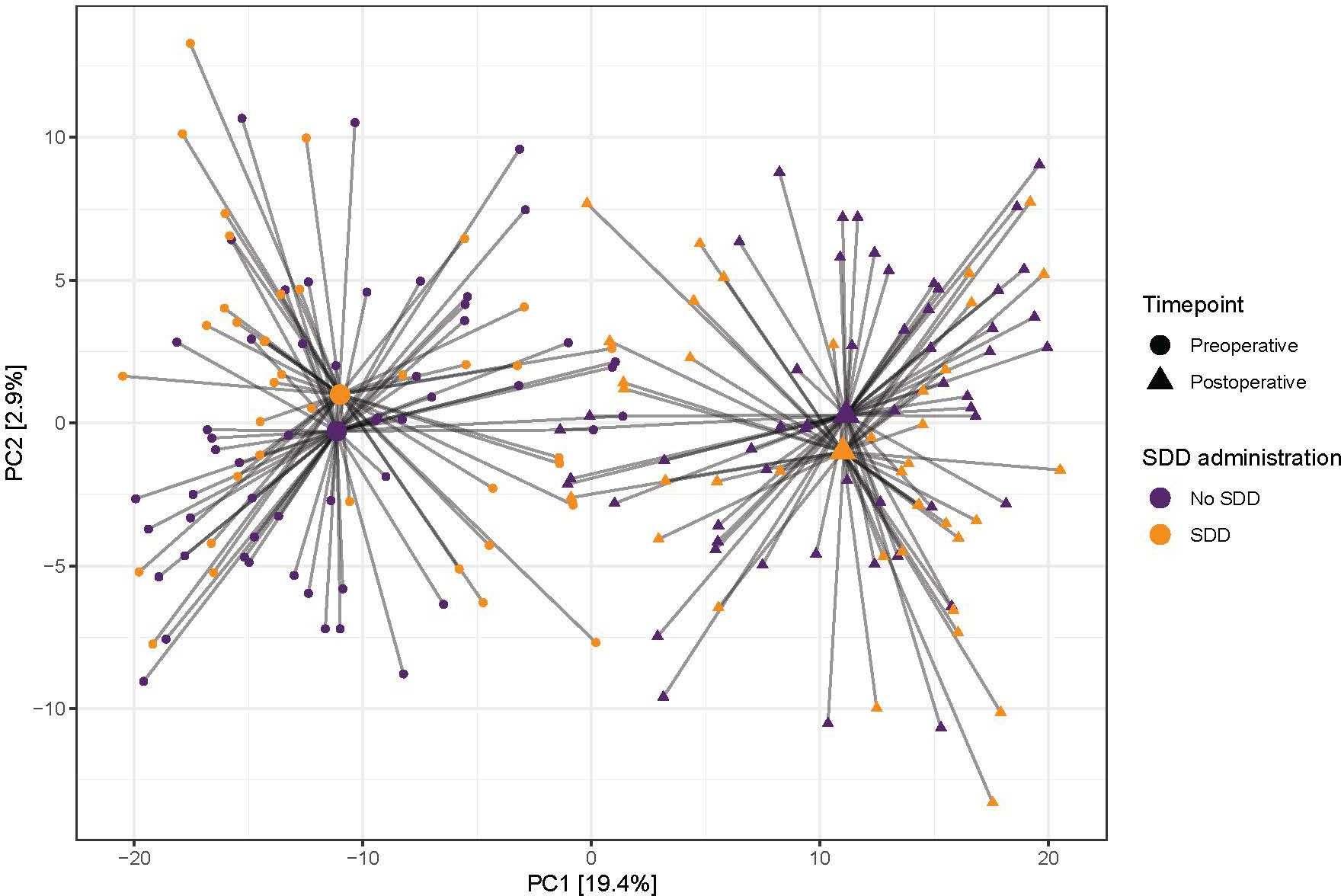


SDD = Selective Digestive Decontamination

## Supplementary figure S2. Multilevel PCA – Ileostomy vs. no ileostomy groups

Beta diversity differences between ileostomy and no ileostomy groups visualized using multilevel PCA.


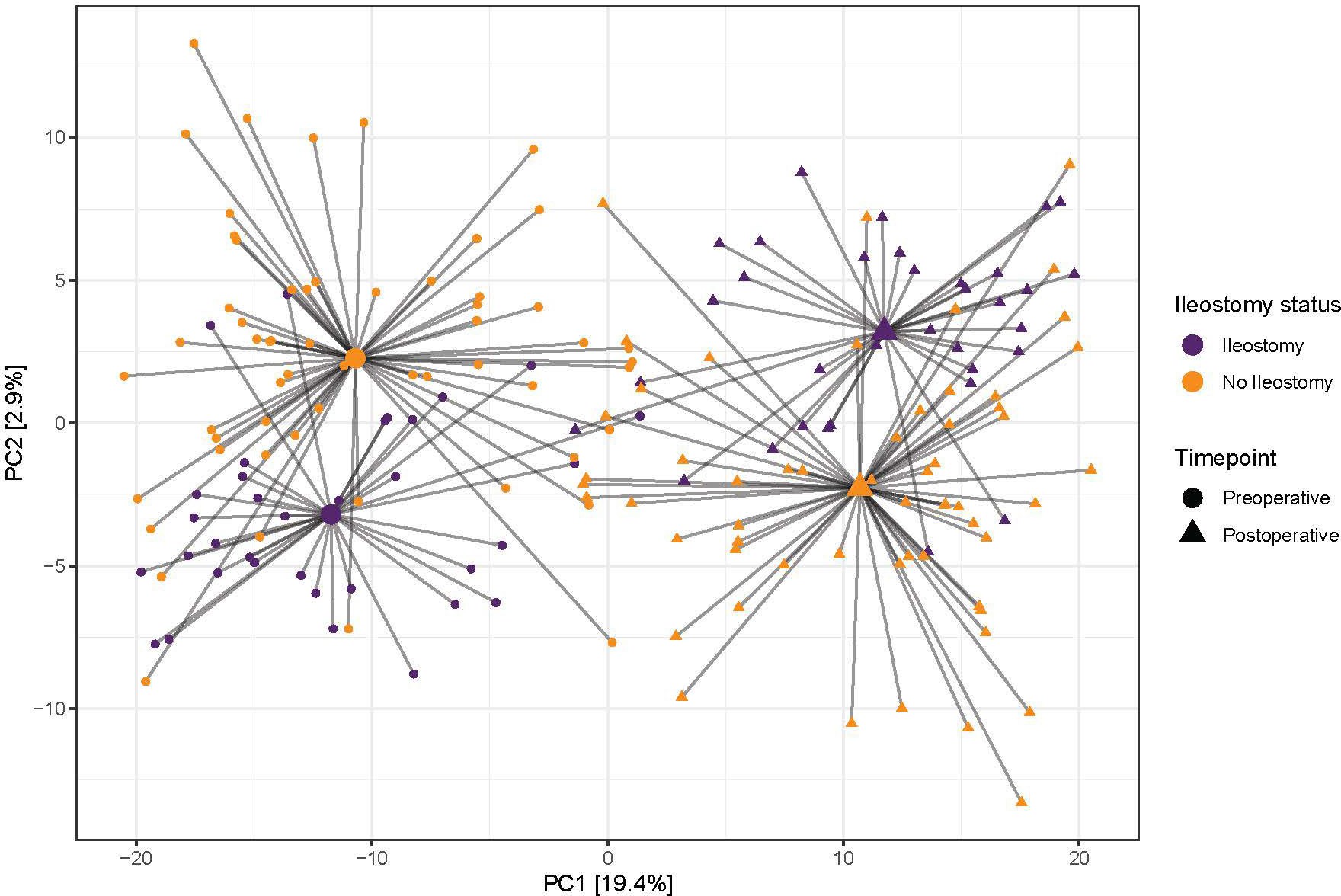


| **Estimate_.Intercept.** | **pvalue_.Intercept.** | **Estimate_Time_Point2** | **pvalue_Time_Point2** | **Estimate_Time_Point2.SDDSDD** | **pvalue_Time_Point2.SDDSDD** | **Estimate_Time_Point2.IleostomyNo.Ileostomy** | **pvalue_Time_Point2.IleostomyNo.Ileostomy** | **padj_.Intercept.** | **padj_Time_Point2** | **padj_Time_Point2.SDDSDD** | **padj_Time_Point2.IleostomyNo.Ileostomy** | **Kingdom** | **Phylum** | **Class** | **Order** | **Family** | **Genus** | **unique** | **mean_abundance** |
| --- | --- | --- | --- | --- | --- | --- | --- | --- | --- | --- | --- | --- | --- | --- | --- | --- | --- | --- | --- |
| 0,127759572 | 2,16015E-21 | -0,031280265 | 0,000655143 | -0,004086779 | 0,437522746 | 0,020211867 | 0,00017171 | 5,16275E-19 | 0,130373389 | 1 | 0,041725458 | Unknown | Unknown | Unknown | Unknown | Unknown | Unknown | Unknown | 0,097584211 |
| 0,066111176 | 0,004176431 | -0,028698665 | 0,09581916 | 0,060787731 | 4,38954E-08 | 0,03525263 | 0,001092926 | 0,684934707 | 1 | 1,09738E-05 | 0,263395054 | Bacteria | Bacteroidetes | Bacteroidia | Bacteroidales | Bacteroidaceae | Bacteroides | Bacteroides | 0,088546491 |
| -0,218292631 | 1,66062E-08 | 0,284357976 | 2,45712E-20 | -0,031710146 | 0,051149596 | -0,103753692 | 1,03766E-09 | 3,35445E-06 | 6,14279E-18 | 1 | 2,59416E-07 | Bacteria | Firmicutes | Bacilli | Lactobacillales | Enterococcaceae | Enterococcus | Enterococcus | 0,086423246 |
| 0,158326308 | 6,34598E-22 | -0,062085031 | 5,28049E-09 | -0,018251505 | 0,004143554 | 0,025789073 | 7,15202E-05 | 1,52304E-19 | 1,23563E-06 | 0,990309496 | 0,017593959 | Bacteria | Firmicutes | Clostridia | Clostridiales | Lachnospiraceae | Blautia | Blautia | 0,080280702 |
| 0,036850995 | 0,108507799 | 0,002418445 | 0,887481415 | 0,024755216 | 0,013720087 | 0,019349858 | 0,054604975 | 1 | 1 | 1 | 1 | Bacteria | Actinobacteria | Actinobacteria | Bifidobacteriales | Bifidobacteriaceae | Bifidobacterium | Bifidobacterium | 0,070773684 |
| 0,118674159 | 5,5167E-24 | -0,045935459 | 4,14053E-09 | -0,014654434 | 0,002656164 | 0,009252141 | 0,056570239 | 1,34607E-21 | 9,73024E-07 | 0,640135639 | 1 | Bacteria | Firmicutes | Clostridia | Clostridiales | Ruminococcaceae | Faecalibacterium | Faecalibacterium | 0,052235965 |
| -0,014662558 | 0,405788694 | 0,040225339 | 0,002554995 | 0,009368996 | 0,222339759 | -0,013121617 | 0,090160074 | 1 | 0,48544903 | 1 | 1 | Bacteria | Firmicutes | Bacilli | Lactobacillales | Streptococcaceae | Streptococcus | Streptococcus | 0,037502632 |
| 0,073647395 | 9,93452E-21 | -0,032150837 | 2,32279E-09 | -0,005401716 | 0,092898649 | 0,007283353 | 0,024644174 | 2,35448E-18 | 5,505E-07 | 1 | 1 | Bacteria | Firmicutes | Clostridia | Clostridiales | Ruminococcaceae | Subdoligranulum | Subdoligranulum | 0,030320614 |
| 0,008205936 | 0,643091216 | 0,031676543 | 0,017385717 | -0,017100879 | 0,026863839 | -0,01771327 | 0,022774715 | 1 | 1 | 1 | 1 | Bacteria | Proteobacteria | Gammaproteobacteria | Enterobacteriales | Enterobacteriaceae | Escherichia/Shigella | Escherichia/Shigella | 0,029300877 |
| -0,018708089 | 0,037117889 | 0,015226934 | 0,023368359 | 0,004828182 | 0,218535774 | 0,018834043 | 4,24611E-06 | 1 | 1 | 1 | 0,001053034 | Bacteria | Bacteroidetes | Bacteroidia | Bacteroidales | Tannerellaceae | Parabacteroides | Parabacteroides | 0,022170614 |
| 0,06588058 | 1,04859E-26 | -0,029347489 | 5,18444E-12 | -0,003023817 | 0,196918191 | 0,000257776 | 0,912810718 | 2,60051E-24 | 1,265E-09 | 1 | 1 | Bacteria | Actinobacteria | Coriobacteriia | Coriobacteriales | Coriobacteriaceae | Collinsella | Collinsella | 0,021941228 |
| 0,056970991 | 4,68441E-28 | -0,027422129 | 2,55769E-14 | -0,000563252 | 0,773349455 | 0,001880356 | 0,339911124 | 1,1711E-25 | 6,34306E-12 | 1 | 1 | Bacteria | Firmicutes | Clostridia | Clostridiales | Lachnospiraceae | Agathobacter | Agathobacter | 0,018583772 |
| 0,056889359 | 4,75404E-21 | -0,027193028 | 1,66244E-11 | 0,00060413 | 0,787182332 | 0,00082056 | 0,715472507 | 1,13146E-18 | 4,02311E-09 | 1 | 1 | Bacteria | Firmicutes | Clostridia | Clostridiales | Ruminococcaceae | Ruminococcus_2 | Ruminococcus_2 | 0,018542544 |
| 0,049105165 | 1,55535E-05 | -0,018364987 | 0,024549062 | -0,002347466 | 0,660226796 | -0,002556637 | 0,633313408 | 0,003017379 | 1 | 1 | 1 | Bacteria | Bacteroidetes | Bacteroidia | Bacteroidales | Prevotellaceae | Prevotella_9 | Prevotella_9 | 0,018338596 |
| 0,034532298 | 1,33989E-16 | -0,014144531 | 8,05557E-07 | 0,001095035 | 0,49755734 | 0,003836267 | 0,019216627 | 3,06834E-14 | 0,000182056 | 1 | 1 | Bacteria | Firmicutes | Clostridia | Clostridiales | Lachnospiraceae | Dorea | Dorea | 0,017965351 |
| 0,000620704 | 0,906796254 | 0,001823527 | 0,648320438 | 0,003789231 | 0,1242298 | 0,012417633 | 1,2678E-06 | 1 | 1 | 1 | 0,000315682 | Bacteria | Firmicutes | Clostridia | Clostridiales | Lachnospiraceae | Lachnoclostridium | Lachnoclostridium | 0,016017105 |
| 0,039401237 | 2,80242E-24 | -0,01891808 | 3,23769E-12 | -6,16839E-05 | 0,967017234 | 0,002021012 | 0,179175222 | 6,92198E-22 | 7,9971E-10 | 1 | 1 | Bacteria | Firmicutes | Clostridia | Clostridiales | Lachnospiraceae | Fusicatenibacter | Fusicatenibacter | 0,013722807 |
| -0,037126153 | 0,009238287 | 0,052956167 | 1,09962E-06 | -0,004713413 | 0,44319281 | -0,026998819 | 1,88765E-05 | 1 | 0,000246316 | 1 | 0,004662494 | Bacteria | Proteobacteria | Gammaproteobacteria | Enterobacteriales | Enterobacteriaceae | Klebsiella | Klebsiella | 0,013685965 |
| 0,017869472 | 0,028690451 | -0,005286411 | 0,384128542 | 0,002034441 | 0,568050115 | 0,000729342 | 0,838742794 | 1 | 1 | 1 | 1 | Bacteria | Firmicutes | Clostridia | Clostridiales | Peptostreptococcaceae | Romboutsia | Romboutsia | 0,011978509 |
| 0,009148105 | 0,300530761 | -0,001144501 | 0,862274082 | 0,002751681 | 0,472751462 | 0,001478335 | 0,701414619 | 1 | 1 | 1 | 1 | Bacteria | Firmicutes | Clostridia | Clostridiales | Clostridiaceae_1 | Clostridium_sensu_stricto_1 | Clostridium_sensu_stricto_1 | 0,010295175 |
| 0,026278729 | 2,83168E-24 | -0,011500993 | 1,50271E-10 | -0,001283959 | 0,197766252 | 0,001395614 | 0,16438965 | 6,96592E-22 | 3,62153E-08 | 1 | 1 | Bacteria | Firmicutes | Clostridia | Clostridiales | Lachnospiraceae | Anaerostipes | Anaerostipes | 0,010117105 |
| 0,011091222 | 0,00266018 | -0,003964649 | 0,156307508 | 0,000580305 | 0,760652007 | 0,004443992 | 0,020973919 | 0,449570501 | 1 | 1 | 1 | Bacteria | Bacteroidetes | Bacteroidia | Bacteroidales | Rikenellaceae | Alistipes | Alistipes | 0,009627193 |
| 0,023127911 | 1,21868E-13 | -0,009316827 | 2,32578E-05 | -0,000321479 | 0,827428259 | -0,00052944 | 0,720362274 | 2,70546E-11 | 0,00504694 | 1 | 1 | Bacteria | Firmicutes | Clostridia | Clostridiales | Christensenellaceae | Christensenellaceae_R-7_group | Christensenellaceae_R-7_group | 0,009113158 |
| 0,025584706 | 3,37824E-27 | -0,011337816 | 3,77128E-12 | -0,003228618 | 0,000387636 | 0,001454596 | 0,108210503 | 8,41182E-25 | 9,27735E-10 | 0,095746013 | 1 | Bacteria | Firmicutes | Clostridia | Clostridiales | Lachnospiraceae | Roseburia | Roseburia | 0,008626754 |
| 0,018682555 | 1,44109E-17 | -0,007384195 | 2,38348E-06 | -0,000952523 | 0,414751471 | 0,000284774 | 0,80743606 | 3,31451E-15 | 0,000531515 | 1 | 1 | Bacteria | Firmicutes | Clostridia | Clostridiales | Ruminococcaceae | Ruminococcaceae_UCG-002 | Ruminococcaceae_UCG-002 | 0,007858333 |
| 0,021520843 | 9,7174E-16 | -0,009709507 | 3,29145E-07 | 0,001019762 | 0,402029173 | -0,001303062 | 0,286275864 | 2,18641E-13 | 7,47159E-05 | 1 | 1 | Bacteria | Firmicutes | Clostridia | Clostridiales | Ruminococcaceae | Ruminococcaceae_UCG-014 | Ruminococcaceae_UCG-014 | 0,007049561 |
| -0,000708042 | 0,886557435 | 0,003008244 | 0,417767341 | 0,000271935 | 0,899531106 | 0,003757027 | 0,08411835 | 1 | 1 | 1 | 1 | Bacteria | Firmicutes | Clostridia | Clostridiales | Peptostreptococcaceae | Intestinibacter | Intestinibacter | 0,007027193 |
| -0,00417833 | 0,452474108 | 0,007311324 | 0,080554746 | -0,005507135 | 0,027168413 | 0,003743123 | 0,132685656 | 1 | 1 | 1 | 1 | Bacteria | Firmicutes | Bacilli | Lactobacillales | Lactobacillaceae | Lactobacillus | Lactobacillus | 0,006510965 |
| 0,017644227 | 2,23714E-16 | -0,008051808 | 1,5384E-07 | -0,001870195 | 0,031193351 | 0,001060855 | 0,22300454 | 5,10068E-14 | 3,50754E-05 | 1 | 1 | Bacteria | Firmicutes | Clostridia | Clostridiales | Ruminococcaceae | Ruminococcus_1 | Ruminococcus_1 | 0,005860526 |
| 0,013068996 | 3,57986E-09 | -0,005337882 | 0,001052249 | -0,001703062 | 0,129208634 | 0,001209031 | 0,281835491 | 7,44611E-07 | 0,20624088 | 1 | 1 | Bacteria | Firmicutes | Negativicutes | Selenomonadales | Acidaminococcaceae | Phascolarctobacterium | Phascolarctobacterium | 0,005493421 |
| 0,008586866 | 0,002855663 | -0,004950125 | 0,024660946 | 0,002260562 | 0,147645958 | 0,003128807 | 0,046116875 | 0,479751438 | 1 | 1 | 1 | Bacteria | Verrucomicrobia | Verrucomicrobiae | Verrucomicrobiales | Akkermansiaceae | Akkermansia | Akkermansia | 0,005441667 |
| 0,00838 | 0,000849711 | -0,003594692 | 0,058006251 | 0,003551514 | 0,006275675 | 0,000240981 | 0,851563358 | 0,152947943 | 1 | 1 | 1 | Bacteria | Firmicutes | Erysipelotrichia | Erysipelotrichales | Erysipelotrichaceae | Holdemanella | Holdemanella | 0,005279825 |
| 0,015129526 | 7,21699E-20 | -0,007124574 | 4,96663E-10 | -1,58319E-05 | 0,981089701 | 0,000407801 | 0,54381343 | 1,68878E-17 | 1,18703E-07 | 1 | 1 | Bacteria | Firmicutes | Clostridia | Clostridiales | Lachnospiraceae | Lachnospiraceae_ND3007_group | Lachnospiraceae_ND3007_group | 0,005191228 |
| 0,01465096 | 1,77962E-22 | -0,007100928 | 4,24945E-12 | -0,000401775 | 0,510633772 | 0,000959476 | 0,118973538 | 4,30667E-20 | 1,04111E-09 | 1 | 1 | Bacteria | Firmicutes | Clostridia | Clostridiales | Lachnospiraceae | Coprococcus_3 | Coprococcus_3 | 0,005008333 |
| 0,015328211 | 1,01994E-10 | -0,007320697 | 1,44441E-05 | -0,000638289 | 0,508991544 | 0,000441598 | 0,649604897 | 2,21328E-08 | 0,003148808 | 1 | 1 | Bacteria | Firmicutes | Clostridia | Clostridiales | Lachnospiraceae | Coprococcus_2 | Coprococcus_2 | 0,004760088 |
| 0,00933343 | 1,30249E-09 | -0,003500117 | 0,001026403 | -0,000912988 | 0,142269544 | 0,000696714 | 0,264409496 | 2,72221E-07 | 0,20220134 | 1 | 1 | Bacteria | Firmicutes | Clostridia | Clostridiales | Lachnospiraceae | Lachnospira | Lachnospira | 0,004354825 |
| -0,010311833 | 0,080132728 | 0,0138415 | 0,00182906 | -0,003009983 | 0,238187378 | -0,004337211 | 0,091995881 | 1 | 0,354837578 | 1 | 1 | Bacteria | Proteobacteria | Gammaproteobacteria | Enterobacteriales | Enterobacteriaceae | Citrobacter | Citrobacter | 0,004317105 |
| 0,012715369 | 1,58855E-20 | -0,005848579 | 1,39863E-09 | -0,000981281 | 0,069136232 | 0,000686987 | 0,205292995 | 3,74897E-18 | 3,32874E-07 | 1 | 1 | Bacteria | Firmicutes | Clostridia | Clostridiales | Lachnospiraceae | Lachnospiraceae_NK4A136_group | Lachnospiraceae_NK4A136_group | 0,004294298 |
| 0,003705503 | 0,152193137 | -0,001263515 | 0,51399721 | 0,001869097 | 0,105744798 | 0,001345924 | 0,24588892 | 1 | 1 | 1 | 1 | Bacteria | Firmicutes | Clostridia | Clostridiales | Ruminococcaceae | Ruminiclostridium_5 | Ruminiclostridium_5 | 0,004072807 |
| -0,002270253 | 0,609247834 | 0,009265174 | 0,007272109 | 0,00261909 | 0,405655935 | -0,009332534 | 0,003382482 | 1 | 1 | 1 | 0,794883322 | Bacteria | Proteobacteria | Gammaproteobacteria | Enterobacteriales | Enterobacteriaceae | Enterobacter | Enterobacter | 0,003977193 |
| 0,012598266 | 9,13254E-10 | -0,005288543 | 0,000252237 | -0,000750646 | 0,373071624 | -0,000643817 | 0,447277949 | 1,91783E-07 | 0,051960725 | 1 | 1 | Bacteria | Firmicutes | Negativicutes | Selenomonadales | Veillonellaceae | Dialister | Dialister | 0,003963596 |
| 0,008334987 | 7,7609E-20 | -0,003559745 | 1,26864E-08 | -0,000621124 | 0,094622441 | 0,000818645 | 0,028868917 | 1,80829E-17 | 2,93056E-06 | 1 | 1 | Bacteria | Firmicutes | Clostridia | Clostridiales | Lachnospiraceae | Marvinbryantia | Marvinbryantia | 0,003563596 |
| 0,00763582 | 1,42873E-12 | -0,003251398 | 2,66838E-05 | -0,000352601 | 0,479522422 | 0,000430934 | 0,38940807 | 3,1432E-10 | 0,005763698 | 1 | 1 | Bacteria | Firmicutes | Clostridia | Clostridiales | Ruminococcaceae | Ruminococcaceae_NK4A214_group | Ruminococcaceae_NK4A214_group | 0,003136842 |
| -0,003353005 | 0,161002705 | 0,002342341 | 0,194765831 | 0,000715753 | 0,528029381 | 0,003188982 | 0,005890909 | 1 | 1 | 1 | 1 | Bacteria | Firmicutes | Clostridia | Clostridiales | Lachnospiraceae | Sellimonas | Sellimonas | 0,003088158 |
| 0,010031348 | 0,198310505 | -0,003329091 | 0,567340749 | -0,001282054 | 0,704338121 | -0,001833808 | 0,589760401 | 1 | 1 | 1 | 1 | Bacteria | Proteobacteria | Gammaproteobacteria | Aeromonadales | Aeromonadaceae | Aeromonas | Aeromonas | 0,002922368 |
| -0,004458967 | 0,061100313 | 0,003144874 | 0,077535325 | -0,00015215 | 0,884727 | 0,003220585 | 0,002667967 | 1 | 1 | 1 | 0,634976214 | Bacteria | Firmicutes | Clostridia | Clostridiales | Lachnospiraceae | Hungatella | Hungatella | 0,002769737 |
| 0,006906522 | 5,01446E-16 | -0,002809688 | 2,87297E-06 | -0,000149405 | 0,700671123 | -9,3914E-05 | 0,809675754 | 1,13327E-13 | 0,0006378 | 1 | 1 | Bacteria | Firmicutes | Clostridia | Clostridiales | Ruminococcaceae | Ruminococcaceae_UCG-005 | Ruminococcaceae_UCG-005 | 0,002712719 |
| 0,007800692 | 1,16207E-17 | -0,003595457 | 2,97239E-08 | -0,000162896 | 0,654334976 | 0,000165974 | 0,650365227 | 2,68438E-15 | 6,83649E-06 | 1 | 1 | Bacteria | Firmicutes | Clostridia | Clostridiales | Ruminococcaceae | Ruminococcaceae_UCG-013 | Ruminococcaceae_UCG-013 | 0,002648684 |
| -0,005433617 | 0,118817907 | 0,007415811 | 0,004653372 | 0,000293716 | 0,845550115 | -0,003477321 | 0,022718671 | 1 | 0,870180601 | 1 | 1 | Bacteria | Firmicutes | Negativicutes | Selenomonadales | Veillonellaceae | Veillonella | Veillonella | 0,002474123 |
| 0,003894656 | 2,61039E-06 | -0,001924155 | 0,001941982 | 0,000979506 | 0,020909353 | 0,00081339 | 0,055173326 | 0,000509026 | 0,374802442 | 1 | 1 | Bacteria | Bacteroidetes | Bacteroidia | Bacteroidales | Barnesiellaceae | Barnesiella | Barnesiella | 0,002337281 |
| -0,002643216 | 0,32705124 | 0,002784006 | 0,168433994 | 0,001359671 | 0,252106194 | 0,000131345 | 0,912270334 | 1 | 1 | 1 | 1 | Bacteria | Bacteroidetes | Bacteroidia | Bacteroidales | Marinifilaceae | Odoribacter | Odoribacter | 0,002259211 |
| 0,005484402 | 1,05627E-23 | -0,002191891 | 6,42384E-09 | -0,000427504 | 0,043710304 | 0,000174864 | 0,410650724 | 2,56672E-21 | 1,49033E-06 | 1 | 1 | Bacteria | Firmicutes | Clostridia | Clostridiales | Ruminococcaceae | Butyricicoccus | Butyricicoccus | 0,002224123 |
| 0,005468634 | 3,73543E-10 | -0,002198521 | 0,000449767 | -0,000726335 | 0,054517661 | 0,000322656 | 0,393278642 | 7,95646E-08 | 0,091752515 | 1 | 1 | Bacteria | Firmicutes | Erysipelotrichia | Erysipelotrichales | Erysipelotrichaceae | Erysipelotrichaceae_UCG-003 | Erysipelotrichaceae_UCG-003 | 0,002171053 |
| 0,004645372 | 0,015114971 | -0,002376636 | 0,094963961 | 0,001047825 | 0,210448643 | 0,000366318 | 0,662722305 | 1 | 1 | 1 | 1 | Bacteria | Bacteroidetes | Bacteroidia | Bacteroidales | Prevotellaceae | Paraprevotella | Paraprevotella | 0,00210307 |
| 0,005532159 | 1,47574E-10 | -0,002789226 | 7,085E-06 | 0,000398059 | 0,264421508 | 0,000311778 | 0,384563986 | 3,1876E-08 | 0,001558699 | 1 | 1 | Bacteria | Firmicutes | Clostridia | Clostridiales | Lachnospiraceae | CAG-56 | CAG-56 | 0,001988158 |
| 0,00373558 | 0,000340362 | -0,001656788 | 0,0339706 | 0,000130069 | 0,795538513 | 0,000615574 | 0,222933882 | 0,063307382 | 1 | 1 | 1 | Bacteria | Firmicutes | Clostridia | Clostridiales | Peptostreptococcaceae | Terrisporobacter | Terrisporobacter | 0,001964035 |
| 0,00341558 | 0,055664205 | -0,000801716 | 0,554285978 | -0,000934575 | 0,302529335 | 0,000195292 | 0,82973401 | 1 | 1 | 1 | 1 | Bacteria | Bacteroidetes | Bacteroidia | Bacteroidales | Prevotellaceae | Prevotella_2 | Prevotella_2 | 0,001921491 |
| -0,004620051 | 0,199572758 | 0,005813935 | 0,03144659 | -0,00066677 | 0,669730027 | -0,001816411 | 0,249139241 | 1 | 1 | 1 | 1 | Bacteria | Proteobacteria | Gammaproteobacteria | Enterobacteriales | Enterobacteriaceae | Serratia | Serratia | 0,001862719 |
| 0,002952799 | 0,000168398 | -0,000550475 | 0,358919879 | -0,000633992 | 0,202492387 | -0,000142306 | 0,774577355 | 0,032163949 | 1 | 1 | 1 | Bacteria | Firmicutes | Clostridia | Clostridiales | Ruminococcaceae | Ruminiclostridium_6 | Ruminiclostridium_6 | 0,001710965 |
| 0,004886589 | 4,85221E-22 | -0,00217772 | 2,92399E-10 | -0,00031734 | 0,120233254 | 0,00014705 | 0,472345172 | 1,16938E-19 | 7,01757E-08 | 1 | 1 | Bacteria | Firmicutes | Clostridia | Clostridiales | Lachnospiraceae | Coprococcus_1 | Coprococcus_1 | 0,001699561 |
| 0,004681855 | 0,001436095 | -0,001915271 | 0,080380317 | -0,000217469 | 0,748770378 | -0,00011495 | 0,866208445 | 0,252752794 | 1 | 1 | 1 | Bacteria | Bacteroidetes | Bacteroidia | Bacteroidales | Prevotellaceae | Alloprevotella | Alloprevotella | 0,001687281 |
| 0,003589694 | 8,4719E-10 | -0,001430475 | 0,000590987 | -0,000541286 | 0,045739057 | 0,00050184 | 0,064575308 | 1,78757E-07 | 0,118788482 | 1 | 1 | Bacteria | Actinobacteria | Coriobacteriia | Coriobacteriales | Eggerthellaceae | Senegalimassilia | Senegalimassilia | 0,001614035 |
| -0,004534494 | 0,154949344 | 0,006001124 | 0,012205914 | 0,001197093 | 0,386262233 | -0,003769577 | 0,007124181 | 1 | 1 | 1 | 1 | Bacteria | Firmicutes | Bacilli | Bacillales | Staphylococcaceae | Staphylococcus | Staphylococcus | 0,001577632 |
| -0,001067136 | 0,242888632 | 0,000358773 | 0,600531269 | 0,001704923 | 5,25656E-05 | 0,001363476 | 0,001166694 | 1 | 1 | 0,013036263 | 0,280006441 | Bacteria | Firmicutes | Clostridia | Clostridiales | Ruminococcaceae | UBA1819 | UBA1819 | 0,001572807 |
| -0,002378726 | 0,546584249 | 0,000742248 | 0,801165255 | 0,002564302 | 0,135514871 | 0,001675781 | 0,331422869 | 1 | 1 | 1 | 1 | Bacteria | Proteobacteria | Gammaproteobacteria | Enterobacteriales | Enterobacteriaceae | Morganella | Morganella | 0,001564474 |
| 0,003470377 | 0,009244815 | -0,000867982 | 0,380590652 | -4,06425E-05 | 0,943576818 | -0,000829849 | 0,152010186 | 1 | 1 | 1 | 1 | Bacteria | Bacteroidetes | Bacteroidia | Bacteroidales | Prevotellaceae | Prevotella_7 | Prevotella_7 | 0,00147807 |
| 0,004726929 | 0,000438141 | -0,002306853 | 0,020684216 | 0,000354212 | 0,538339 | -0,000151343 | 0,793862805 | 0,080617918 | 1 | 1 | 1 | Bacteria | Firmicutes | Erysipelotrichia | Erysipelotrichales | Erysipelotrichaceae | Catenibacterium | Catenibacterium | 0,001455263 |
| -0,002345164 | 0,315881431 | 0,004687288 | 0,007732293 | -0,001522629 | 0,134028286 | -0,002484355 | 0,015554406 | 1 | 1 | 1 | 1 | Bacteria | Proteobacteria | Gammaproteobacteria | Pasteurellales | Pasteurellaceae | Haemophilus | Haemophilus | 0,001455263 |
| 0,003174857 | 2,71338E-15 | -0,001289979 | 5,41457E-06 | -0,000152361 | 0,403007332 | 0,000248645 | 0,174643939 | 6,07798E-13 | 0,00119662 | 1 | 1 | Bacteria | Firmicutes | Clostridia | Clostridiales | Ruminococcaceae | Intestinimonas | Intestinimonas | 0,001452632 |
| 0,004422093 | 0,070902118 | -0,002174887 | 0,238186129 | 0,000307356 | 0,788380875 | -6,91224E-05 | 0,952096572 | 1 | 1 | 1 | 1 | Bacteria | Bacteroidetes | Bacteroidia | Bacteroidales | Porphyromonadaceae | Porphyromonas | Porphyromonas | 0,00142193 |
| 0,0041606 | 4,73843E-16 | -0,002108233 | 3,98545E-09 | -8,73667E-05 | 0,669785886 | 0,000377519 | 0,068435641 | 1,07562E-13 | 9,40567E-07 | 1 | 1 | Bacteria | Actinobacteria | Coriobacteriia | Coriobacteriales | Eggerthellaceae | Enterorhabdus | Enterorhabdus | 0,001384211 |
| 0,003686371 | 3,14596E-24 | -0,001608908 | 6,77297E-12 | -0,000196886 | 0,175734445 | 0,000126236 | 0,385758656 | 7,70761E-22 | 1,64583E-09 | 1 | 1 | Bacteria | Firmicutes | Clostridia | Clostridiales | Lachnospiraceae | Lachnospiraceae_FCS020_group | Lachnospiraceae_FCS020_group | 0,001356579 |
| -0,003917261 | 0,340764436 | 0,00439994 | 0,15280584 | 0,003180906 | 0,075400571 | -0,003343469 | 0,063424283 | 1 | 1 | 1 | 1 | Bacteria | Proteobacteria | Gammaproteobacteria | Xanthomonadales | Xanthomonadaceae | Stenotrophomonas | Stenotrophomonas | 0,001348684 |
| 0,003404499 | 3,59411E-09 | -0,00147974 | 0,000452214 | -0,000481488 | 0,076733421 | 0,000373998 | 0,169995594 | 7,44611E-07 | 0,091799542 | 1 | 1 | Bacteria | Actinobacteria | Coriobacteriia | Coriobacteriales | Eggerthellaceae | Slackia | Slackia | 0,001320614 |
| 0,003089449 | 4,9797E-13 | -0,001043709 | 0,000495823 | -9,6972E-05 | 0,588708869 | -0,000243177 | 0,178515399 | 1,10051E-10 | 0,100156152 | 1 | 1 | Bacteria | Firmicutes | Clostridia | Clostridiales | Family_XIII | Family_XIII_AD3011_group | Family_XIII_AD3011_group | 0,001320614 |
| -0,001170186 | 0,119485935 | 0,000839454 | 0,135939163 | 0,000626345 | 0,061022595 | 0,00100166 | 0,003138342 | 1 | 1 | 1 | 0,740648805 | Bacteria | Firmicutes | Clostridia | Clostridiales | Lachnospiraceae | Eisenbergiella | Eisenbergiella | 0,001254386 |
| -0,002953913 | 2,45378E-06 | 0,003795155 | 8,99212E-15 | -0,000755246 | 0,004786473 | -0,001031086 | 0,000145473 | 0,000480942 | 2,23904E-12 | 1 | 0,035640849 | Bacteria | Firmicutes | Bacilli | Lactobacillales | Enterococcaceae | Melissococcus | Melissococcus | 0,001232018 |
| 0,003438482 | 0,014802288 | -0,001647072 | 0,123555993 | -0,000283546 | 0,687953966 | 0,000354136 | 0,617247073 | 1 | 1 | 1 | 1 | Bacteria | Actinobacteria | Coriobacteriia | Coriobacteriales | Atopobiaceae | Olsenella | Olsenella | 0,00120614 |
| -0,002181265 | 0,532285714 | 0,000567809 | 0,827730115 | 0,002445429 | 0,108358983 | 0,001316288 | 0,389102725 | 1 | 1 | 1 | 1 | Bacteria | Proteobacteria | Gammaproteobacteria | Enterobacteriales | Enterobacteriaceae | Yersinia | Yersinia | 0,001134211 |
| -0,000228399 | 0,661038858 | 0,00057371 | 0,150146576 | -0,000223328 | 0,399179805 | 0,000756416 | 0,004917372 | 1 | 1 | 1 | 1 | Bacteria | Firmicutes | Erysipelotrichia | Erysipelotrichales | Erysipelotrichaceae | Erysipelatoclostridium | Erysipelatoclostridium | 0,001107456 |
| 0,003758185 | 0,060929943 | -0,001772685 | 0,235227942 | 0,00029721 | 0,731460107 | -0,000338047 | 0,698155901 | 1 | 1 | 1 | 1 | Bacteria | Proteobacteria | Gammaproteobacteria | Pseudomonadales | Moraxellaceae | Acinetobacter | Acinetobacter | 0,001067544 |
| 0,002993999 | 2,03219E-12 | -0,001328026 | 1,42309E-05 | -2,52589E-05 | 0,900770523 | -7,17677E-06 | 0,971823388 | 4,4505E-10 | 0,003116557 | 1 | 1 | Bacteria | Proteobacteria | Deltaproteobacteria | Desulfovibrionales | Desulfovibrionaceae | Desulfovibrio | Desulfovibrio | 0,001044298 |
| 0,000626561 | 0,336220322 | 0,000802172 | 0,112583851 | -0,000660848 | 0,07735591 | -0,000516116 | 0,167767108 | 1 | 1 | 1 | 1 | Bacteria | Firmicutes | Erysipelotrichia | Erysipelotrichales | Erysipelotrichaceae | Turicibacter | Turicibacter | 0,001033333 |
| -0,001554259 | 0,142027443 | 0,000778465 | 0,324557877 | 0,001163986 | 0,011674849 | 0,000851793 | 0,065821376 | 1 | 1 | 1 | 1 | Bacteria | Firmicutes | Clostridia | Clostridiales | Eubacteriaceae | Eubacterium | Eubacterium | 0,00095307 |
| 0,000402449 | 0,377328751 | 4,9409E-05 | 0,884536944 | -5,33385E-05 | 0,787455648 | 0,000588902 | 0,003577593 | 1 | 1 | 1 | 0,837156718 | Bacteria | Firmicutes | Clostridia | Clostridiales | Lachnospiraceae | Lachnospiraceae_UCG-004 | Lachnospiraceae_UCG-004 | 0,000950439 |
| 0,001900011 | 1,10675E-18 | -0,000742378 | 9,0545E-07 | -0,000263431 | 0,014224089 | 0,000260516 | 0,015488407 | 2,56767E-16 | 0,000203726 | 1 | 1 | Bacteria | Firmicutes | Clostridia | Clostridiales | Ruminococcaceae | Ruminiclostridium_9 | Ruminiclostridium_9 | 0,000911404 |
| 0,001843033 | 5,22452E-08 | -0,000677945 | 0,006872594 | -0,000347918 | 0,050243771 | 0,000257761 | 0,146748279 | 1,05013E-05 | 1 | 1 | 1 | Bacteria | Firmicutes | Clostridia | Clostridiales | Ruminococcaceae | Ruminococcaceae_UCG-004 | Ruminococcaceae_UCG-004 | 0,00087193 |
| -0,000406325 | 0,267120069 | 0,001144482 | 5,00583E-05 | -8,30161E-05 | 0,626882912 | -0,00046688 | 0,007132641 | 1 | 0,010562301 | 1 | 1 | Bacteria | Actinobacteria | Actinobacteria | Actinomycetales | Actinomycetaceae | Actinomyces | Actinomyces | 0,000804825 |
| -0,000317501 | 0,532018025 | 0,000195272 | 0,610120561 | 0,000449602 | 0,057322387 | 0,00067938 | 0,004536781 | 1 | 1 | 1 | 1 | Bacteria | Proteobacteria | Gammaproteobacteria | Betaproteobacteriales | Burkholderiaceae | Parasutterella | Parasutterella | 0,000797368 |
| 0,001741386 | 1,18579E-07 | -0,000652122 | 0,006949585 | -0,000117208 | 0,454793941 | 3,77639E-05 | 0,810257453 | 2,37157E-05 | 1 | 1 | 1 | Bacteria | Firmicutes | Clostridia | Clostridiales | Ruminococcaceae | Ruminococcaceae_UCG-010 | Ruminococcaceae_UCG-010 | 0,000779386 |
| 0,002091612 | 0,038671946 | -0,000994172 | 0,195009906 | -0,000111801 | 0,823772518 | 0,000172755 | 0,731770333 | 1 | 1 | 1 | 1 | Bacteria | Firmicutes | Clostridia | Clostridiales | Ruminococcaceae | CAG-352 | CAG-352 | 0,000770614 |
| 0,001831497 | 0,186291049 | -0,000337827 | 0,743871867 | -0,000367059 | 0,541018266 | -0,000433071 | 0,47372747 | 1 | 1 | 1 | 1 | Bacteria | Firmicutes | Clostridia | Clostridiales | Ruminococcaceae | DTU089 | DTU089 | 0,000765789 |
| -0,001687639 | 0,036570632 | 0,001872572 | 0,002065965 | -9,29749E-05 | 0,791604879 | -0,00025279 | 0,475565031 | 1 | 0,394599395 | 1 | 1 | Bacteria | Actinobacteria | Actinobacteria | Bifidobacteriales | Bifidobacteriaceae | Alloscardovia | Alloscardovia | 0,000754825 |
| -0,000649945 | 0,294572299 | 0,000887152 | 0,058100414 | 0,000532335 | 0,061355783 | -0,000217281 | 0,445457272 | 1 | 1 | 1 | 1 | Bacteria | Firmicutes | Bacilli | Lactobacillales | Streptococcaceae | Lactococcus | Lactococcus | 0,000744737 |
| -8,45233E-05 | 0,796636288 | 0,000359926 | 0,147425919 | -0,000370967 | 0,017038392 | 0,000597413 | 0,000165397 | 1 | 1 | 1 | 0,040356841 | Bacteria | Firmicutes | Clostridia | Clostridiales | Ruminococcaceae | Flavonifractor | Flavonifractor | 0,000736404 |
| 0,001341417 | 0,050047536 | -0,000499682 | 0,327649 | -4,56953E-05 | 0,881425922 | 0,000157577 | 0,60920406 | 1 | 1 | 1 | 1 | Bacteria | Firmicutes | Clostridia | Clostridiales | Peptostreptococcaceae | Peptostreptococcus | Peptostreptococcus | 0,000725439 |
| 0,000734275 | 0,11406084 | -7,38375E-06 | 0,982524599 | -0,000715715 | 0,033340928 | 0,000548922 | 0,103569523 | 1 | 1 | 1 | 1 | Bacteria | Actinobacteria | Coriobacteriia | Coriobacteriales | Eggerthellaceae | Eggerthella | Eggerthella | 0,000703509 |
| 0,002323144 | 0,069980613 | -0,000714733 | 0,454406611 | -0,00013981 | 0,800896044 | -0,000607955 | 0,276524541 | 1 | 1 | 1 | 1 | Bacteria | Bacteroidetes | Bacteroidia | Bacteroidales | Muribaculaceae | CAG-873 | CAG-873 | 0,000688158 |
| 0,000955842 | 0,214089774 | -0,000234607 | 0,688665186 | -0,000430175 | 0,267780053 | 0,00030074 | 0,439712334 | 1 | 1 | 1 | 1 | Bacteria | Firmicutes | Clostridia | Clostridiales | Lachnospiraceae | Butyrivibrio | Butyrivibrio | 0,000664035 |
| 0,002313058 | 0,040819593 | -0,001107894 | 0,188110014 | 6,35462E-05 | 0,89663408 | -0,000102489 | 0,835082752 | 1 | 1 | 1 | 1 | Bacteria | Bacteroidetes | Bacteroidia | Bacteroidales | Prevotellaceae | Prevotellaceae_UCG-001 | Prevotellaceae_UCG-001 | 0,000657018 |
| 0,000895461 | 0,002509819 | -0,000234003 | 0,293309566 | -0,000213859 | 0,144516639 | 0,000224472 | 0,12713759 | 0,426669218 | 1 | 1 | 1 | Bacteria | Firmicutes | Clostridia | Clostridiales | Lachnospiraceae | Lachnospiraceae_UCG-010 | Lachnospiraceae_UCG-010 | 0,000635088 |
| 0,000647924 | 0,021596697 | 1,99759E-05 | 0,924552461 | -0,000451934 | 0,000970917 | 0,000245663 | 0,069239971 | 1 | 1 | 0,238845653 | 1 | Bacteria | Proteobacteria | Gammaproteobacteria | Betaproteobacteriales | Burkholderiaceae | Sutterella | Sutterella | 0,000632018 |
| -0,001733681 | 0,178683441 | 0,002723202 | 0,005003686 | -0,000759254 | 0,174766568 | -0,001355878 | 0,016520135 | 1 | 0,93068567 | 1 | 1 | Bacteria | Proteobacteria | Gammaproteobacteria | Enterobacteriales | Enterobacteriaceae | Cronobacter | Cronobacter | 0,000619298 |
| 4,63687E-05 | 0,913241978 | 7,63752E-06 | 0,980933997 | 0,000614339 | 0,001579326 | 0,000250196 | 0,194789499 | 1 | 1 | 0,385355641 | 1 | Bacteria | Bacteroidetes | Bacteroidia | Bacteroidales | Marinifilaceae | Butyricimonas | Butyricimonas | 0,000614035 |
| 0,001435832 | 3,40999E-07 | -0,000602763 | 0,00322331 | -7,5285E-05 | 0,529661547 | 8,47691E-05 | 0,481836242 | 6,78588E-05 | 0,605982219 | 1 | 1 | Bacteria | Actinobacteria | Coriobacteriia | Coriobacteriales | Eggerthellaceae | Adlercreutzia | Adlercreutzia | 0,000596053 |
| 0,000738244 | 0,005017591 | -0,000188473 | 0,334131977 | -0,000123599 | 0,27544089 | 0,000229458 | 0,044938374 | 0,80783211 | 1 | 1 | 1 | Bacteria | Firmicutes | Clostridia | Clostridiales | Ruminococcaceae | Oscillibacter | Oscillibacter | 0,00059386 |
| 0,001522324 | 0,001960555 | -0,000583474 | 0,118464587 | 1,09726E-05 | 0,974595576 | 3,8909E-05 | 0,910286186 | 0,341136633 | 1 | 1 | 1 | Bacteria | Firmicutes | Negativicutes | Selenomonadales | Veillonellaceae | Megasphaera | Megasphaera | 0,000578509 |
| -0,001050387 | 0,074755838 | 0,001329376 | 0,002722186 | 0,000590282 | 0,02133608 | -0,000735571 | 0,004495061 | 1 | 0,514493196 | 1 | 1 | Bacteria | Actinobacteria | Actinobacteria | Micrococcales | Micrococcaceae | Rothia | Rothia | 0,000570175 |
| 0,001756707 | 5,27765E-09 | -0,000748776 | 0,000637707 | -0,000286819 | 0,023225695 | 5,71059E-05 | 0,651649426 | 1,07664E-06 | 0,12754139 | 1 | 1 | Bacteria | Firmicutes | Clostridia | Clostridiales | Lachnospiraceae | Lachnospiraceae_UCG-001 | Lachnospiraceae_UCG-001 | 0,000562281 |
| -0,000515031 | 0,492763845 | 0,000956979 | 0,089049952 | 0,000194119 | 0,551316179 | -0,000534331 | 0,104121688 | 1 | 1 | 1 | 1 | Bacteria | Proteobacteria | Gammaproteobacteria | Enterobacteriales | Enterobacteriaceae | Raoultella | Raoultella | 0,00051886 |
| 0,001431228 | 3,58038E-10 | -0,000626964 | 0,000131455 | -0,000151411 | 0,117097287 | 8,33855E-05 | 0,389524552 | 7,66202E-08 | 0,027474196 | 1 | 1 | Bacteria | Firmicutes | Clostridia | Clostridiales | Ruminococcaceae | Ruminococcaceae_UCG-003 | Ruminococcaceae_UCG-003 | 0,000514912 |
| -0,000441925 | 0,451575654 | 0,00029664 | 0,498904433 | 0,000212446 | 0,405042197 | 0,000457537 | 0,075903697 | 1 | 1 | 1 | 1 | Bacteria | Firmicutes | Erysipelotrichia | Erysipelotrichales | Erysipelotrichaceae | Faecalitalea | Faecalitalea | 0,000499123 |
| 0,001159966 | 0,024182303 | -0,000429712 | 0,264738023 | -0,000134128 | 0,570076775 | 3,06147E-05 | 0,897339861 | 1 | 1 | 1 | 1 | Bacteria | Bacteroidetes | Bacteroidia | Bacteroidales | Prevotellaceae | Prevotellaceae_NK3B31_group | Prevotellaceae_NK3B31_group | 0,00049386 |
| 0,000871938 | 0,005346836 | -0,000272517 | 0,244460042 | -0,000259477 | 0,079413646 | 0,000190365 | 0,19907756 | 0,855493782 | 1 | 1 | 1 | Bacteria | Firmicutes | Clostridia | Clostridiales | Peptococcaceae | Peptococcus | Peptococcus | 0,000491228 |
| 0,000890591 | 0,262860542 | -0,000631563 | 0,288065703 | 0,00025226 | 0,464557635 | 0,000429714 | 0,216307429 | 1 | 1 | 1 | 1 | Bacteria | Firmicutes | Negativicutes | Selenomonadales | Veillonellaceae | Megamonas | Megamonas | 0,000486404 |
| -0,001373492 | 0,053480376 | 0,001711617 | 0,001393849 | 0,000586873 | 0,057240967 | -0,001107195 | 0,000417375 | 1 | 0,271800638 | 1 | 0,101004773 | Bacteria | Firmicutes | Bacilli | Bacillales | Paenibacillaceae | Paenibacillus | Paenibacillus | 0,000478947 |
| -1,28361E-05 | 0,979746858 | -6,10191E-05 | 0,871852448 | 0,00069437 | 0,001985049 | 0,000224208 | 0,314432744 | 1 | 1 | 0,48236694 | 1 | Bacteria | Firmicutes | Erysipelotrichia | Erysipelotrichales | Erysipelotrichaceae | Merdibacter | Merdibacter | 0,000478509 |
| 0,001432627 | 4,18039E-09 | -0,000651329 | 0,000250787 | 2,91898E-05 | 0,774193242 | -7,14676E-05 | 0,485449919 | 8,6116E-07 | 0,051912932 | 1 | 1 | Bacteria | Firmicutes | Clostridia | Clostridiales | Ruminococcaceae | Negativibacillus | Negativibacillus | 0,000444737 |
| 0,001177961 | 3,80138E-14 | -0,000591118 | 1,05796E-07 | 9,77264E-05 | 0,147205066 | 5,81465E-05 | 0,389601804 | 8,47707E-12 | 2,42272E-05 | 1 | 1 | Bacteria | Firmicutes | Clostridia | Clostridiales | Lachnospiraceae | Lachnospiraceae_UCG-008 | Lachnospiraceae_UCG-008 | 0,000430263 |
| -0,000413128 | 0,427662429 | 0,000344451 | 0,383338407 | -0,000170282 | 0,499724284 | 0,000503566 | 0,048022694 | 1 | 1 | 1 | 1 | Bacteria | Firmicutes | Clostridia | Clostridiales | Lachnospiraceae | UC5-1-2E3 | UC5-1-2E3 | 0,000428509 |
| 0,001356642 | 0,052111924 | -0,000628154 | 0,22789899 | -0,000117007 | 0,702134808 | 4,23821E-05 | 0,890474018 | 1 | 1 | 1 | 1 | Bacteria | Firmicutes | Clostridia | Clostridiales | Family_XI | Parvimonas | Parvimonas | 0,000419737 |
| 0,001112374 | 2,67129E-12 | -0,000472051 | 3,81079E-05 | -1,82703E-05 | 0,77960581 | -1,69585E-05 | 0,796357501 | 5,82341E-10 | 0,008155092 | 1 | 1 | Bacteria | Proteobacteria | Deltaproteobacteria | Desulfovibrionales | Desulfovibrionaceae | Bilophila | Bilophila | 0,000404386 |
| -8,55433E-05 | 0,738024128 | 5,11912E-05 | 0,791105441 | 0,00036928 | 0,002508647 | 0,000233722 | 0,054880102 | 1 | 1 | 0,607092681 | 1 | Bacteria | Bacteroidetes | Bacteroidia | Bacteroidales | Barnesiellaceae | Coprobacter | Coprobacter | 0,00039693 |
| -0,000631162 | 0,060354745 | 0,001058797 | 3,31723E-05 | -0,000429672 | 0,003394638 | -0,000311375 | 0,034093077 | 1 | 0,007132041 | 0,814713209 | 1 | Bacteria | Firmicutes | Bacilli | Lactobacillales | Carnobacteriaceae | Granulicatella | Granulicatella | 0,000394298 |
| -9,3191E-07 | 0,998927486 | 0,000221629 | 0,668954275 | 0,000396796 | 0,188085527 | -0,000191419 | 0,527507233 | 1 | 1 | 1 | 1 | Bacteria | Proteobacteria | Gammaproteobacteria | Enterobacteriales | Enterobacteriaceae | Hafnia-Obesumbacterium | Hafnia-Obesumbacterium | 0,000376316 |
| 0,000175947 | 0,367950453 | 3,84753E-05 | 0,792085699 | -2,81727E-06 | 0,973476933 | 0,000120638 | 0,15814386 | 1 | 1 | 1 | 1 | Bacteria | Firmicutes | Clostridia | Clostridiales | Peptostreptococcaceae | Asaccharospora | Asaccharospora | 0,000333772 |
| 0,000469107 | 0,004403027 | -0,000155349 | 0,207117816 | -0,000137314 | 0,075383384 | 0,000189881 | 0,014830884 | 0,717693404 | 1 | 1 | 1 | Bacteria | Proteobacteria | Gammaproteobacteria | Enterobacteriales | Enterobacteriaceae | Pseudocitrobacter | Pseudocitrobacter | 0,000331579 |
| -7,99699E-06 | 0,986663359 | -0,00012833 | 0,729263645 | 0,000417052 | 0,131779028 | 0,000333016 | 0,229024043 | 1 | 1 | 1 | 1 | Bacteria | Actinobacteria | Coriobacteriia | Coriobacteriales | Coriobacteriaceae | Enorma | Enorma | 0,00033114 |
| 0,000127057 | 0,470073843 | 1,08126E-05 | 0,937146036 | -0,000168597 | 0,143144793 | 0,000345921 | 0,002934625 | 1 | 1 | 1 | 0,695506231 | Bacteria | Firmicutes | Clostridia | Clostridiales | Lachnospiraceae | GCA-900066575 | GCA-900066575 | 0,000325 |
| 0,000567246 | 0,001712639 | -0,000166441 | 0,219093805 | -1,63597E-05 | 0,853038324 | 9,84383E-06 | 0,911585294 | 0,299711874 | 1 | 1 | 1 | Bacteria | Actinobacteria | Actinobacteria | Bifidobacteriales | Bifidobacteriaceae | Pseudoscardovia | Pseudoscardovia | 0,000320175 |
| 0,00096867 | 0,000945934 | -0,000459046 | 0,035893753 | -8,37112E-06 | 0,951248462 | 2,0087E-05 | 0,88390268 | 0,169322158 | 1 | 1 | 1 | Bacteria | Actinobacteria | Coriobacteriia | Coriobacteriales | Atopobiaceae | Libanicoccus | Libanicoccus | 0,000310965 |
| 0,000903705 | 0,001076438 | -0,000508864 | 0,013162978 | 6,05333E-05 | 0,613905046 | 0,000121271 | 0,31568564 | 0,19160599 | 1 | 1 | 1 | Bacteria | Bacteroidetes | Bacteroidia | Bacteroidales | Rikenellaceae | Rikenellaceae_RC9_gut_group | Rikenellaceae_RC9_gut_group | 0,00030614 |
| 0,000824476 | 2,88769E-20 | -0,000366201 | 5,95263E-09 | -7,00311E-05 | 0,047420492 | 4,82652E-05 | 0,173616303 | 6,78608E-18 | 1,38696E-06 | 1 | 1 | Bacteria | Firmicutes | Clostridia | Clostridiales | Family_XIII | Family_XIII_UCG-001 | Family_XIII_UCG-001 | 0,00029693 |
| -0,000440192 | 0,373398911 | 0,000391168 | 0,290713969 | -0,00026983 | 0,214236264 | 0,000338554 | 0,121943128 | 1 | 1 | 1 | 1 | Bacteria | Tenericutes | Mollicutes | Anaeroplasmatales | Anaeroplasmataceae | Anaeroplasma | Anaeroplasma | 0,000267105 |
| 0,000700448 | 0,127398177 | -0,00029081 | 0,396304339 | 0,000124262 | 0,532291623 | -0,000101613 | 0,611836467 | 1 | 1 | 1 | 1 | Bacteria | Fusobacteria | Fusobacteriia | Fusobacteriales | Fusobacteriaceae | Fusobacterium | Fusobacterium | 0,000261404 |
| 0,0003537 | 0,374694636 | -3,54246E-05 | 0,90532433 | 2,84153E-05 | 0,870701781 | -6,74975E-05 | 0,700862722 | 1 | 1 | 1 | 1 | Bacteria | Firmicutes | Clostridia | Clostridiales | Ruminococcaceae | Anaerotruncus | Anaerotruncus | 0,000260965 |
| 0,00073462 | 0,000169436 | -0,000283705 | 0,050922734 | -8,1794E-05 | 0,372732154 | -2,62565E-05 | 0,775507987 | 0,03219289 | 1 | 1 | 1 | Bacteria | Firmicutes | Clostridia | Clostridiales | Family_XIII | Mogibacterium | Mogibacterium | 0,000257018 |
| -0,000639428 | 0,039617958 | 0,000823918 | 0,000440055 | -0,00010755 | 0,423293923 | -0,000279307 | 0,039617939 | 1 | 0,090211237 | 1 | 1 | Bacteria | Firmicutes | Bacilli | Lactobacillales | Carnobacteriaceae | Isobaculum | Isobaculum | 0,00025307 |
| 0,000580852 | 0,004103429 | -0,00011554 | 0,446121108 | 1,01431E-05 | 0,943855613 | -0,000115107 | 0,425884311 | 0,677065709 | 1 | 1 | 1 | Bacteria | Firmicutes | Negativicutes | Selenomonadales | Veillonellaceae | Mitsuokella | Mitsuokella | 0,00025307 |
| 0,000142464 | 0,552597011 | 0,000137363 | 0,449544164 | -9,55171E-05 | 0,404531082 | -4,99677E-05 | 0,664013948 | 1 | 1 | 1 | 1 | Bacteria | Firmicutes | Bacilli | Bacillales | Family_XI | Gemella | Gemella | 0,000250439 |
| 0,000847035 | 0,02852037 | -0,000330547 | 0,243461041 | -0,000154085 | 0,361157453 | -4,00347E-05 | 0,81268684 | 1 | 1 | 1 | 1 | Bacteria | Actinobacteria | Coriobacteriia | Coriobacteriales | Atopobiaceae | Coriobacteriaceae_UCG-003 | Coriobacteriaceae_UCG-003 | 0,000246053 |
| 0,000369052 | 0,035611872 | -0,000207139 | 0,128339639 | 0,000178796 | 0,091136608 | 7,62256E-05 | 0,470523916 | 1 | 1 | 1 | 1 | Bacteria | Firmicutes | Erysipelotrichia | Erysipelotrichales | Erysipelotrichaceae | Solobacterium | Solobacterium | 0,000244737 |
| 0,000802696 | 0,002125729 | -0,000448658 | 0,020065274 | 3,28543E-05 | 0,768968489 | 7,62083E-05 | 0,498721567 | 0,367751125 | 1 | 1 | 1 | Bacteria | Firmicutes | Clostridia | Clostridiales | Lachnospiraceae | Tyzzerella_3 | Tyzzerella_3 | 0,000236842 |
| 0,000508229 | 0,001172795 | -0,000224305 | 0,05582256 | -3,97189E-06 | 0,957624269 | 6,33433E-05 | 0,399341033 | 0,207584767 | 1 | 1 | 1 | Bacteria | Firmicutes | Clostridia | Clostridiales | Ruminococcaceae | Fournierella | Fournierella | 0,000236842 |
| 0,000760593 | 0,253708591 | -0,000195083 | 0,694947035 | -0,000132394 | 0,646693084 | -0,000205734 | 0,479297077 | 1 | 1 | 1 | 1 | Bacteria | Proteobacteria | Gammaproteobacteria | Aeromonadales | Succinivibrionaceae | Succinivibrio | Succinivibrio | 0,000227632 |
| 0,000714201 | 0,28509689 | -0,000313326 | 0,530073026 | 0,000247808 | 0,39253997 | -0,000213326 | 0,464507355 | 1 | 1 | 1 | 1 | Bacteria | Actinobacteria | Actinobacteria | Micrococcales | Micrococcaceae | Glutamicibacter | Glutamicibacter | 0,000215351 |
| 0,00024751 | 0,537779135 | 9,95826E-05 | 0,740072833 | 6,27732E-05 | 0,718702704 | -0,000259524 | 0,139909617 | 1 | 1 | 1 | 1 | Bacteria | Proteobacteria | Gammaproteobacteria | Enterobacteriales | Enterobacteriaceae | Proteus | Proteus | 0,000203509 |
| 0,000301583 | 2,19007E-05 | -9,77857E-05 | 0,061394407 | -8,50178E-05 | 0,008601009 | 0,000104888 | 0,001334871 | 0,00420493 | 1 | 1 | 0,319034192 | Bacteria | Firmicutes | Clostridia | Clostridiales | Ruminococcaceae | GCA-900066225 | GCA-900066225 | 0,00020307 |
| 0,000469816 | 0,073148692 | -0,0001905 | 0,306034142 | -0,000129435 | 0,48978398 | 0,000157742 | 0,40285571 | 1 | 1 | 1 | 1 | Bacteria | Actinobacteria | Actinobacteria | Corynebacteriales | Corynebacteriaceae | Corynebacterium_1 | Corynebacterium_1 | 0,000200439 |
| 0,000695135 | 0,118133501 | -0,000446048 | 0,179466866 | 0,000151914 | 0,430399195 | 7,4551E-05 | 0,700492324 | 1 | 1 | 1 | 1 | Bacteria | Bacteroidetes | Bacteroidia | Bacteroidales | Prevotellaceae | Prevotella | Prevotella | 0,000198246 |
| 0,000531823 | 5,58762E-09 | -0,00024954 | 0,000206429 | -2,96883E-05 | 0,504583646 | 4,87224E-05 | 0,275547313 | 1,13429E-06 | 0,042937213 | 1 | 1 | Bacteria | Firmicutes | Clostridia | Clostridiales | Lachnospiraceae | Howardella | Howardella | 0,000197807 |
| 0,000603764 | 0,000170771 | -0,000257512 | 0,030189524 | 1,03214E-05 | 0,880427233 | -4,73978E-05 | 0,492725671 | 0,032275789 | 1 | 1 | 1 | Bacteria | Firmicutes | Clostridia | Clostridiales | Ruminococcaceae | Ruminococcaceae_UCG-008 | Ruminococcaceae_UCG-008 | 0,000196053 |
| -0,000548228 | 0,347532036 | 0,000854025 | 0,051112688 | -0,00022859 | 0,367186553 | -0,000427113 | 0,095119893 | 1 | 1 | 1 | 1 | Bacteria | Proteobacteria | Gammaproteobacteria | Enterobacteriales | Enterobacteriaceae | Buttiauxella | Buttiauxella | 0,000193421 |
| 9,00971E-05 | 0,679241356 | -2,58859E-05 | 0,873925243 | 0,000136261 | 0,157114494 | 7,33482E-05 | 0,448054571 | 1 | 1 | 1 | 1 | Bacteria | Firmicutes | Clostridia | Clostridiales | Lachnospiraceae | Shuttleworthia | Shuttleworthia | 0,000191667 |
| 0,000432822 | 6,47301E-07 | -0,000202138 | 0,00200255 | -3,36837E-05 | 0,468965283 | 6,97009E-05 | 0,135730539 | 0,000127518 | 0,384489505 | 1 | 1 | Bacteria | Firmicutes | Clostridia | Clostridiales | Ruminococcaceae | Anaerofilum | Anaerofilum | 0,000184211 |
| 0,000621664 | 0,043598316 | -0,000299561 | 0,191579869 | 2,48091E-05 | 0,859598806 | -2,17779E-05 | 0,877221289 | 1 | 1 | 1 | 1 | Bacteria | Synergistetes | Synergistia | Synergistales | Synergistaceae | Cloacibacillus | Cloacibacillus | 0,000179825 |
| 0,000417268 | 1,68098E-05 | -0,000163786 | 0,013301075 | 1,44696E-05 | 0,72937191 | -9,25464E-06 | 0,825441478 | 0,003244283 | 1 | 1 | 1 | Bacteria | Firmicutes | Clostridia | Clostridiales | Ruminococcaceae | Caproiciproducens | Caproiciproducens | 0,000176316 |
| 8,22812E-06 | 0,947282298 | 3,38242E-05 | 0,716176813 | 6,98563E-05 | 0,196670587 | 8,87258E-05 | 0,103642241 | 1 | 1 | 1 | 1 | Bacteria | Firmicutes | Clostridia | Clostridiales | Ruminococcaceae | Pygmaiobacter | Pygmaiobacter | 0,000172368 |
| -0,000472962 | 0,345014003 | 0,000739585 | 0,049058774 | -0,00021071 | 0,332831389 | -0,000363662 | 0,097710783 | 1 | 1 | 1 | 1 | Bacteria | Proteobacteria | Gammaproteobacteria | Enterobacteriales | Enterobacteriaceae | Pluralibacter | Pluralibacter | 0,000167544 |
| 0,000555935 | 0,285552442 | -0,000244412 | 0,529589919 | 0,000193798 | 0,390776828 | -0,000166376 | 0,464063401 | 1 | 1 | 1 | 1 | Bacteria | Bacteroidetes | Bacteroidia | Bacteroidales | Dysgonomonadaceae | Dysgonomonas | Dysgonomonas | 0,000167105 |
| 0,000437046 | 0,125076179 | -0,000150463 | 0,479009868 | -3,56771E-05 | 0,772394491 | -4,47074E-05 | 0,718777979 | 1 | 1 | 1 | 1 | Bacteria | Proteobacteria | Gammaproteobacteria | Pseudomonadales | Pseudomonadaceae | Pseudomonas | Pseudomonas | 0,000160965 |
| 0,000509902 | 6,03971E-10 | -0,000247805 | 3,88029E-05 | -4,63789E-06 | 0,904076697 | 1,00431E-05 | 0,794905285 | 1,28042E-07 | 0,008265013 | 1 | 1 | Archaea | Euryarchaeota | Methanobacteria | Methanobacteriales | Methanobacteriaceae | Methanobrevibacter | Methanobrevibacter | 0,000160088 |
| 0,000421654 | 0,000602442 | -0,0001843 | 0,0430374 | 5,057E-06 | 0,923469958 | 7,12008E-07 | 0,989278291 | 0,109644509 | 1 | 1 | 1 | Bacteria | Actinobacteria | Coriobacteriia | Coriobacteriales | Eggerthellaceae | Gordonibacter | Gordonibacter | 0,000158333 |
| 0,000379715 | 0,000795406 | -0,00016307 | 0,051778233 | -5,59046E-05 | 0,263185703 | 4,97302E-05 | 0,322147565 | 0,143968529 | 1 | 1 | 1 | Bacteria | Actinobacteria | Coriobacteriia | Coriobacteriales | Eggerthellaceae | DNF00809 | DNF00809 | 0,000155702 |
| 4,18254E-05 | 0,78499037 | -7,83014E-05 | 0,49633631 | 0,00022148 | 0,001480968 | 0,000117199 | 0,090749038 | 1 | 1 | 0,362837261 | 1 | Bacteria | Firmicutes | Erysipelotrichia | Erysipelotrichales | Erysipelotrichaceae | Dielma | Dielma | 0,00015307 |
| -0,00041567 | 0,357614013 | 0,000657667 | 0,052372888 | -0,000182973 | 0,351206704 | -0,000334837 | 0,091056701 | 1 | 1 | 1 | 1 | Bacteria | Firmicutes | Clostridia | Clostridiales | Lachnospiraceae | Lachnoclostridium_5 | Lachnoclostridium_5 | 0,000146491 |
| 0,000454059 | 0,002370687 | -0,000198803 | 0,071892996 | -3,35504E-05 | 0,607018583 | -1,13979E-05 | 0,862036858 | 0,405387524 | 1 | 1 | 1 | Bacteria | Firmicutes | Negativicutes | Selenomonadales | Acidaminococcaceae | Acidaminococcus | Acidaminococcus | 0,000137281 |
| 0,000386961 | 0,04284386 | -0,000118359 | 0,405475195 | -3,29272E-05 | 0,690005201 | -7,39122E-05 | 0,374067151 | 1 | 1 | 1 | 1 | Bacteria | Firmicutes | Clostridia | Clostridiales | Family_XI | Peptoniphilus | Peptoniphilus | 0,000133772 |
| 0,000309538 | 5,02611E-09 | -0,000126694 | 0,00095618 | -3,3053E-05 | 0,159338216 | 2,32149E-05 | 0,324564478 | 1,03035E-06 | 0,189323717 | 1 | 1 | Bacteria | Firmicutes | Clostridia | Clostridiales | Defluviitaleaceae | Defluviitaleaceae_UCG-011 | Defluviitaleaceae_UCG-011 | 0,000128947 |
| 0,000373023 | 0,213653605 | -0,000100184 | 0,654520174 | -5,06557E-05 | 0,696730168 | -9,21189E-05 | 0,481498411 | 1 | 1 | 1 | 1 | Bacteria | Firmicutes | Clostridia | Clostridiales | Family_XI | Finegoldia | Finegoldia | 0,000120614 |
| 7,41283E-05 | 0,702819785 | -9,64766E-05 | 0,511786434 | 0,000163377 | 0,078095372 | 0,000102904 | 0,268002494 | 1 | 1 | 1 | 1 | Bacteria | Firmicutes | Erysipelotrichia | Erysipelotrichales | Erysipelotrichaceae | Faecalicoccus | Faecalicoccus | 0,000114912 |
| 0,000377919 | 0,285552442 | -0,000166149 | 0,529589919 | 0,000131742 | 0,390776828 | -0,000113101 | 0,464063401 | 1 | 1 | 1 | 1 | Bacteria | Firmicutes | Negativicutes | Selenomonadales | Veillonellaceae | Schwartzia | Schwartzia | 0,000113596 |
| 0,000204825 | 0,083152433 | -2,3158E-05 | 0,577211855 | -5,78938E-06 | 0,900750865 | -8,23021E-06 | 0,861639574 | 1 | 1 | 1 | 1 | Bacteria | Firmicutes | Clostridia | Clostridiales | Lachnospiraceae | Tyzzerella_4 | Tyzzerella_4 | 0,000107456 |
| 0,000293129 | 0,025669883 | -9,16583E-05 | 0,348430533 | -1,35591E-06 | 0,980921127 | -6,43566E-05 | 0,260116949 | 1 | 1 | 1 | 1 | Bacteria | Firmicutes | Clostridia | Clostridiales | Family_XI | Anaerococcus | Anaerococcus | 0,000104386 |
| 0,000275653 | 0,000245605 | -0,000139674 | 0,011956914 | 5,24491E-06 | 0,871429985 | 2,98867E-05 | 0,359999628 | 0,046173743 | 1 | 1 | 1 | Bacteria | Firmicutes | Clostridia | Clostridiales | Ruminococcaceae | Oscillospira | Oscillospira | 0,000102193 |
| 8,56156E-05 | 0,698676677 | 0,000131722 | 0,425836505 | -8,76013E-05 | 0,361778601 | -0,000146752 | 0,129670273 | 1 | 1 | 1 | 1 | Bacteria | Epsilonbacteraeota | Campylobacteria | Campylobacterales | Campylobacteraceae | Campylobacter | Campylobacter | 0,000101316 |
| 1,04361E-05 | 0,942491684 | 0,000151198 | 0,17531347 | -6,83037E-05 | 0,382352075 | -0,000116947 | 0,136488027 | 1 | 1 | 1 | 1 | Bacteria | Firmicutes | Clostridia | Clostridiales | Lachnospiraceae | Lachnospiraceae_UCG-003 | Lachnospiraceae_UCG-003 | 9,73684E-05 |
| 8,63944E-05 | 0,113100387 | -6,35957E-05 | 0,118661212 | 0,000122554 | 4,50423E-07 | 3,94099E-05 | 0,098087633 | 1 | 1 | 0,000112155 | 1 | Bacteria | Firmicutes | Erysipelotrichia | Erysipelotrichales | Erysipelotrichaceae | Holdemania | Holdemania | 9,64912E-05 |
| -0,000115128 | 0,4883587 | 8,52492E-05 | 0,492444745 | 7,85785E-06 | 0,913175117 | 9,49945E-05 | 0,191171877 | 1 | 1 | 1 | 1 | Bacteria | Firmicutes | Clostridia | Clostridiales | Lachnospiraceae | Robinsoniella | Robinsoniella | 9,42982E-05 |
| -0,000248711 | 0,258867217 | 0,000278489 | 0,091344255 | 0,000186925 | 0,051198492 | -0,000200177 | 0,038137205 | 1 | 1 | 1 | 1 | Bacteria | Proteobacteria | Gammaproteobacteria | Enterobacteriales | Enterobacteriaceae | Salmonella | Salmonella | 8,64035E-05 |
| 2,84181E-08 | 0,999723487 | 2,25573E-05 | 0,713915631 | 2,50788E-05 | 0,493743193 | 4,52401E-05 | 0,220456696 | 1 | 1 | 1 | 1 | Bacteria | Firmicutes | Clostridia | Clostridiales | Lachnospiraceae | Lactonifactor | Lactonifactor | 8,55263E-05 |
| 0,000216071 | 3,23251E-10 | -9,06986E-05 | 0,000110948 | -1,35337E-05 | 0,347244603 | 9,82443E-06 | 0,496324171 | 6,94989E-08 | 0,023299058 | 1 | 1 | Bacteria | Firmicutes | Clostridia | Clostridiales | Ruminococcaceae | Candidatus_Soleaferrea | Candidatus_Soleaferrea | 8,50877E-05 |
| 0,000142999 | 0,000377707 | -4,74217E-05 | 0,107068236 | -1,44002E-05 | 0,411900981 | 1,55028E-05 | 0,379845773 | 0,0698758 | 1 | 1 | 1 | Bacteria | Firmicutes | Clostridia | Clostridiales | Ruminococcaceae | Ruminococcaceae_UCG-009 | Ruminococcaceae_UCG-009 | 7,9386E-05 |
| 0,000169172 | 0,061415819 | -7,35863E-05 | 0,293487571 | -4,51005E-05 | 0,416655057 | 5,40259E-05 | 0,331239025 | 1 | 1 | 1 | 1 | Bacteria | Firmicutes | Clostridia | Clostridiales | Lachnospiraceae | Anaerosporobacter | Anaerosporobacter | 7,80702E-05 |
| -6,257E-05 | 0,721371226 | 7,69076E-05 | 0,557707291 | -6,76264E-05 | 0,37581181 | 6,56647E-05 | 0,392805127 | 1 | 1 | 1 | 1 | Bacteria | Firmicutes | Clostridia | Clostridiales | Ruminococcaceae | Ruminiclostridium | Ruminiclostridium | 6,75439E-05 |
| 0,000134108 | 0,176890164 | -8,39917E-05 | 0,259292331 | -4,1995E-05 | 0,55722065 | 0,000114684 | 0,111056919 | 1 | 1 | 1 | 1 | Bacteria | Firmicutes | Clostridia | Clostridiales | Peptostreptococcaceae | Paraclostridium | Paraclostridium | 6,53509E-05 |
| -0,000170004 | 0,295270701 | 0,000249381 | 0,040763276 | -6,92933E-05 | 0,326187915 | -0,000103264 | 0,146712424 | 1 | 1 | 1 | 1 | Bacteria | Proteobacteria | Gammaproteobacteria | Enterobacteriales | Enterobacteriaceae | Siccibacter | Siccibacter | 6,35965E-05 |
| -0,000121651 | 0,530705339 | 3,16638E-05 | 0,827093424 | 0,000135773 | 0,108580596 | 7,31872E-05 | 0,388720252 | 1 | 1 | 1 | 1 | Bacteria | Firmicutes | Clostridia | Clostridiales | Lachnospiraceae | Lachnoclostridium_10 | Lachnoclostridium_10 | 6,27193E-05 |
| 0,000211164 | 3,48834E-07 | -8,20241E-05 | 0,006848872 | -3,0541E-05 | 0,081413511 | -1,83604E-05 | 0,296919029 | 6,90691E-05 | 1 | 1 | 1 | Bacteria | Firmicutes | Negativicutes | Selenomonadales | Veillonellaceae | Allisonella | Allisonella | 5,96491E-05 |
| 0,000175522 | 0,075522736 | -4,4492E-05 | 0,54545419 | -2,43691E-05 | 0,568415999 | -4,86824E-05 | 0,258102119 | 1 | 1 | 1 | 1 | Bacteria | Firmicutes | Clostridia | Clostridiales | Family_XIII | S5-A14a | S5-A14a | 5,57018E-05 |
| -0,0001363 | 0,028292501 | 0,000192022 | 4,52575E-05 | -5,68372E-05 | 0,035009507 | -6,61859E-05 | 0,014888757 | 1 | 0,009594586 | 1 | 1 | Bacteria | Firmicutes | Bacilli | Lactobacillales | Aerococcaceae | Abiotrophia | Abiotrophia | 5,30702E-05 |
| 9,97775E-05 | 0,000456775 | -2,86719E-05 | 0,17783851 | 3,28487E-06 | 0,812234615 | -1,49674E-05 | 0,2817991 | 0,083589905 | 1 | 1 | 1 | Bacteria | Firmicutes | Clostridia | Clostridiales | Ruminococcaceae | Hydrogenoanaerobacterium | Hydrogenoanaerobacterium | 4,7807E-05 |
| 0,000149303 | 0,229084406 | -4,03036E-05 | 0,663595091 | -2,08902E-05 | 0,697775276 | -3,9224E-05 | 0,468997736 | 1 | 1 | 1 | 1 | Bacteria | Firmicutes | Negativicutes | Selenomonadales | Acidaminococcaceae | Succiniclasticum | Succiniclasticum | 4,5614E-05 |
| 0,000152671 | 0,278556145 | -3,90245E-05 | 0,710662755 | -2,9429E-05 | 0,629952767 | -4,02727E-05 | 0,512524976 | 1 | 1 | 1 | 1 | Bacteria | Firmicutes | Erysipelotrichia | Erysipelotrichales | Erysipelotrichaceae | Asteroleplasma | Asteroleplasma | 4,51754E-05 |
| 2,89779E-05 | 0,592679595 | 3,32124E-05 | 0,34667085 | -4,30118E-05 | 0,244706942 | 5,95609E-06 | 0,872887314 | 1 | 1 | 1 | 1 | Bacteria | Proteobacteria | Gammaproteobacteria | Enterobacteriales | Enterobacteriaceae | Lelliottia | Lelliottia | 4,42982E-05 |
| -0,000124085 | 0,355633559 | 0,000195819 | 0,052014531 | -5,42996E-05 | 0,35207239 | -9,967E-05 | 0,090659063 | 1 | 1 | 1 | 1 | Bacteria | Firmicutes | Clostridia | Clostridiales | Family_XI | Tissierella | Tissierella | 4,34211E-05 |
| 0,000136523 | 0,252114918 | -3,9026E-05 | 0,66101803 | -2,15936E-05 | 0,67600689 | -3,16648E-05 | 0,542699233 | 1 | 1 | 1 | 1 | Bacteria | Firmicutes | Clostridia | Clostridiales | Family_XI | Ezakiella | Ezakiella | 4,07895E-05 |
| -2,59734E-05 | 0,656751874 | 3,59621E-05 | 0,415368361 | -4,55773E-05 | 0,096893283 | 4,46169E-05 | 0,105775274 | 1 | 1 | 1 | 1 | Bacteria | Firmicutes | Erysipelotrichia | Erysipelotrichales | Erysipelotrichaceae | Coprobacillus | Coprobacillus | 3,94737E-05 |
| 5,32122E-05 | 0,004883654 | -7,61026E-06 | 0,590530527 | -1,39263E-05 | 0,142469901 | 2,12817E-06 | 0,822382802 | 0,791151937 | 1 | 1 | 1 | Bacteria | Firmicutes | Clostridia | Clostridiales | Eubacteriaceae | Anaerofustis | Anaerofustis | 3,55263E-05 |
| 8,21762E-05 | 0,165495505 | -2,96443E-05 | 0,50261038 | -2,78457E-05 | 0,278478782 | 1,16471E-05 | 0,65209138 | 1 | 1 | 1 | 1 | Bacteria | Firmicutes | Clostridia | Clostridiales | Peptostreptococcaceae | Paeniclostridium | Paeniclostridium | 3,37719E-05 |
| 8,53831E-05 | 0,010875657 | -3,15285E-05 | 0,215924883 | -3,29088E-06 | 0,889527153 | -2,51354E-06 | 0,915705511 | 1 | 1 | 1 | 1 | Bacteria | Firmicutes | Clostridia | Clostridiales | Lachnospiraceae | Epulopiscium | Epulopiscium | 3,28947E-05 |
| -3,1105E-05 | 0,281056315 | 2,28905E-05 | 0,288557563 | 4,89764E-06 | 0,695400212 | 3,27351E-05 | 0,009847375 | 1 | 1 | 1 | 1 | Bacteria | Firmicutes | Clostridia | Clostridiales | Lachnospiraceae | GCA-900066755 | GCA-900066755 | 3,28947E-05 |
| 8,10719E-05 | 0,015207188 | -2,55828E-05 | 0,321673998 | 9,96066E-06 | 0,645796231 | -1,5933E-05 | 0,462801895 | 1 | 1 | 1 | 1 | Bacteria | Firmicutes | Erysipelotrichia | Erysipelotrichales | Erysipelotrichaceae | Catenisphaera | Catenisphaera | 3,11404E-05 |
| 0,000117856 | 0,218209505 | -8,7991E-05 | 0,218760148 | 3,2408E-05 | 0,434822048 | 2,5765E-05 | 0,537218309 | 1 | 1 | 1 | 1 | Bacteria | Proteobacteria | Gammaproteobacteria | Betaproteobacteriales | Burkholderiaceae | Delftia | Delftia | 3,07018E-05 |
| -5,73439E-05 | 0,345871535 | 7,28216E-05 | 0,110012176 | -3,49703E-05 | 0,186600843 | -5,99566E-07 | 0,982000384 | 1 | 1 | 1 | 1 | Bacteria | Firmicutes | Clostridia | Clostridiales | Clostridiaceae_1 | Clostridium_sensu_stricto_3 | Clostridium_sensu_stricto_3 | 2,80702E-05 |
| 2,75474E-05 | 0,587087931 | 2,88507E-05 | 0,446870733 | -2,27028E-05 | 0,302825178 | -3,4277E-05 | 0,122745074 | 1 | 1 | 1 | 1 | Bacteria | Actinobacteria | Actinobacteria | Corynebacteriales | Corynebacteriaceae | Corynebacterium | Corynebacterium | 2,7193E-05 |
| 9,3676E-05 | 0,112935226 | -6,5882E-05 | 0,13572575 | 1,971E-05 | 0,441199579 | 2,08152E-05 | 0,419067339 | 1 | 1 | 1 | 1 | Bacteria | Firmicutes | Clostridia | Clostridiales | Ruminococcaceae | Harryflintia | Harryflintia | 2,7193E-05 |
| 7,0545E-05 | 0,048150541 | -2,81859E-05 | 0,288363614 | -1,99589E-06 | 0,897542009 | -2,19733E-06 | 0,887968042 | 1 | 1 | 1 | 1 | Bacteria | Firmicutes | Clostridia | Clostridiales | Lachnospiraceae | Tyzzerella | Tyzzerella | 2,67544E-05 |
| 6,22662E-05 | 0,000250704 | -2,99957E-05 | 0,017293887 | 6,04776E-06 | 0,40582242 | 5,27456E-06 | 0,471254234 | 0,046881724 | 1 | 1 | 1 | Bacteria | Firmicutes | Clostridia | Clostridiales | Ruminococcaceae | Ruminococcaceae_UCG-007 | Ruminococcaceae_UCG-007 | 2,67544E-05 |
| -4,68061E-05 | 0,566591335 | 4,44456E-05 | 0,466689171 | -3,86793E-05 | 0,2764941 | 3,57959E-05 | 0,316837225 | 1 | 1 | 1 | 1 | Bacteria | Proteobacteria | Gammaproteobacteria | Enterobacteriales | Enterobacteriaceae | Providencia | Providencia | 2,67544E-05 |
| -7,52033E-05 | 0,355633566 | 0,000118678 | 0,052014536 | -3,29088E-05 | 0,352072398 | -6,04061E-05 | 0,090659073 | 1 | 1 | 1 | 1 | Bacteria | Firmicutes | Clostridia | Clostridiales | Clostridiaceae_2 | Alkaliphilus | Alkaliphilus | 2,63158E-05 |
| 8,79331E-05 | 0,003823943 | -3,72891E-05 | 0,098681248 | 2,75711E-06 | 0,832944763 | -1,11914E-05 | 0,395332022 | 0,634774524 | 1 | 1 | 1 | Bacteria | Firmicutes | Clostridia | Clostridiales | Lachnospiraceae | Lachnospiraceae_NK4B4_group | Lachnospiraceae_NK4B4_group | 2,58772E-05 |
| 2,49522E-06 | 0,960521733 | 4,04286E-05 | 0,283921068 | -3,6479E-06 | 0,867572711 | -3,95258E-05 | 0,073672369 | 1 | 1 | 1 | 1 | Bacteria | Firmicutes | Bacilli | Bacillales | Bacillaceae | Bacillus | Bacillus | 0,000025 |
| 5,90948E-05 | 0,058117372 | -2,53955E-05 | 0,280395452 | -1,71385E-05 | 0,250173205 | 1,31841E-05 | 0,378113458 | 1 | 1 | 1 | 1 | Bacteria | Actinobacteria | Coriobacteriia | Coriobacteriales | Eggerthellaceae | CHKCI002 | CHKCI002 | 2,41228E-05 |
| -6,89363E-05 | 0,355633564 | 0,000108788 | 0,052014535 | -3,01664E-05 | 0,352072396 | -5,53722E-05 | 0,090659071 | 1 | 1 | 1 | 1 | Bacteria | Proteobacteria | Gammaproteobacteria | Pasteurellales | Pasteurellaceae | Cricetibacter | Cricetibacter | 2,41228E-05 |
| 6,33763E-05 | 0,082314704 | -2,73881E-05 | 0,287785877 | 4,87408E-06 | 0,851346959 | 6,95516E-06 | 0,790411154 | 1 | 1 | 1 | 1 | Bacteria | Firmicutes | Bacilli | Lactobacillales | Leuconostocaceae | Weissella | Weissella | 2,36842E-05 |
| 4,21664E-05 | 0,061371664 | -1,28444E-05 | 0,455488164 | -1,56419E-05 | 0,187697987 | 9,21099E-06 | 0,438418431 | 1 | 1 | 1 | 1 | Bacteria | Actinobacteria | Coriobacteriia | Coriobacteriales | Atopobiaceae | Coriobacteriaceae_UCG-002 | Coriobacteriaceae_UCG-002 | 2,32456E-05 |
| 5,37572E-05 | 0,018810667 | -1,59545E-05 | 0,294579276 | -1,73532E-05 | 0,272000365 | 8,76348E-06 | 0,581638139 | 1 | 1 | 1 | 1 | Bacteria | Firmicutes | Clostridia | Clostridiales | Ruminococcaceae | Pseudoflavonifractor | Pseudoflavonifractor | 2,32456E-05 |
| 5,16616E-05 | 0,019030943 | -2,89424E-05 | 0,07803462 | 8,88184E-06 | 0,350417303 | 9,40905E-06 | 0,32575277 | 1 | 1 | 1 | 1 | Bacteria | Firmicutes | Clostridia | Clostridiales | Lachnospiraceae | FD2005 | FD2005 | 2,2807E-05 |
| 6,8316E-05 | 0,023829689 | -3,61651E-05 | 0,108492604 | 9,66208E-06 | 0,459149848 | 1,18038E-06 | 0,928364423 | 1 | 1 | 1 | 1 | Bacteria | Firmicutes | Clostridia | Clostridiales | Lachnospiraceae | Lachnospiraceae_UCG-006 | Lachnospiraceae_UCG-006 | 2,23684E-05 |
| 1,18611E-06 | 0,957149093 | -5,64192E-06 | 0,738373463 | 1,8846E-05 | 0,100048842 | 2,13358E-05 | 0,063721559 | 1 | 1 | 1 | 1 | Bacteria | Bacteroidetes | Bacteroidia | Bacteroidales | Marinifilaceae | Sanguibacteroides | Sanguibacteroides | 2,19298E-05 |
| -6,27152E-05 | 0,345523958 | 9,83725E-05 | 0,048604246 | -2,58346E-05 | 0,370914664 | -5,04291E-05 | 0,083722568 | 1 | 1 | 1 | 1 | Bacteria | Firmicutes | Bacilli | Bacillales | Paenibacillaceae | Fontibacillus | Fontibacillus | 2,19298E-05 |
| 9,74738E-06 | 0,663785114 | 6,89563E-06 | 0,680773584 | -1,70368E-05 | 0,080966293 | 1,30904E-05 | 0,182176247 | 1 | 1 | 1 | 1 | Bacteria | Actinobacteria | Coriobacteriia | Coriobacteriales | Coriobacteriales_Incertae_Sedis | Raoultibacter | Raoultibacter | 2,14912E-05 |
| 5,39178E-05 | 0,267978022 | -2,23999E-05 | 0,538956348 | 1,01653E-05 | 0,63900028 | -7,3303E-06 | 0,736623776 | 1 | 1 | 1 | 1 | Bacteria | Firmicutes | Erysipelotrichia | Erysipelotrichales | Erysipelotrichaceae | Erysipelotrichaceae_UCG-004 | Erysipelotrichaceae_UCG-004 | 2,10526E-05 |
| 1,13836E-05 | 0,668555096 | 5,86373E-07 | 0,977139851 | -1,14657E-05 | 0,427869449 | 1,75695E-05 | 0,226041337 | 1 | 1 | 1 | 1 | Bacteria | Proteobacteria | Deltaproteobacteria | Desulfovibrionales | Desulfovibrionaceae | Mailhella | Mailhella | 2,10526E-05 |
| 4,48195E-05 | 0,002966634 | -1,57733E-05 | 0,158352955 | 3,9502E-06 | 0,56274405 | -3,92835E-06 | 0,566928976 | 0,495427929 | 1 | 1 | 1 | Bacteria | Proteobacteria | Gammaproteobacteria | Betaproteobacteriales | Burkholderiaceae | Oxalobacter | Oxalobacter | 2,0614E-05 |
| 5,19587E-06 | 0,825518992 | 1,71071E-05 | 0,177924697 | -2,26048E-05 | 0,101859826 | 1,05687E-05 | 0,448386388 | 1 | 1 | 1 | 1 | Bacteria | Proteobacteria | Gammaproteobacteria | Betaproteobacteriales | Neisseriaceae | Eikenella | Eikenella | 1,88596E-05 |
| 3,48515E-06 | 0,898296445 | -4,28633E-06 | 0,822465708 | 8,97232E-06 | 0,644242805 | 2,88131E-05 | 0,141637642 | 1 | 1 | 1 | 1 | Bacteria | Firmicutes | Clostridia | Clostridiales | Peptostreptococcaceae | Clostridioides | Clostridioides | 1,84211E-05 |
| 5,00247E-06 | 0,690097212 | 1,32474E-05 | 0,160443946 | -4,1315E-07 | 0,941328714 | -7,55293E-06 | 0,182227781 | 1 | 1 | 1 | 1 | Bacteria | Actinobacteria | Coriobacteriia | Coriobacteriales | Atopobiaceae | Atopobium | Atopobium | 1,75439E-05 |
| 6,69668E-05 | 0,186339374 | -4,82617E-05 | 0,202570244 | 1,62271E-05 | 0,45999869 | 1,32504E-05 | 0,548815447 | 1 | 1 | 1 | 1 | Bacteria | Firmicutes | Clostridia | Clostridiales | Lachnospiraceae | Lachnospiraceae_NK3A20_group | Lachnospiraceae_NK3A20_group | 1,75439E-05 |
| 4,4763E-05 | 0,034664754 | -9,10877E-06 | 0,555896135 | -1,77068E-05 | 0,246020743 | -1,53936E-06 | 0,919906945 | 1 | 1 | 1 | 1 | Bacteria | Firmicutes | Clostridia | Clostridiales | Lachnospiraceae | Moryella | Moryella | 1,71053E-05 |
| 6,17263E-05 | 0,132492108 | -4,36106E-05 | 0,155018089 | 1,00087E-05 | 0,575481261 | 1,44848E-05 | 0,42086639 | 1 | 1 | 1 | 1 | Bacteria | Actinobacteria | Actinobacteria | Actinomycetales | Actinomycetaceae | Arcanobacterium | Arcanobacterium | 1,66667E-05 |
| -2,81079E-05 | 0,245339528 | 2,73723E-05 | 0,130551576 | -1,21143E-05 | 0,248595028 | 1,38483E-05 | 0,190258618 | 1 | 1 | 1 | 1 | Bacteria | Proteobacteria | Gammaproteobacteria | Enterobacteriales | Enterobacteriaceae | Atlantibacter | Atlantibacter | 1,66667E-05 |
| 5,25293E-05 | 0,285552442 | -2,3094E-05 | 0,529589919 | 1,83117E-05 | 0,390776828 | -1,57206E-05 | 0,464063401 | 1 | 1 | 1 | 1 | Bacteria | Bacteroidetes | Bacteroidia | Flavobacteriales | Flavobacteriaceae | Flavobacterium | Flavobacterium | 1,57895E-05 |
| 5,18785E-05 | 0,177053052 | -1,32607E-05 | 0,643768026 | -1,00001E-05 | 0,548135659 | -1,36849E-05 | 0,414358102 | 1 | 1 | 1 | 1 | Bacteria | Actinobacteria | Actinobacteria | Actinomycetales | Actinomycetaceae | Mobiluncus | Mobiluncus | 1,53509E-05 |
| 4,20177E-05 | 0,011271443 | -1,19412E-05 | 0,332330698 | -1,20899E-05 | 0,091616322 | -3,08165E-06 | 0,668299976 | 1 | 1 | 1 | 1 | Bacteria | Firmicutes | Clostridia | Clostridiales | Lachnospiraceae | Lachnospiraceae_UCG-002 | Lachnospiraceae_UCG-002 | 1,53509E-05 |
| 3,3381E-05 | 0,002241397 | -7,37013E-06 | 0,358436863 | -4,90174E-06 | 0,303439921 | -5,31735E-06 | 0,26725701 | 0,385520287 | 1 | 1 | 1 | Bacteria | Firmicutes | Clostridia | Clostridiales | Ruminococcaceae | Phocea | Phocea | 1,53509E-05 |
| 5,10702E-05 | 0,285552442 | -2,24525E-05 | 0,529589919 | 1,7803E-05 | 0,390776828 | -1,52839E-05 | 0,464063401 | 1 | 1 | 1 | 1 | Bacteria | Proteobacteria | Gammaproteobacteria | Betaproteobacteriales | Burkholderiaceae | Comamonas | Comamonas | 1,53509E-05 |
| 2,26533E-05 | 0,098548588 | 1,71575E-06 | 0,866670727 | -1,23586E-06 | 0,835003694 | -1,10928E-05 | 0,064251236 | 1 | 1 | 1 | 1 | Bacteria | Actinobacteria | Actinobacteria | Actinomycetales | Actinomycetaceae | F0332 | F0332 | 1,49123E-05 |
| 3,04986E-05 | 0,0410599 | -1,57989E-05 | 0,155840243 | 5,10775E-06 | 0,428646338 | 4,64932E-06 | 0,474052661 | 1 | 1 | 1 | 1 | Bacteria | Firmicutes | Clostridia | Clostridiales | Christensenellaceae | Catabacter | Catabacter | 1,44737E-05 |
| 4,80591E-05 | 0,211052609 | -1,44959E-05 | 0,613270903 | -7,69129E-06 | 0,644149357 | -1,0189E-05 | 0,543291403 | 1 | 1 | 1 | 1 | Bacteria | Bacteroidetes | Bacteroidia | Bacteroidales | Prevotellaceae | Prevotella_6 | Prevotella_6 | 1,40351E-05 |
| 2,31258E-05 | 0,046855619 | -5,97133E-06 | 0,490654573 | -3,73285E-06 | 0,45803453 | 1,89758E-06 | 0,707683881 | 1 | 1 | 1 | 1 | Bacteria | Firmicutes | Clostridia | Clostridiales | Christensenellaceae | Christensenella | Christensenella | 1,40351E-05 |
| 4,35938E-05 | 0,155770121 | -1,14075E-05 | 0,618559715 | -6,82813E-06 | 0,607738589 | -1,1264E-05 | 0,400418961 | 1 | 1 | 1 | 1 | Bacteria | Actinobacteria | Actinobacteria | Actinomycetales | Actinomycetaceae | Varibaculum | Varibaculum | 1,35965E-05 |
| -3,89842E-05 | 0,335609249 | 4,35757E-05 | 0,150427039 | 3,12123E-05 | 0,076396526 | -3,29365E-05 | 0,063292366 | 1 | 1 | 1 | 1 | Bacteria | Proteobacteria | Gammaproteobacteria | Enterobacteriales | Enterobacteriaceae | Kluyvera | Kluyvera | 1,31579E-05 |
| 3,00064E-05 | 0,366333721 | -7,48494E-06 | 0,762876907 | 1,00144E-05 | 0,487076654 | -1,45064E-05 | 0,317500688 | 1 | 1 | 1 | 1 | Bacteria | Firmicutes | Clostridia | Clostridiales | Lachnospiraceae | Herbinix | Herbinix | 1,22807E-05 |
| 2,62243E-05 | 0,050578881 | -1,52516E-05 | 0,127591327 | 5,9413E-06 | 0,305935306 | 5,09869E-06 | 0,382561442 | 1 | 1 | 1 | 1 | Bacteria | Firmicutes | Bacilli | Lactobacillales | Lactobacillaceae | Pediococcus | Pediococcus | 1,18421E-05 |
| 3,50195E-05 | 0,285552442 | -1,5396E-05 | 0,529589919 | 1,22078E-05 | 0,390776828 | -1,04804E-05 | 0,464063401 | 1 | 1 | 1 | 1 | Bacteria | Firmicutes | Bacilli | Bacillales | Planococcaceae | Sporosarcina | Sporosarcina | 1,05263E-05 |
| 3,02727E-05 | 0,042594941 | -9,8817E-06 | 0,388966222 | 5,09803E-08 | 0,99609091 | -4,60882E-06 | 0,658441038 | 1 | 1 | 1 | 1 | Bacteria | Bacteroidetes | Bacteroidia | Bacteroidales | Prevotellaceae | Prevotellaceae_UCG-003 | Prevotellaceae_UCG-003 | 1,00877E-05 |
| 3,40916E-05 | 0,152283118 | -8,7142E-06 | 0,623772342 | -6,57152E-06 | 0,524123393 | -8,99294E-06 | 0,386628783 | 1 | 1 | 1 | 1 | Bacteria | Firmicutes | Clostridia | Clostridiales | Family_XI | Murdochiella | Murdochiella | 1,00877E-05 |
| 3,43956E-05 | 0,132580288 | -1,47627E-05 | 0,387010777 | 6,00143E-06 | 0,544535402 | -7,30965E-06 | 0,463469134 | 1 | 1 | 1 | 1 | Bacteria | Firmicutes | Clostridia | Clostridiales | Lachnospiraceae | Lachnospiraceae_NC2004_group | Lachnospiraceae_NC2004_group | 1,00877E-05 |

Slooter *et al. BMC Surgery* (2020) 20:240


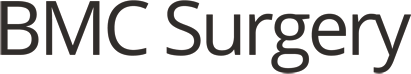


<https://doi.org/10.1186/s12893-020-00890-w>

STUDY PROTOCOL Open Access

IMARI: multi-Interventional program for prevention and early Management of Anastomotic leakage after low anterior resection in Rectal cancer patIents: rationale and study protocol

M. D. Slooter^1†^, K. Talboom^1*†^[
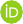
](http://orcid.org/0000-0002-2007-4934), S. Sharabiany^1^, C. P. M. van Helsdingen^1^, S. van Dieren^1^, C. Y. Ponsioen^2^, C. Y. Nio^3^,

[
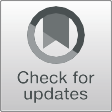
](http://crossmark.crossref.org/dialog/?doi=10.1186/s12893-020-00890-w&domain=pdf)

E. C. J. Consten^4^, J. H. Wijsman^5^, M. A. Boermeester^1^, J. P. M. Derikx^6^, G. D. Musters^1^, W. A. Bemelman^1^, P. J. Tanis^1^,

R. Hompes^1*^ and on behalf of the IMARI-study group

Abstract

Background: Anastomotic leakage (AL) is still a common and feared complication after low anterior resection (LAR) for rectal cancer. The multifactorial pathophysiology of AL and lack of standardised treatment options requires a multi-modal approach to improve long-term anastomotic integrity. The objective of the IMARI-trial is to determine whether the one-year anastomotic integrity rate in patients undergoing LAR for rectal cancer can be improved using a multi-interventional program.

Methods: IMARI is a multicentre prospective clinical effectiveness trial, whereby current local practice (control cohort) will be evaluated, and subsequently compared to results after implementation of the multi-interventional program (intervention cohort). Patients undergoing LAR for rectal cancer will be included. The multi-interventional program includes three preventive interventions (mechanical bowel preparation with oral antibiotics, tailored full splenic flexure mobilization and intraoperative fluorescence angiography using indocyanine green) combined with a standardised pathway for early detection and active management of AL. The primary outcome is anastomotic integrity, confirmed by CT-scan at one year postoperatively. Secondary outcomes include incidence of AL, protocol compliance and association with AL, temporary and permanent stoma rate, reintervention rate, quality of life and functional outcome. Microbiome analysis will be conducted to investigate the role of the rectal microbiome in AL. In a Dutch nationwide study, the AL rate was 20%, with anastomotic integrity of 90% after one year. Based on an expected reduction of AL due to the preventive approaches of 50%, and increase of anastomotic integrity by a standardised pathway for early detection and active management of AL, we hypothesised that the anastomotic

(Continued on next page)

* Correspondence: [k.talboom@amsterdamumc.nl](mailto:k.talboom@amsterdamumc.nl); [r.hompes@amsterdamumc.nl](mailto:r.hompes@amsterdamumc.nl)

^†^M. D. Slooter and K. Talboom contributed equally to this work.

^1^Department of Surgery, Amsterdam UMC, Location AMC, Amsterdam, The Netherlands

Full list of author information is available at the end of the article


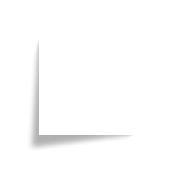

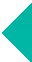


© The Author(s). 2020 Open Access This article is licensed under a Creative Commons Attribution 4.0 International License, which permits use, sharing, adaptation, distribution and reproduction in any medium or format, as long as you give appropriate credit to the original author(s) and the source, provide a link to the Creative Commons licence, and indicate if changes were made. The images or other third party material in this article are included in the article's Creative Commons licence, unless indicated otherwise in a credit line to the material. If material is not included in the article's Creative Commons licence and your intended use is not permitted by statutory regulation or exceeds the permitted use, you will need to obtain permission directly from the copyright holder. To view a copy of this licence, visit <http://creativecommons.org/licenses/by/4.0/>. The Creative Commons Public Domain Dedication waiver (<http://creativecommons.org/publicdomain/zero/1.0/>) applies to the data made available in this article, unless otherwise stated in a credit line to the data.

(Continued from previous page)

integrity rate will increase from 90 to 97% at one year. An improvement of 7% in anastomotic integrity at one year was considered clinically relevant. A total number of 488 patients (244 per cohort) are needed to detect this difference, with 80% statistical power.

Discussion: The IMARI-trial is designed to evaluate whether a multi-interventional program can improve long-term anastomotic integrity after rectal cancer surgery. The uniqueness of IMARI lies in the multi-modal design that addresses the multifactorial pathophysiology for prevention, and a standardised pathway for early detection and active treatment of AL.

Trial registration: Trialregister.nl ([NL8261](https://www.trialregister.nl/trial/8261)), January 2020.

Keywords: Rectal cancer, Anastomotic leakage, Total Mesorectal excision, Prevention, Anastomotic salvage

Background

Anastomotic leakage (AL) is still a common and feared complication after low anterior resection (LAR) for rec-tal cancer. A nationwide cross-sectional study with more than 3-years follow-up revealed an overall incidence of 20% [[1](#bookmark3)]. Occurrence of AL leads to significant increase of postoperative morbidity, prolonged hospital stay, in-creased healthcare costs, and adversely affects onco-logical and functional outcome with an increased risk of a permanent stoma [[2](#bookmark3)–[4](#bookmark3)]. The underlying aetiology for AL is a complex multifactorial mix of both modifiable and non-modifiable risk factors that relate to various pa-tient- and tumour characteristics, neo-adjuvant proto-cols and intraoperative technical aspects [[1](#bookmark3), [5](#bookmark3)–[7](#bookmark3)]. Examples of modifiable surgical factors include tension on the anastomosis and anastomotic perfusion. Lately, the impact of the gut microbiome on AL has been stud-ied and a pivotal role seems plausible [[8](#bookmark3), [9](#bookmark3)].

While better understanding and modification of risk fac-tors will undoubtedly drive AL rates down, the risk will never be completely non-existent as a result of non-modifiable and currently unknown factors. Hence, besides focus on prevention, limiting the impact of AL is equally important and can be achieved by early detection and ap-propriate management. However, no international con-sensus exists on a diagnostic pathway for early detection of AL, even though evidence is building for the use of C-reactive protein (CRP) in the early postoperative period [[10](#bookmark3), [11](#bookmark3)]. Regarding management of AL, this usually in-volves a deviating ileostomy if not yet performed primar-ily, in combination with “passive” drainage of the abscess cavity via transanal or percutaneous route [[1](#bookmark3), [12](#bookmark3)]. Using this aforementioned approach, almost half of the leaks do not heal and may require major salvage surgery, including the creation of a permanent stoma [[1](#bookmark3), [12](#bookmark3)].

We hypothesised that a multi-interventional program with a focus on prevention, diagnosis and management of AL would improve the one-year anastomotic integrity rate in patients undergoing LAR for rectal cancer. In the IMARI trial, the chosen set of interventions aiming at reduced risk of AL were: (1) mechanical bowel

preparation (MBP) with oral antibiotics (AB) to optimise the microbiome [[13](#bookmark3)–[16](#bookmark3)]; (2) splenic flexure mobilization to optimise a tension-free anastomosis [[17](#bookmark3)]; (3) intraop-erative real-time fluorescence angiography (FA) using in-docyanine green (ICG) to assess adequate perfusion [[18](#bookmark3), [19](#bookmark3)]. These preventive measures are combined with clin-ical pathways for early detection and “active” manage-ment of AL. Serial CRP measurements in the early postoperative period in combination with a CT-scan with rectal contrast will be employed for early detection. On confirmation of AL, endoscopic vacuum-assisted closure therapy (EVAC) of the abscess cavity is initiated to control pelvic sepsis followed by early transanal clos-ure or restorative re-do surgery to restore anastomotic integrity. This quality controlled multi-interventional program will be implemented within existing institu-tional enhanced recovery programs and prehabilitation initiatives.

Methods

This study protocol is written in accordance with the SPIRIT guidelines [[20](#bookmark3), [21](#bookmark3)] and the SPIRIT checklist is provided in Appendix 1.

Study objectives

The primary objective of this study is to determine whether the one-year anastomotic integrity rate in pa-tients undergoing LAR for rectal cancer can be improved using a multi-interventional program which includes:

(1) MBP/AB; (2) tailored full splenic flexure mobilisa-tion; (3) intraoperative FA using ICG ; (4) routine CRP measurements postoperatively and CT-scan with rectal contrast on indication; (5) EVAC with early transanal closure of the anastomotic defect or restorative re-do surgery.

Secondary objectives include the evaluation of the multi-interventional program on the AL rate and quality of life until one year after the index operation, and the establishment of the IMARI biobank. The rationale for sample collection in the IMARI biobank is to investigate the role of the rectal microbiome in AL.

Study design

The IMARI trial is a multicentre prospective clinical ef-fectiveness trial, whereby current local practice (control cohort) will be evaluated, and subsequently compared to results after implementation of the multi-interventional program (intervention cohort). The flow diagram for the study is shown in Fig. [1](#bookmark0).

Ethical consideration

The trial will be conducted according to Good Clinical Practice guidelines and the principles of the declaration of Helsinki (2013, [[22](#bookmark4)]). This study is approved by the Medical Ethical Committee and Biobank committee of the Amsterdam UMC, location AMC. The protocol is registered by the Dutch Central Committee on Research Involving Human Subjects (NL67600.018.18) and is sub-mitted to the trialregister.nl database (NL8261).

Study population

Eligibility criteria for study participation are: (1) planned to undergo LAR for either one of the following diagno-ses: a) primary rectal cancer as defined by the inter-national consensus definition for rectal cancer [[23](#bookmark4)] or b) regrowth of rectal cancer in a watch and wait protocol or c) completion/salvage surgery after local excision for rectal cancer; (2) willing to complete quality of life

questionnaires and comply with schedule of outpatient follow-up visits; (3) ≥ 18 years old.

A subject is not eligible for inclusion in case of pres-ence of one of the following exclusion criteria: (1) LAR without colorectal or coloanal anastomosis; (2) locally advanced rectal cancer, expected to require beyond-total mesorectal excision approach or multi-visceral excision;

(3) synchronous colonic resections.

Informed consent procedure

Patients meeting all eligibility criteria stated above will be informed on the trial at the outpatient clinic by a member of the research team. Written informed consent will be obtained for participation in the trial and separ-ate consent obtained for storage of samples in the IMARI biobank. Every included patient will be assigned a three-digit study number and only local sites have ac-cess to a decryption code.

Study outline

*Control cohort*

The study will start in all participating hospitals with ac-crual into the control cohort, whereby patients will re-ceive care according to standard local protocol. The local protocol may well include one or more compo-nents of the multi-interventional program and this will


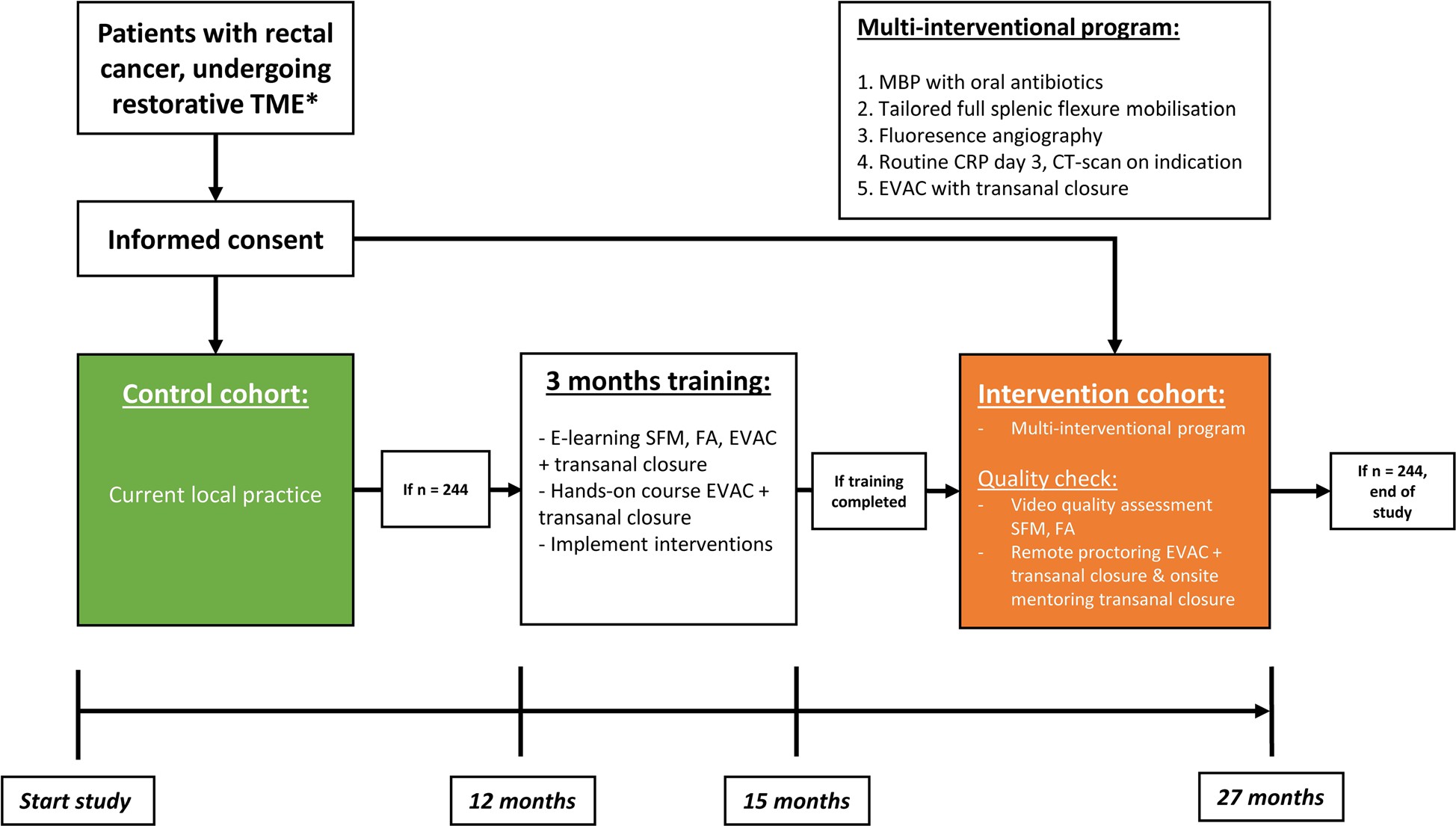


Fig. 1 Flow diagram study. MBP, Mechanical Bowel preparation; CRP, C-reactive protein; CT, computed tomography; EVAC, endoscopic vacuum-assisted closure; FA, Fluorescence angiography; SFM, Splenic flexure mobilisation; TME, Total Mesorectal Excision

be recorded in the case-report form (CRF) for each patient.

*Intervention cohort*

When accrual of the control cohort has been completed (*n* = 244, Fig. [1](#bookmark0)), all participating hospitals will start a training period of 3 months before implementation of the multi-interventional program and accrual of patients into the intervention cohort. A standardised protocol for MBP/AB and postoperative surveillance of patients for AL will be distributed among centres, enabling timely implementation before start of the intervention cohort. Staff from participating centres will be trained via online educational modules and hands-on training sessions on tailored splenic flexure mobilization, intraoperative FA and EVAC management of AL combined with early sur-gical closure of anastomotic defects. Random checks of procedural videos and use of a system for remote proc-toring will be employed to ensure quality control throughout the entire trial period.

*Multi-interventional program*

Mechanical bowel preparation with oral antibiotics MBP will start the day before surgery by oral administra-tion of 2 l of polyethylene glycol (Moviprep®) or sodium phosphate. Based on the results from the SELECT-trial

[[16](#bookmark3)] and unpublished work from the pre-caution trial [[24](#bookmark4)], 10 ml of selective digestive decontamination (SDD) solution will be administered four times daily during the three days prior to surgery. The SDD suspension (10 ml) will contain: colistine 100 mg, tobramycine 80 mg and amphotericine B 500 mg.

Tailored full splenic flexure mobilization For low rec-tal cancers, defined according to the LOREC definition, a full splenic flexure mobilisation is mandatory [[25](#bookmark4), [26](#bookmark4)]. For all other rectal cancers a full splenic flexure mobil-isation is at the discretion of the operating surgeon. Full splenic flexure mobilisation entails the following essen-tial and mandatory steps: (1) division of the inferior mes-enteric vein at the lower border of the pancreas just lateral to the angle of Treitz; (2) full release of the distal transverse colonic mesentery from the body and tail of the pancreas; (3) division of the gastro-colic ligament to release omentum from distal transverse colon. These steps can be completed either in a medial to lateral or lateral to medial approach.

Intraoperative fluorescence angiography using indocyanine green Intraoperative FA using ICG will be performed in all patients before and after construc-tion of the anastomosis using a standard intravenous injection of ICG (0.1 mg/kg/bolus). Near infrared

imaging can be performed by different imaging plat-forms, and all relevant FA characteristics will be re-corded in the CRF. The first assessment is done after rectal mobilisation, but prior to bowel division. The proximal colon will be assessed under conventional white light and the point of planned transection will be marked. Subsequently, FA will be performed using either an intracorporeal or extracorporeal FA tech-nique. The decision whether or not to change the planned anastomotic site will be made according to the surgeon’s subjective interpretation of FA.

Anastomotic reconstruction is performed according to the surgeon’s preference, followed by an intracorporeal or intraluminal FA assessment of the anastomosis after a second bolus of ICG. Any anastomotic revision, or add-itional manipulation of the anastomosis (i.e. sutures) will be recorded. The creation of a deviating stoma will be at the surgeon’s discretion. A third dose of ICG is allowed, if deemed necessary by the operating surgeon.

Routine CRP measurement CRP measurement will be performed routinely on day 3 postoperatively. A CRP level above the threshold of 172 mg/l [[10](#bookmark3)], combined with any clinical aberrant observations, will trigger a CT Abdomen with rectal contrast. Otherwise, CRP measure-ment will be repeated at day 4 postoperatively. In case of a stable or higher CRP level, a CT abdomen with rectal contrast will be performed to exclude AL, irrespective of clinical findings. Any extraluminal air and/or fluid at the level of the anastomosis will at least be considered as suspicious of AL based on CT, requiring further investi-gation. Any extravasation of contrast will be defined as clear AL. The algorithm for clinical decision making ac-cording to CRP level is displayed in Fig. [2](#bookmark1).

Endoscopic vacuum-assisted drainage with early transanal closure of the anastomotic defect When the CT-scan reveals clear AL, clinical management depends on the presence of a primary diverting stoma. If not cre-ated primarily, a diverting ileostomy will be constructed with abdominal lavage in case of purulent or fecal peri-tonitis, preferably using a laparoscopic approach, and combined with intraoperative endoscopic assessment of the anastomosis with EVAC if indicated. In patients with primary diversion, endoscopic assessment of the anasto-mosis can be performed under general anaesthesia, espe-cially if surgical management of peritonitis is required, or under sedation at the endoscopy room. For a pelvic fluid collection on CT without any obvious extraluminal contrast, an endoscopy is preferred as first step to assess whether an actual defect can be identified before return to theatre for diversion. At endoscopy, potential signs of ischaemia and characteristics of the anastomotic defect


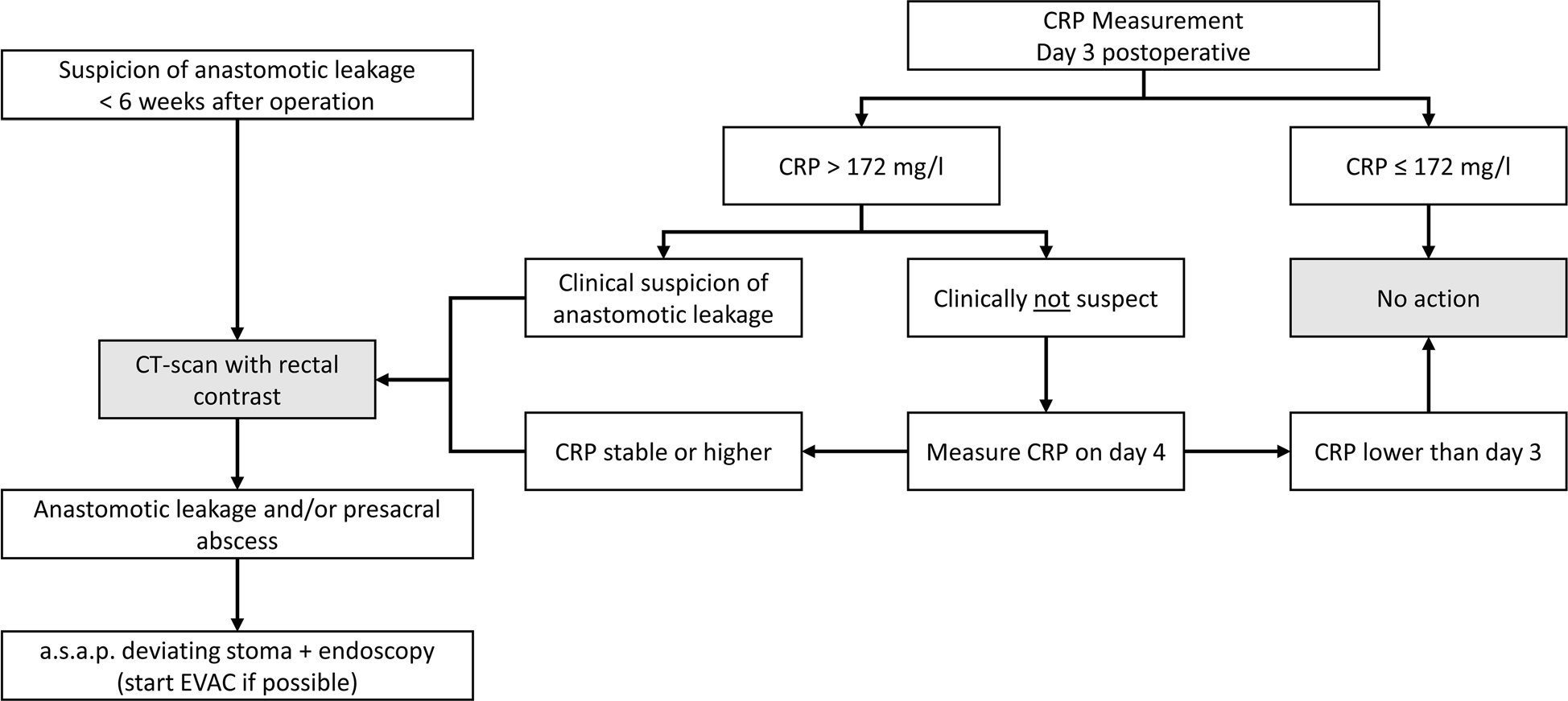


Fig. 2 Flow diagram postoperative algorithm


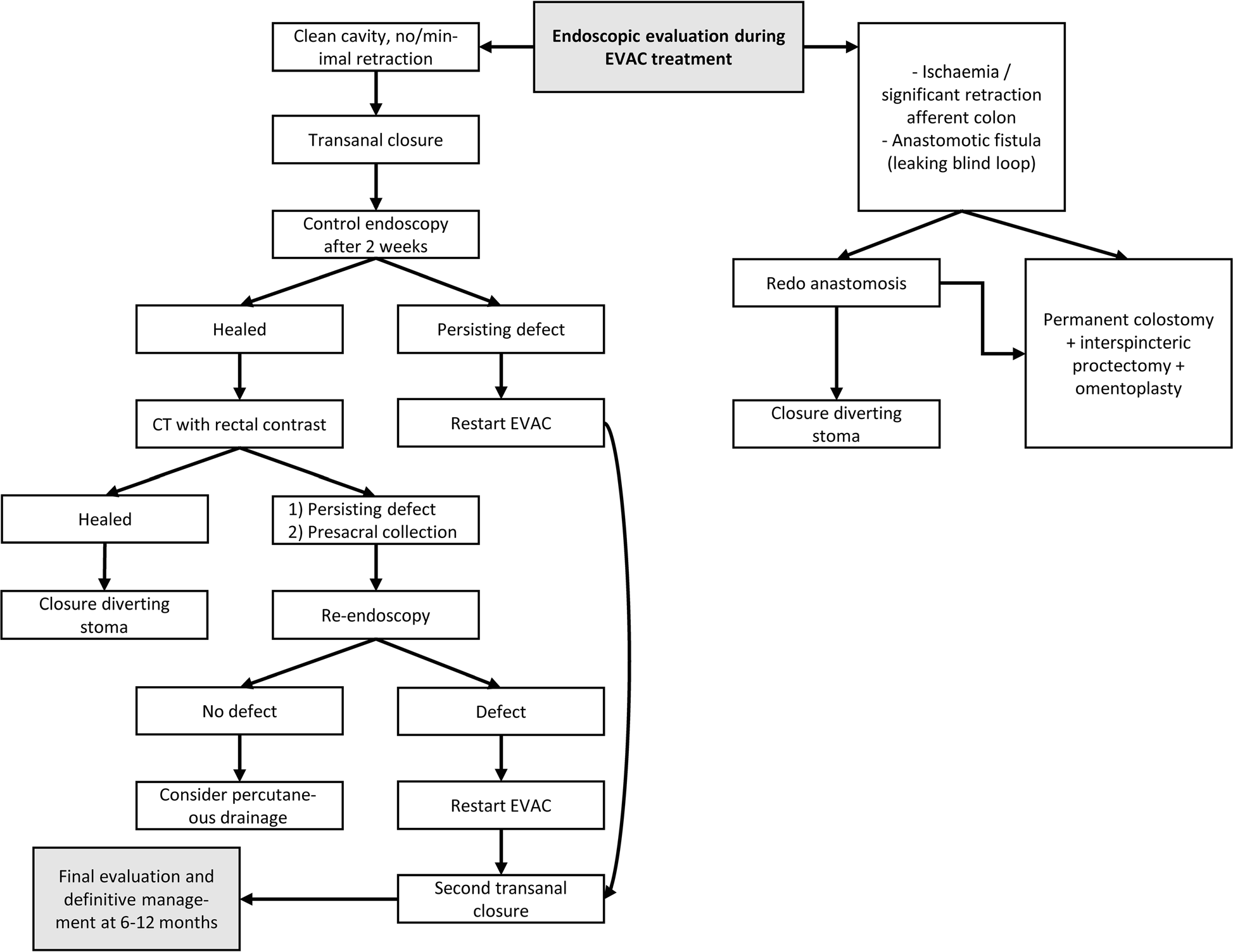


Fig. 3 Flow diagram pro-active leak management

(extent circular dehiscence, retraction) will determine further steps to control pelvic sepsis (Fig. [3](#bookmark1)).

Patients deemed suitable for EVAC will have endo-sponge exchanges every 3–4 days, with assessment of the anastomotic defect and associated cavity by the gastro-enterologist and/or surgeon. Usually after two to four endosponge exchanges, the anastomotic defect should be ready to be closed transanally as previously described [[27](#bookmark4)–[29](#bookmark4)]. The transanal closure will be checked by endos-copy two weeks postoperatively. If no defect is identified at endoscopy, a further assessment will follow by CT with rectal contrast. At the time of endoscopy a CRP check will also be included.

If the initial endoscopic evaluation reveals ischaemia or significant retraction of the afferent colon, a different pathway will be followed: (1) early or late re-do of the anastomosis, with use of EVAC for initial control of pel-vic sepsis; or (2) take down of the anastomosis; preferred technique will be intersphincteric resection of the rectal remnant, permanent colostomy and filling of the pelvis with an omentoplasty.

At any point in time, participating centres can contact the initiating centre for advice, assessment of endoscopy images and the most appropriate further step in man-agement of the AL and sepsis.

Outcomes

The primary outcome of this study is anastomotic integ-rity one year after the index operation. This will be de-termined in all included patients by CT-scan at one year as part of regular follow-up of patients after rectal can-cer surgery [[30](#bookmark4)].

Secondary outcomes include: (1) incidence of AL within 30 days, 90 days, and one year post-operative; (2) protocol compliance to any intervention; (3) protocol compliance in association to AL; (4) changes in rectal microbiome and association with AL; (5) permanent stoma rate; (6) temporary stoma rate and total time of having a stoma during one year; (7) length of hospital stay after index surgery and total stay during one year;

(8) overall and stoma-related readmission and reinter-vention rates; (9) quality of life (EQ-5D, QLQ-C30, QLQ-CR29, 10) bowel, urinary and sexual function (LARS, UDI-6, IIQ-7, IIEF for male and MFSFQ for fe-male) pre-operatively, at 90 days and one year; (11) diag-nostic accuracy of serial CRP at day 3–4; (12) efficacy of EVAC with early transanal closure of the anastomotic defect; (13) change of management related to FA: site of proximal bowel division used for anastomosis, re-do anastomosis, reinforcement of anastomosis after con-struction, decision for diverting stoma, or decision for a non-restorative procedure; (14) operative and post-operative complications within 90 days of index surgery;

(15) 1-year local recurrence and overall survival rate.

To assess the rectal microbiome, the following samples are collected for the IMARI biobank: stool samples be-fore start MBP/AB and at day 4 postoperative, the anas-tomotic donut (colonic side) from the operation, intraoperative rectal swab from the anastomotic site, and for patients that develop AL an endoscopic rectal swab from the abscess cavity. Samples will be stored centrally in the IMARI biobank at the Tytgat Institute in the Amsterdam UMC, location AMC. Microbiota profiling will be done using an Illumina Miseq platform. In addition, metatranscriptomics will be performed on se-lected samples to look for presence and activity of col-lagenolytic *Enterococcus faecalis* and additional detrimental species for anastomotic integrity.

Collection points of all outcomes are summarised in

Table [1](#bookmark2).

Sample size calculation

In a Dutch nationwide study, the AL rate was 20%, with anastomotic integrity of 90% after one year [[1](#bookmark3)]. Meta-analysis of MBP/AB revealed that preoperative antibi-otics were associated with lower AL rates (OR 0.59, 0.53–0.67; *p* < 0.001) [[14](#bookmark3)]. Pooled analysis of studies using routine FA showed an OR of 0.34 (0.16–0.74; *p* = 0.006) [[18](#bookmark3)]. Together with full splenic flexure mobilisa-tion, the estimated reduction in AL rate is 50%. In the CLEAN-study, treatment with EVAC and early surgical closure resulted in anastomotic healing in two thirds of the patients within the first year [[31](#bookmark4)]. Therefore, we hypothesised that the combination of all interventions will increase the anastomotic integrity rate from 90 to 97% at one year. Applying a Fisher exact test with a two-sided 0.05 significance level and 80% power, and with an estimated drop-out of 10%, a total number of 488 pa-tients (244 per cohort) are needed to be able to detect a 7% increase in anastomotic integrity by implementation of the combined interventions.

Statistical analysis

The primary endpoint, anastomotic integrity, will be compared between the two trial cohorts using a two-sided Fisher exact test. AL rates will be compared be-tween the cohorts using generalised estimating equations model adjusting for stratification factors. This approach will be used to test the two-sided hypothesis that the AL rate is equal in both cohorts (i.e. an odds ratio of 1), considering the 95% confidence interval and a *p*-value of

0.05. Other secondary endpoints with binary measures will be analysed using multi-variable logistic regression adjusting for stratification factors. Secondary endpoints with continuous measures will be analysed using linear regression models adjusting for stratification factors. When the data is not normally distributed, the data will be transformed to achieve normal distribution. The

Table 1 Timing of enrollment, interventions and assessments. IC, informed consent

|  | 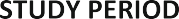 | | | | | | | | | | |
| --- | --- | --- | --- | --- | --- | --- | --- | --- | --- | --- | --- |
|  |  | | | |  | 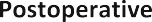 | | | | | |
|  | 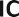 |  |  |  |  |  |  |  | 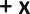 | 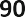 | 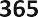 |
| 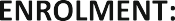 | | | | | | | | | | | |
| 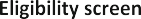 | 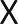 |  |  |  |  |  |  |  |  |  |  |
| 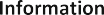 | 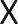 |  |  |  |  |  |  |  |  |  |  |
| 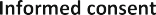 | 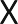 |  |  |  |  |  |  |  |  |  |  |
|  | | | | | | | | | | | |
| 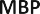 |  |  |  | 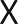 |  |  |  |  |  |  |  |
| 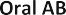 |  |  |  |  |  |  |  |  |  |  |  |
| 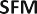 |  |  |  |  | 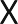 |  |  |  |  |  |  |
| 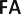 |  |  |  |  | 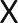 |  |  |  |  |  |  |
| 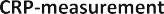 |  |  |  |  |  |  | 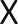 |  |  |  |  |
|  |  |  |  |  |  |  |  |  | 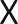 |  |  |
| 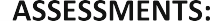 | | | | | | | | | | | |
| 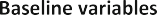 |  | 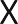 |  |  |  |  |  |  |  |  |  |
| 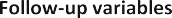 |  |  |  |  | 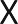 |  |  |  | 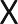 | 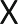 | 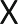 |
|  |  | 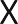 |  |  | 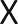 | 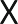 |  | 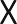 | 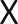 |  |  |
|  |  | 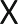 |  |  |  |  |  |  |  | 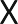 | 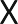 |
| 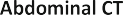 |  |  |  |  |  |  |  |  | 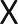 |  | 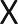 |

secondary endpoint ‘duration of temporary stoma’ will be analysed using a cox-regression model with adjust-ing for stratification factors. Quality of life and func-tion outcome will be calculated as domain and summarised scores according to the manuals, and graphically represented across all time points. Com-parisons of questionnaire outcomes will be analysed using linear mixed models. Statistical analyses will be performed using the latest version of SPSS software for Windows.


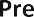

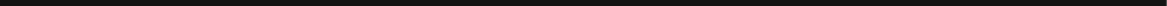

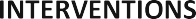

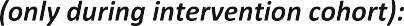

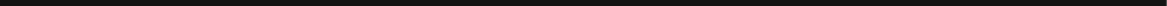

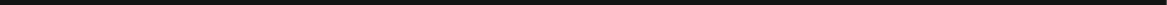


The statistical analysis plan will be finalised before data is locked for analysis, and decision will be made on stratification factors and planned subgroup analysis, and on how to deal with application of components of the multi-interventional program in the control cohort, protocol violations, and baseline imbalance.

Safety reporting

This IMARI trial is considered a low-risk study, because any of the interventions are already being used in

routine daily practice. Serious adverse events will not be reported for the control cohort, since patients will re-ceive standard care. Serious adverse events will be re-corded until 30 days after index surgery or any study related procedure for the intervention cohort.

Data handling and monitoring

Data will be digitally collected using the electronic data management system Castor EDC ([www.castoredc.com](http://www.castoredc.com/)). In all participating hospitals, one surgeon acts as local investigator who is primarily responsible for execution of trial interventions, and for accuracy and completeness of the CRF. Quality of life questionnaires will be col-lected through the data collection initiative of the Pro-spective Dutch ColoRectal Cancer (PLCRC) group ([clinicaltrials.gov](http://clinicaltrials.gov/) NCT02070146). This study will be monitored as described in a monitoring plan by an inde-pendent monitor to ensure quality and adherence to the protocol. If patients are only willing to participate in the

IMARI-trial, questionnaires will be collected by the investigators.

Public disclosure and publication policy

IMARI was registered at the trialregister.nl database (NL8261). The results of IMARI will be submitted to a peer-reviewed journal regardless of study outcome. Co-authorship will be based on the international ICMJE guidelines. Besides the key authors (coordinating investi-gators as first authors and principal investigators as se-nior authors), authorship is granted to the local investigator of each centre when at least ten patients are included in the trial and when substantial contribution to the trial is made.

Discussion

In contrast to improvements over the last decades re-garding oncological outcomes after rectal cancer surgery, AL and ensuing long-term sequelae remain common. A cross-sectional study in the Netherlands revealed an AL rate of 20% after long-term follow-up, with nearly half of AL not healing and giving rise to a chronic sinus. In the IMARI trial we propose a multi-interventional program, not only being designed to reduce AL, but also to in-crease the chance of long-term anastomotic integrity. The uniqueness of the IMARI trial lies in the multi-modal design that addresses the multifactorial patho-physiology, early detection and active treatment of AL.

Thus far, many risk factors have been associated with AL and a complex multifactorial pathophysiology has emerged [[1](#bookmark3), [5](#bookmark3)–[7](#bookmark3), [9](#bookmark3)]. Most interventional studies up till now only evaluated the impact of a single risk factor on AL [[16](#bookmark3), [17](#bookmark3), [32](#bookmark4), [33](#bookmark4)]. The IMARI trial addresses three modifiable risk factors to ensure a tension-free, adequate perfused anastomosis, under optimal condition of the microbiome: (1) MBP/AB that could lead to a reduction in AL by reduction of the fecal bulk and bacterial load [[13](#bookmark3)–[16](#bookmark3)]; (2) Splenic flexure mobilization to optimise a tension-free anastomosis, particularly for low rectal can-cer [[17](#bookmark3), [34](#bookmark4)]; (3) Intraoperative real-time FA using ICG to assesses adequate perfusion of the afferent colon and anastomosis. Routine use of this FA technology has been associated with reduced AL rates, although no data from large randomised controlled trials (RCT) are available [[18](#bookmark3), [19](#bookmark3)].

If AL occurs, prompt detection is crucial to allow for immediate treatment initiation and control of pelvic sep-sis. Rapid sepsis control avoids further morbidity and should also limit long-term functional sequelae. Al-though transanal and/or radiological transgluteal drain-age of pelvic sepsis does allow for some degree of sepsis control, leakage is not actively treated and the anasto-motic defect is not likely to heal spontaneously. In con-trast, after 2–4 EVAC exchanges, which takes

approximately 1–2 weeks, well vascularised granulation tissue is often visible inside the cavity. This allows for subsequent transanal closure of the anastomotic defect with a suction drain positioned behind the anastomosis with its tip inside the cavity, after which the cavity col-lapses and the neo-rectum expands [[29](#bookmark4), [31](#bookmark4)]. As such, EVAC in combination with early transanal closure al-lows for a more active, rapid control of pelvic sepsis and at the end mucosal approximation. This pathway should allow for more anastomoses to be preserved, prevent chronic presacral sinuses and improve functional out-comes by limiting peri-anastomotic fibrosis with preser-vation of compliance of the neo-rectum.

Even though RCTs are considered the most robust re-

search strategy for establishing a causal relationship, a comparative cohort design was chosen for the IMARI trial. In the setting of a classical RCT, contamination is likely to occur in the control arm. Surgeons are likely to change their daily practice, when observing benefits from the multi-interventional program. We consider this also a problem in a stepped-wedge cluster RCT, a fre-quently used variant of a classical RCT. Thus, a com-parative cohort design was selected in the form of a prospective clinical effectiveness trial, where crossover to the intervention cohort occurs after completion of ac-crual in the control cohort. Participating centres will simultaneous start recruitment for the intervention arm, after completion of a 3 month training period. Further-more, in the set-up of a clinical effectiveness trial the true impact of utilising the multi-interventional program can be evaluated under real conditions [[35](#bookmark4)].

For the purpose of the IMARI trial, a multidisciplinary

scientific study-group was composed, including surgeons from both academic and peripheral centres, gastroenter-ologists, radiologists, specialised nurses and researchers. In this way hospital-wide awareness is created and a broadly supported multi-modal approach was made possible.

Successful implementation of the IMARI multi-interventional program within existing enhanced recov-ery and prehabilitation programs would have a positive influence on morbidity, mortality, and possibly onco-logical outcomes. By increasing the chance of long-term anastomotic integrity and decreasing permanent stoma rates, the IMARI trial should contribute to a better quality of life for patients undergoing rectal cancer surgery.

Supplementary information

Supplementary information accompanies this paper at [https://doi.org/10.](https://doi.org/10.1186/s12893-020-00890-w) [1186/s12893-020-00890-w](https://doi.org/10.1186/s12893-020-00890-w).

Additional file 1.

Abbreviations

AB: Antibiotics; AL: Anastomotic Leakage; CRP: C-reactive protein;

CT: Computed tomography; EDC: Electronic Data Capture; EVAC: Endoscopic vacuum-assisted closure; FA: Fluorescence angiography; ICG: Indocyanine green; ICMJE : International Committee of Medical Journal Editors; LAR: Low anterior resection; LOREC : Low Rectal Cancer Development programme; MBP: Mechanical bowel preparation; PLCRC: Prospective Dutch ColoRectal Cancer; RCT: Randomized controlled trial; SDD: Selective digestive decontamination; SPIRIT: Standard Protocol Items: Recommendations for Interventional Trials; TME: Total mesorectal excision

Acknowledgements

The IMARI-study group consists of: J.D.W. van der Bilt, J.W.A. Burger, R.M.P.H. Crolla, F. Daams, I. Faneyte, M. Gerhards, E.J.R. de Graaf, W.J. de Jonge, W. van der Meij, S. J. Oosterling, L.P.S. Stassen, J.B. Tuynman, E.G.G. Verdaasdonk, H.L. van Westreenen, J.H.W. de Wilt.

Authors’ contributions

MDS, KT, SS, CPMH, SVD, CYP, CYN, ECJC, JHW, MAB, JPMD, GDM, WAB, PJT

and RH have made substantial contributions to the conception and design of his study and have been involved intensively in drafting and revising the manuscript. The IMARI-study group (JDWB, JWAB, PMPHC, FD, IF, MG, EJRG, WJJ, WM, SJC, LPS, JPT, EGGV, HLW and JHWW) has made substantial contri-butions to the conception and design of this study, in critically revising this manuscript and in organising and coordinating this study. All authors have read and approved this final version for publication.

Funding

The IMARI trial is an investigator initiated study funded by the Dutch Cancer Society (KWF) and third party funding by B. Braun Surgical, S. A and Stryker European Operations B.V. with no influence on protocol writing and no access to data.

Availability of data and materials

Data collection is in progress. When data collection and follow-up is finalized, data from the study will be available on reasonable request from the corre-sponding author.

Ethics approval and consent to participate

This study has been approved by the Medical Ethical Committee (METC 2019_055, August 14th, 2019) and Biobank committee (METC 2019_219, February 21st, 2020) of the Amsterdam UMC, location AMC. The protocol is registered by the Dutch Central Committee on Research Involving Human Subjects (NL67600.018.18). For all other participating centers approval of the local ethical committee and/or board of director will be obtained. Written informed consent will be obtained from all participants.

Consent for publication

Not applicable.

Competing interests

The authors declare there are no competing interests.

Author details

^1^Department of Surgery, Amsterdam UMC, Location AMC, Amsterdam, The Netherlands. ^2^Department of Gastroenterology, Amsterdam UMC, Location AMC, Amsterdam, The Netherlands. ^3^Department of Radiology, Amsterdam UMC, Location AMC, Amsterdam, The Netherlands. ^4^Department of Surgery, Meander Medical Center, Amersfoort, The Netherlands. ^5^Department of Surgery, Amphia Hospital, Breda, The Netherlands. ^6^Department of Paediatric Surgery, Emma Children’s Hospital, Amsterdam UMC, Location AMC, Amsterdam, The Netherlands.

Received: 22 September 2020 Accepted: 28 September 2020


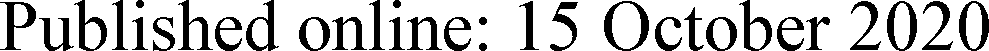


References

1. Borstlap WAA, Westerduin E, Aukema TS, Bemelman WA, Tanis PJ. Anastomotic leakage and chronic Presacral sinus formation after low anterior resection: results from a large cross-sectional study. Ann Surg. 2017; 266(5):870–7.
2. Yang J, Chen Q, Jindou L, Cheng Y. The influence of anastomotic leakage for rectal cancer oncologic outcome: a systematic review and meta-analysis. J Surg Oncol. 2020;121(8):1283–97.
3. Kverneng Hultberg D, Svensson J, Jutesten H, Rutegård J, Matthiessen P, Lydrup ML, et al. The impact of anastomotic leakage on long-term function after anterior resection for rectal Cancer. Dis Colon Rectum. 2020;63(5):619–28.
4. Ashraf SQ, Burns EM, Jani A, Altman S, Young JD, Cunningham C, et al. The economic impact of anastomotic leakage after anterior resections in English NHS hospitals: are we adequately remunerating them? Color Dis. 2013;15(4): e190–8.
5. Rahbari NN, Weitz J, Hohenberger W, Heald RJ, Moran B, Ulrich A, et al. Definition and grading of anastomotic leakage following anterior resection of the rectum: a proposal by the international study Group of Rectal Cancer. Surgery. 2010;147(3):339–51.
6. Boyce SA, Harris C, Stevenson A, Lumley J, Clark D. Management of low Colorectal Anastomotic Leakage in the laparoscopic era: more than a decade of experience. Dis Colon Rectum. 2017;60(8):807–14.
7. Penna M, Hompes R, Arnold S, Wynn G, Austin R, Warusavitarne J, et al. Incidence and risk factors for anastomotic failure in 1594 patients treated by Transanal Total Mesorectal excision: results from the international TaTME registry. Ann Surg. 2019;269(4):700–11.
8. Alverdy JC, Hyoju SK, Weigerinck M, Gilbert JA. The gut microbiome and the mechanism of surgical infection. Br J Surg. 2017;104(2):e14–23.
9. Foppa C, Ng SC, Montorsi M, Spinelli A. Anastomotic leak in colorectal cancer patients: new insights and perspectives. Eur J Surgical Oncol. 2020; 46(6):943–54.
10. Singh PP, Zeng IS, Srinivasa S, Lemanu DP, Connolly AB, Hill AG.

Systematic review and meta-analysis of use of serum C-reactive protein levels to predict anastomotic leak after colorectal surgery. Br J Surg.

2014;101(4):339–46.

1. Adamina M, Steffen T, Tarantino I, Beutner U, Schmied BM, Warschkow R. Meta-analysis of the predictive value of C-reactive protein for infectious complications in abdominal surgery. Br J Surg. 2015;102(6):590–8.
2. Challine A, Lefèvre JH, Creavin B, Benoit O, Chafai N, Debove C, et al. Can a local drainage salvage a failed colorectal or Coloanal anastomosis? A prospective cohort of 54 patients. Dis Colon Rectum. 2020;63(1):93–100.
3. Chen M, Song X, Chen LZ, Lin ZD, Zhang XL. Comparing mechanical bowel preparation with both Oral and systemic antibiotics versus mechanical bowel preparation and systemic antibiotics alone for the prevention of surgical site infection after elective colorectal surgery: a meta-analysis of randomized controlled clinical trials. Dis Colon Rectum. 2016;59(1):70–8.
4. McSorley ST, Steele CW, McMahon AJ. Meta-analysis of oral antibiotics, in combination with preoperative intravenous antibiotics and mechanical bowel preparation the day before surgery, compared with intravenous antibiotics and mechanical bowel preparation alone to reduce surgical-site infections in elective colorectal surgery. BJS Open. 2018;2(4):185–94.
5. Kiran RP, Murray AC, Chiuzan C, Estrada D, Forde K. Combined preoperative mechanical bowel preparation with oral antibiotics significantly reduces surgical site infection, anastomotic leak, and ileus after colorectal surgery. Ann Surg. 2015;262(3):416–25 discussion 23-5.
6. Abis GSA, Stockmann H, Bonjer HJ, van Veenendaal N, van Doorn-Schepens MLM, Budding AE, et al. Randomized clinical trial of selective decontamination of the digestive tract in elective colorectal cancer surgery (SELECT trial). Br J Surg. 2019;106(4):355–63.
7. Kye BH, Kim HJ, Kim HS, Kim JG, Cho HM. How much colonic redundancy could be obtained by splenic flexure mobilization in laparoscopic anterior or low anterior resection? Int J Med Sci. 2014;11(9):857–62.
8. Blanco-Colino R, Espin-Basany E. Intraoperative use of ICG fluorescence imaging to reduce the risk of anastomotic leakage in colorectal surgery: a systematic review and meta-analysis. Tech Coloproctol. 2018;22(1):15–23.
9. De Nardi P, Elmore U, Maggi G, Maggiore R, Boni L, Cassinotti E, et al. Intraoperative angiography with indocyanine green to assess anastomosis perfusion in patients undergoing laparoscopic colorectal resection: results of a multicenter randomized controlled trial. Surg Endosc. 2019;34(1):53–60.
10. Chan AW, Tetzlaff JM, Altman DG, Laupacis A, Gøtzsche PC, Krleža-Jerić K, et al. SPIRIT 2013 statement: defining standard protocol items for clinical trials. Ann Intern Med. 2013;158(3):200–7.
11. Chan AW, Tetzlaff JM, Gøtzsche PC, Altman DG, Mann H, Berlin JA, et al. SPIRIT 2013 explanation and elaboration: guidance for protocols of clinical trials. Bmj. 2013;346:e7586.
12. World Medical Association Declaration of Helsinki: ethical principles for medical research involving human subjects. Jama, 2013;310(20):2191–4.
13. D'Souza N, de Neree Tot Babberich MPM, d'Hoore A, Tiret E, Xynos E, RGH B-T, et al. Definition of the Rectum: An International, Expert-based Delphi Consensus. Ann Surg. 2019;270(6):955–9.
14. Mulder T, den Bergh MFQ K-v, de Smet A, van’t Veer NE, Roos D, Nikolakopoulos S, et al. Prevention of severe infectious complications after colorectal surgery using preoperative orally administered antibiotic prophylaxis (PreCaution): study protocol for a randomized controlled trial. Trials. 2018;19(1):51.
15. Moran BJ, Holm T, Brannagan G, Chave H, Quirke P, West N, et al. The English national low rectal cancer development programme: key messages and future perspectives. Colorectal Dis. 2014;16(3):173–8.
16. Roodbeen SX, Penna M, Mackenzie H, Kusters M, Slater A, Jones OM, et al. Transanal total mesorectal excision (TaTME) versus laparoscopic TME for MRI-defined low rectal cancer: a propensity score-matched analysis of oncological outcomes. Surg Endosc. 2019;33(8):2459–67.
17. van Koperen PJ, van Berge Henegouwen MI, Rosman C, Bakker CM, Heres P, Slors JF, et al. The Dutch multicenter experience of the endo-sponge treatment for anastomotic leakage after colorectal surgery. Surg Endosc. 2009;23(6):1379–83.
18. Gardenbroek TJ, Musters GD, Buskens CJ, Ponsioen CY, D'Haens GR, Dijkgraaf MG, et al. Early reconstruction of the leaking ileal pouch-anal anastomosis: a novel solution to an old problem. Color Dis. 2015;17(5): 426–32.
19. Talboom K, van Kesteren J, Sonneveld DJA, Tanis PJ, Bemelman WA, Hompes R. Early transanal closure after vacuum-assisted drainage for anastomotic leakage in rectal cancer surgery - a video vignette. Colorectal Dis. 2020;22(8):973–4.
20. Bastiaenen VP, Hovdenak Jakobsen I, Labianca R, Martling A, Morton DG, Primrose JN, et al. Consensus and controversies regarding follow-up after treatment with curative intent of nonmetastatic colorectal cancer: a synopsis of guidelines used in countries represented in the European Society of Coloproctology. Colorectal Dis. 2019;21(4):392–416.
21. Borstlap WAA, Musters GD, Stassen LPS, van Westreenen HL, Hess D, van Dieren S, et al. Vacuum-assisted early transanal closure of leaking low colorectal anastomoses: the CLEAN study. Surg Endosc. 2018;32(1):315–27.
22. Armstrong G, Croft J, Corrigan N, Brown JM, Goh V, Quirke P, et al. IntAct: intra-operative fluorescence angiography to prevent anastomotic leak in rectal cancer surgery: a randomized controlled trial. Colorectal Dis. 2018; 20(8):O226–o34.
23. Tulina IA, Zhurkovsky VI, Bredikhin MI, Tsugulya PB, Tsarkov PV. Selective approach for splenic flexure mobilization in total mesorectal excision followed by low colorectal anastomoses. Khirurgiia. 2018;7:41–6.
24. Mouw TJ, King C, Ashcraft JH, Valentino JD, DiPasco PJ, Al-Kasspooles M. Routine splenic flexure mobilization may increase compliance with pathological quality metrics in patients undergoing low anterior resection. Colorectal Dis. 2019;21(1):23–9.
25. Singal AG, Higgins PDR, Waljee AK. A primer on effectiveness and efficacy trials. Clin Transl Gastroenterol. 2014;5(1):e45.

Publisher’s Note

Springer Nature remains neutral with regard to jurisdictional claims in published maps and institutional affiliations.


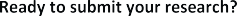

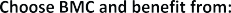

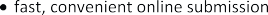

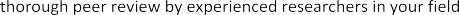

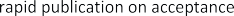

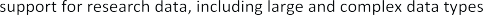

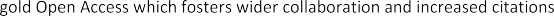

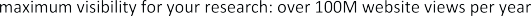

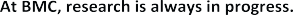

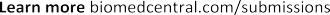

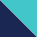


Multi-Interventional program for prevention and early Management of Anastomotic leakage after total mesorectal excision in Rectal cancer patIents,


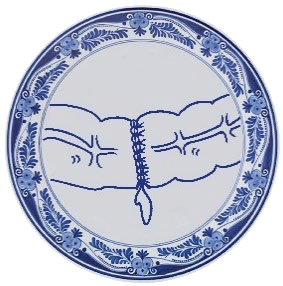


the IMARI-trial

M.D. Slooter^1^, S. Sharabiany^1^, G.D. Musters^1^, W.A. Bemelman^1^, P.J. Tanis^1^, R. Hompes^1^

1 Department of Surgery, Amsterdam UMC, Location AMC, Amsterdam, the Netherlands

**IMARI-trial Project group (Also see Appendix A ‘Organogram’):**

P.J. Tanis^1^ , R. Hompes^1^, W.A. Bemelman^1^, G.D. Musters^1^, M.D. Slooter^1^, M.A. Boermeester^1^, C.I.J. Ponsioen^2^, C.Y. Nio^3^, E.C.J. Consten^4^, J.H. Wijsman^5^

^1^ Department of Surgery, Amsterdam UMC, Location AMC, Amsterdam, the Netherlands.

^2^ Department of Gastro-enterology, Amsterdam UMC, Location AMC, Amsterdam, the Netherlands

^3^ Department of Radiology, Amsterdam UMC, Location AMC, Amsterdam, the Netherlands

^4^ Department of Surgery, Meander Medical Centre, Amersfoort, the Netherlands

^5^ Department of Surgery, Amphia Hospital, Breda, the Netherlands

## Participants of the IMARI-trial:

J.H. Wijsman^1^, J. Tuynman^2^, W. van der Meij^3^, P. Burger^4^, J. van der Bilt^5^, C. Sietses^6^, I. Faneyte^7^, H.L. van Westreenen^8^, E.G.G. Verdaasdonk^9^, L.P.S. Stassen^10^, E. Consten^11^, P. Steenvoorde^12^, M. Gerhards^13^, H. de Wilt^14^, S. J. Oosterling^15^, E.J.R. de Graaf^16^, B. van Vugt^17^, E.H.J. Belgers^18^, J. Heemskerk^19^

^1^ Department of Surgery, Amphia Hospital, Breda, the Netherlands

^2^ Department of Surgery, Amsterdam UMC, Location VUMC, Amsterdam, the Netherlands.

^3^ Department of Surgery, Bernhoven Hospital, Uden, Oss, the Netherlands

^4^ Department of Surgery, Catharina Hospital, Eindhoven, the Netherlands

^5^ Department of Surgery, Flevo Hospital, Almere, the Netherlands

^6^ Department of Surgery, Hospital Gelderse Vallei, Ede, the Netherlands

^7^ Department of Surgery, Hospital Group Twente, Almelo and Hengelo, the Neherlands

^8^ Department of Surgery, Isala Hospital, Zwolle, Meppel, Kampen, Steenwijk, Heerde, the Netherlands

^9^ Department of Surgery, Jeroen Bosch Hospital, Den Bosch, the Netherlands

^10^ Department of Surgery, Maastricht UMC, Maastricht, the Netherlands

^11^ Department of Surgery, Meander Medical Centre, Amersfoort, the Netherlands

^12^ Department of Surgery, Medical Spectrum Twente, Enschede, the Netherlands

^13^ Department of Surgery, Onze Lieve Vrouwe Gasthuis, Location East, Amsterdam, the Netherlands

^14^ Department of Surgery, Radboud UMC, Nijmegen, the Netherlands

^15^ Department of Surgery, Spaarne Gasthuis, Haarlem, Hoofddorp, the Netherlands

^16^ Department of Surgery, IJsselland Hospital, Capelle aan de IJssel, the Netherlands

^17^ Department of Surgery, Wilhelmina Ziekenhuis Assen, Assen, The Netherlands

^18^ Department of Surgery, Zuyderland Medical Center, Sittard-Geleen, The Netherlands

^19^ Department of Surgery, Laurentius Hospital Roermond, The Netherlands

**PROTOCOL TITLE:** Multi-Interventional program for prevention and early Management of Anastomotic leakage after total mesorectal excision in Rectal cancer patIents, the IMARI-trial

| **Protocol ID** | METC 2019_055 |
| --- | --- |
| **Short title** | IMARI-trial |
| **Version** | 6 |
| **Date** | 13-12-2022 |
| **Coordinating investigators**  **Principal investigator**  **Project leader & Principal Investigator** | **Drs. K.R. Wienholts**  *Address:* Amsterdam University Medical Centres, location AMC, Department of Surgery G4  Postbox 22660, 1100 DD Amsterdam, the Netherlands  *Email:* [k.r.wienholts@amsterdamumc.nl](mailto:k.r.wienholts@amsterdamumc.nl)  *Telephone:* +31 6 3115 6178  **Dr. R. Hompes**  *Address:* Amsterdam University Medical Centres, location AMC, Department of Surgery G4  Postbox 22660, 1100 DD Amsterdam, the Netherlands  *Email:* [r.hompes@amc.nl](mailto:r.hompes@amc.nl)  *Telephone:* +31 (0)20 566 29507  **Prof. dr. P.J. Tanis**  *Address:* Amsterdam University Medical Centres, location AMC, Department of Surgery G4  Postbox 22660, 1100 DD Amsterdam, the Netherlands  *Email:* [p.j.tanis@amc.nl](mailto:p.j.tanis@amc.nl)  *Telephone:* +31 629 06 82 75 |

| **Local principal investigators**   1. Prof dr. P.J. Tanis 2. Dr. J.H. Wijsman 3. Dr. J. Tuynman 4. Dr. W. van der Meij 5. Dr. P. Burger 6. Dr. J. van der Bilt 7. Dr. C. Sietses 8. Dr. I. Faneyte 9. Dr. E. van Westreenen 10. Dr. E. Verdaasdonk 11. Prof. dr. L.P.S. Stassen 12. Dr. E. Consten 13. Dr. P. Steenvoorde 14. Dr. M. Gerhards 15. Dr. H. de Wilt 16. Dr. S.J. Oosterling 17. Dr. E.J.R. de Graaf 18. Dr. B. van Vugt 19. E.H.J. Belgers 20. J. Heemskerk | 1. Amsterdam UMC, location AMC, Amsterdam 2. Amphia Hospital, Breda 3. Amsterdam UMC, location VUMC, Amsterdam 4. Bernhoven, Uden, Oss 5. Catharina Hospital, Eindhoven 6. Flevo Hospital, Almere 7. Hospital Gelderse Vallei, Ede 8. Hospital Group Twente 9. Isala, Zwolle, Meppel, Kampen, Steenwijk, Heerde 10. Jeroen Bosch Hospital, Den Bosch 11. Maastricht UMC, Maastricht 12. Meander Medical Center, Amersfoort 13. Medical Spectrum Twente, Enschede 14. OLVG, Amsterdam 15. Radboud UMC, Nijmegen 16. Spaarne Gasthuis 17. IJsselland ziekenhuis, Capelle aan de IJssel 18. Wilhelmina Ziekenhuis Assen 19. Zuyderland Medical Center 20. Laurentius Hospital Roermond   Also see Appendix A ‘Organogram’ and document I2 ‘Lijst deelnemende centra’. |
| --- | --- |
| **Sponsor** | Amsterdam University Medical Centres, location AMC Postbox 22660, 1100 DD Amsterdam, the Netherlands |
| **Subsidising party** | Dutch Cancer Society (KWF), Stryker European Operations B.V.  B. Braun Surgical, S.A. |
| **Independent expert** | Prof. dr. M.G.H. Besselink, Hepato-Pancreato-Biliary surgeon *Address:* Amsterdam University Medical Centres, location AMC, department of surgery G4  Postbox 22660, 1100 DD Amsterdam, the Netherlands |

# TABLE OF CONTENTS

1. INTRODUCTION AND RATIONALE 9
2. OBJECTIVES 14
3. STUDY DESIGN 15
4. STUDY POPULATION 16
   1. Population (base) 16
   2. Inclusion criteria 16
   3. Exclusion criteria 16
   4. Sample size calculation 16
5. TREATMENT OF SUBJECTS 17
   1. Standard care of the control arm 17
   2. Investigational treatment 17
6. METHODS 21
   1. Study parameters/endpoints 21
      1. Main study parameter/endpoint 21
      2. Secondary study parameters/endpoints 21
      3. Other study parameters 21
   2. Study procedures 22
   3. Withdrawal of individual subjects 23
   4. Follow-up of subjects withdrawn from treatment 23
   5. Premature termination of the study 23
7. SAFETY REPORTING 24
   1. Temporary halt for reasons of subject safety 24
   2. AEs and SAEs 24
      1. Adverse events (AEs) 24
      2. Serious adverse events (SAEs) 24
   3. Recording procedures for AE’s 25
   4. Reporting procedures for SAEs 25
   5. Follow-up of adverse events 25
   6. Data Safety Monitoring Board (DSMB) 25
8. STATISTICAL ANALYSIS 26
   1. Primary study parameter(s) 26
   2. Secondary study parameter(s) 26
9. ETHICAL CONSIDERATIONS 27
   1. Regulation statement 27
   2. Recruitment and consent 27
   3. Objection by minors or incapacitated subjects (if applicable) 27
   4. Benefits and risks assessment, group relatedness 27
   5. Compensation for injury 28
   6. Incentives (if applicable) 28
10. ADMINISTRATIVE ASPECTS, MONITORING AND PUBLICATION 29
    1. Handling and storage of data and documents 29
    2. Monitoring and Quality Assurance 29
    3. Amendments 30
    4. Annual progress report 30
    5. Temporary halt and (prematurely) end of study report 31
    6. Public disclosure and publication policy 31
11. REFERENCES 32

# LIST OF ABBREVIATIONS AND RELEVANT DEFINITIONS

| ABR | General Assessment and Registration form (ABR form), the application form that is  required for submission to the accredited Ethics Committee; in Dutch: Algemeen Beoordelings- en Registratieformulier (ABR-formulier) |
| --- | --- |
| AE  AL | Adverse Event  Anastomotic Leakage/ anastomotic leak |
| AR | Adverse Reaction |
| CA | Competent Authority |
| CCMO | Central Committee on Research Involving Human Subjects; in Dutch: Centrale  Commissie Mensgebonden Onderzoek |
| CRF  CRP CV | Case Record Form  C-reactive protein Curriculum Vitae |
| DSMB | Data Safety Monitoring Board |
| ERAS  EU | Enhanced Recovery After Surgery  European Union |
| EudraCT  EVAC FA | European drug regulatory affairs Clinical Trials  Endoscopic VACuum-assisted drainage Fluorescence Angiography |
| GCP | Good Clinical Practice |
| GDPR | General Data Protection Regulation; in Dutch: Algemene Verordening  Gegevensbescherming (AVG) |
| IB | Investigator’s Brochure |
| IBD IC  ICG | Inflammatory Bowel Disease Informed Consent  Indocyanine Green |
| LAR  IMP | Low Anterior Resection  Investigational Medicinal Product |

| IMPD | Investigational Medicinal Product Dossier |
| --- | --- |
| METC  MBP | Medical research ethics committee (MREC); in Dutch: medisch-ethische toetsingscommissie (METC)  Mechanical Bowel Preparation |
| PIF (S)AE SDD  SFM | Patient Information Sheet (Serious) Adverse Event Selective Bowel Preparation  Splenic Flexure Mobilization |
| SPC | Summary of Product Characteristics; in Dutch: officiële productinformatie IB1-tekst |
| Sponsor | The sponsor is the party that commissions the organisation or performance of the research, for example a pharmaceutical  company, academic hospital, scientific organisation or investigator. A party that provides  funding for a study but does not commission it is not regarded as the sponsor, but referred to as a subsidising party. |
| SUSAR  TME | Suspected Unexpected Serious Adverse Reaction  Total Mesorectal Excision |
| UAVG | Dutch Act on Implementation of the General Data Protection Regulation; in Dutch:  Uitvoeringswet AVG |
| WMO | Medical Research Involving Human Subjects Act; in Dutch: Wet Medisch-  wetenschappelijk Onderzoek met Mensen |

**SUMMARY**

**Rationale:** Anastomotic leakage (AL) is one of the most feared complications after rectal cancer surgery. AL leads to a significant increase of postoperative morbidity, long-term surgical complications, negative impact on quality of life, higher permanent stoma rates and impaired oncological outcome. Our research group recently published a cross-sectional study of outcomes after rectal cancer surgery in the Netherlands with a long-term incidence of AL of 20%. The current management of AL usually involves a deviating ileostomy, if not performed primarily, in combination with “passive” drainage of the abscess cavity via transanal or transcutaneous route. The cross-sectional study showed that almost half of the leaks do not heal and may require major salvage surgery.

Numerous risk factors have been identified for AL. Modifiable surgical factors include tension on the anastomosis and anastomotic perfusion. A more recently described pathophysiological mechanism relates to the intestinal microbiome. Given the multifactorial etiology, a multi-interventional program is required for the prevention of AL.

Mechanical bowel preparation (MBP) with oral antibiotics can lead to a reduction in AL by reduction of the fecal bulk and bacterial load. Splenic flexure mobilization (SFM) optimizes a tension-free anastomosis, particularly for the most distal rectal cancers. Intraoperative real-time fluorescence angiography (FA) using indocyanine green (ICG) assesses perfusion, thereby enabling precise delineation of bowel transection and final anastomotic vitality. Routine use of this technology has been associated with reduced AL rates.

If AL occurs, early diagnosis and “active” treatment allows for optimal control of pelvic sepsis, anastomotic healing and stoma reversal. No international consensus exists on a diagnostic pathway for early detection of AL, even though evidence is building for the use of C-reactive protein (CRP) in the early postoperative period. Considering “active” treatment our research group investigated the impact of endoscopic vacuum-assisted drainage (EVAC) of the abscess cavity in combination with early transanal closure of the anastomotic defect.

In the IMARI-trial we want to address all the interventions mentioned above within existing institutional enhanced recovery programs and prehabilitation initiatives (i.e. correction of anemia, optimization of nutritional status, cessation of smoking).

**Objective**: To increase the one year anastomotic integrity rate in patients undergoing total mesorectal excision (TME) for rectal cancer by the routine and quality controlled implementation of a multi-interventional program, which includes:

1. MBP with oral antibiotics
2. Tailored full SFM
3. Intraoperative FA using ICG
4. Routine CRP-measurement at day three postoperatively, CT-scan with rectal contrast on indication
5. EVAC with early transanal closure of the anastomotic defect

**Study design:** This is a multicenter prospective clinical effectiveness trial, whereby current local practice (control cohort) will be evaluated, and subsequently compared to the results after implementation of the multi-interventional program (intervention cohort). First, the control cohort will finish accrual. After finishing accrual of the control cohort, the full multi-interventional program will be implemented and checked for quality over a three month period in all participating hospitals, followed by accrual in the intervention cohort. Anastomotic integrity at one year will be determined by a CT-scan in all included patients.

**Study population:** Patients with primary rectal cancer and scheduled for a TME with planned restoration of bowel continuity, including patients for completion TME after previous local excision or regrowth in a watch and wait protocol.

**Intervention**: In the intervention cohort all perioperative measures will be implemented, described under ‘Objective’.

**Main study parameters/endpoints:** The primary endpoint of the IMARI-trial is anastomotic integrity at one year postoperative. The most important secondary aim is to determine the impact on the incidence of AL within 30 and 90 days and one year post-operation. Other outcomes include quality of life, protocol compliance, changes in rectal microbiome, FA details and other postoperative outcomes.

**Nature and extent of the burden and risks associated with participation, benefit and group relatedness:** Patients are asked to fill in questionnaires before surgery, 90 days and 1 year after the operation. Furthermore, stool samples will be taken for microbiota-analysis preoperative and 4 days post-operative. In the intervention cohort, a blood sample will be taken on day 3. When patients develop AL, an additional swab will be taken from the presacral cavity.

At one year a CT-scan will be performed to assess the primary endpoint. According to the guideline synopsis of non-metastatic colorectal cancer, a routine CT-scan at one year should be performed for follow-up.

Additional samples will be collected in the Amsterdam UMC locations and OLVG: drain fluid will be collected on day 1 postoperative (when a postoperative drain is placed during surgery) and each subsequent day until the drain is removed. Drain fluid is collected to asses if biomarkers are present in peritoneal drain fluid that can predict AL. Blood samples will be taken peri-operatively and on day 3

after surgery (in both cohorts). Blood samples are collected to asses if biomarkers are present for AL and whether the relative difference between the two measurements has a predictive value for AL.

# INTRODUCTION AND RATIONALE

## Problem

Colorectal cancer is one of the most common types of cancer worldwide, with the incidence still increasing. In the Netherlands about 2900 patients are being treated for rectal cancer each year. Oncological outcomes have improved since the introduction of pre-operative radiotherapy and the optimized radical resections, but this comes at a cost of treatment-induced morbidity and mortality.

The most dreaded complication after rectal cancer surgery is anastomotic leakage (AL), and occurs in up to 20% of the patients(1-3). AL leads to a significant increase of postoperative morbidity, long-term surgical complications, negative impact on quality of life, higher permanent stoma rates and impaired oncological outcome(4). The current management of AL usually involves a deviating ileostomy, if not performed primarily, in combination with “passive” drainage of the abscess cavity via transanal or transcutaneous route. Our research group recently published a cross-sectional study of outcomes after rectal cancer surgery, and the study showed that almost half of the leaks do not heal and may require major salvage surgery(2).

## Solution

Prevention and treatment of AL is necessary to improve patient cancer outcomes and quality of life.

Numerous risk factors have been identified for AL(3). Modifiable surgical factors include tension on the anastomosis and anastomotic perfusion. A more recently described pathophysiological mechanism relates to the intestinal microbiome(5-7). Several studies show that the microbiome potentially effects cancer recurrence and metastatic disease(6-8). As such mechanic bowel preparation (MBP) with oral antibiotics can lead to a reduction in AL by reduction of the fecal bulk and bacterial load(9), and might also improve oncological outcomes. Splenic flexure mobilization (SFM) optimizes a tension-free anastomosis, particularly for the most distal rectal cancers. Intraoperative real-time fluorescence angiography (FA) using indocyanine green (ICG) assesses perfusion, thereby enabling precise delineation of bowel transection and final anastomotic vitality. Routine use of this technology has been associated with reduced AL rates(10).

Considering “active” treatment our research group investigated the impact of endoscopic vacuum-assisted drainage (EVAC) of the abscess cavity in combination with early transanal closure of the anastomotic defect(11).

In the IMARI-trial we want to address all the interventions mentioned above within existing institutional enhanced recovery programs and prehabilitation initiatives (i.e. correction of anemia, optimization nutritional status, cessation of smoking).

## Summary of literature

*Mechanical bowel preparation with oral antibiotics*

Surgical site infections (SSI), including intra-abdominal abscesses, might be associated with intestinal faecal bulk and bacterial load. Reducing only the faecal bulk with only MBP has shown to have no effect on SSI. However, when MBP is combined with oral antibiotics, a significant reduction in AL has been described in colorectal cancer and inflammatory bowel disease patients(9, 12-17) while only oral antibiotics has a more limited effect (OR 0.70, 0.55–0.88 vs OR 0.47, 0.42–0.53)(15). The most recent meta-analysis of MBP with oral antibiotics revealed that preoperative antibiotics were associated with lower AL rates after elective colorectal procedures (OR 0.59, 0.53-0.67; p<0.001)(12). As a result, MBP combined with preoperative oral antibiotics results in a reduction in hospital stay and an earlier return to work(12, 15, 17). Currently, 75% of the colorectal surgeons in Europe prescribe MBP for colon surgery and in 95% for rectal surgery, and 11% of the colorectal surgeons combine it with preoperative oral antibiotics(18). Therefore, the introduction of the combination of MBP with preoperative oral antibiotics in all participating hospitals may lead to a reduction of AL.

*Splenic flexure mobilization*

A tension-free anastomosis is crucial for anastomotic healing, and anatomical studies prove that SFM is required for adequate mobilization of the afferent colonic conduit. Particularly if the sigmoid colon is resected with an anastomosis at the level of the pelvic floor, SFM is mandatory to obtain sufficient length(19, 20). Additional full SFM can offer approximately 30cm of additional length, and can be combined with either a low or high-tie on the inferior mesenteric artery(19, 21, 22). SFM during laparoscopic low anterior resections (LAR) is a safe and feasible option(21, 23), and seems advantageous as it avoids tension-related morbidity, does not excessively prolongs operation time, and leads to wider oncologic resection(21, 24).

*Perfusion assessment*

Intraoperative FA using ICG can visualize perfusion of the bowel selected for anastomotic reconstruction, which in turn aids the surgeon’s decision making on the anastomotic site(25). FA using ICG relies on a camera able to excite and detect the emission of ICG in the near infrared fluorescence spectrum. After intravenous administration, ICG rapidly binds to plasma proteins and is transported intravascular with minimal leakage to the interstitium, making ICG an ideal marker for perfusion. ICG is registered for in human use in numerous European countries, including the Netherlands, and is safe

to use as toxicity and allergic reactions rarely occur (1:10,000, as reported by manufacturer)(26). FA has already been described to be safe and readily achievable of perfusion assessment in colorectal surgery(10, 27, 28). A recent meta-analysis, including 1302 patients, showed low AL rates when FA was applied, especially in rectal cancer surgery (ICG 1.1% vs non-ICG 6.1%; p=0.02, OR 0.34, 0.16-0.74; p=0.006))(10). Therefore, introducing FA in all participating hospitals may lead to a reduction of AL.

*Early detection of AL*

Currently, no standard diagnostic examination is being performed for the early detection of AL. Although some leaks present early after surgery with a fulminant onset of sepsis, most leaks become clinically evident 8-12 days postoperatively. A meta-analysis by Singh et al showed that the value of CRP measured at day 3-5 postoperatively after colorectal surgery is a useful negative predictive test(29). This is in line with other studies(30, 31). The derived CRP cut-off values by Singh et al were 172 mg/l on day 3, 124 mg/l on day 4 and 144 on day 5 post-operation. CRP at these time points had high negative predictive value (97%), and low positive predictive value (21-23%) for AL. An earlier time point is more useful clinically as it allows an earlier assessment for leakage and initiation of appropriate management.

## Results of own research

*EVAC treatment*

AL after low pelvic anastomosis has various treatment options, but are not always successful. The abscess is most commonly treated by “passive” drainage either percutaneously or transanally. In a recently published Dutch nationwide study, our research group showed an anastomotic leak rate of 20%. We found that almost half of the leaks, which are treated with current “passive” management, do not heal and may require major salvage surgery(2). This means that 10% of the patients had a chronic presacral sinus after one year. Also another study found that half of the leaks might not heal with this conventional treatment(32).

Management of the chronic sinus means major surgery taking down the leaking anastomosis followed by either redo anastomosis or intersphincteric proctectomy with omentoplasty and permanent colostomy(33). EVAC treatment seems to be a valuable alternative treatment option(34). By changing the EVAC-sponge two times per week and tapering the size of the EVAC-sponge systematically, the abscess cavity gradually collapses. This technique is labor-intensive, expensive and takes several weeks until closure is achieved(34). Against all surgical principles, it was hypothesized that an anastomotic defect could be closed transanally after the presacral abscess cavity was cleaned using a short course of EVAC treatment. This technique seems to be successful in patients with AL after ileal pouch-anal anastomosis for ulcerative colitis and familial adenomatous polyposis in comparison to conventional treatment(35). Secondary anastomotic healing was achieved in all patients in the early

surgical closure group, which was significantly higher compared to 52% in the conventional treatment group, without a significant difference in direct medical costs. Although promising, more research was needed to evaluate if this success rate can also be achieved in rectal cancer patients who underwent low anterior resection, especially after neo-adjuvant radiotherapy. To answer this question, we performed a prospective cohort study including 30 patients and showed that anastomotic healing was achieved in 79% of the patients at 12 months, with a direct medical cost of only €8933,-(11). In our research we show that EVAC treatment with transanally closure of the defect seems to result in an earlier and more successful closure of the anastomotic defect, without increasing direct medical costs. Therefore, EVAC deserves to be included in the current treatment strategy of AL.

*Perfusion assessment*

Adequate blood supply of the anastomosis is one of the key factors to warrant anastomotic integrity. The current strategy to evaluate the anastomotic perfusion is by visual assessment and palpation of pulsating vessels. Near infrared imaging for perfusion assessment is reported to aid the surgeon in the decision making on the site of the anastomosis. The principal investigator of our research group has been involved in research investigating the additive value of FA using ICG. First a feasibility study showed that perfusion angiography of colorectal anastomosis at the time of their laparoscopic construction is feasible and readily achievable with minimal added intraoperative time(36, 37).

Thorough research in literature encouraged the use of FA using ICG as it holds great potential for intraoperative guidance(38). These promising results were reason for the execution of a prospective multi-centre phase II trial recruiting 504 patients(27). In the study FA resulted in a change in the site of bowel division in 5.8% with no subsequent leaks in these patients. The study showed the overall leak rate for colorectal operations not involving FA 5.8%, compared to 2.6% with use of FA (p = 0.009). For LARs alone, the leak rates were 10.7% (39 of 365) versus 3% (3 of 90) (p = 0.031). In conclusion, the study showed that FA can be used to assess intestinal vascularity before and after anastomosis, and that use of FA leads to a significant reduction of AL in LAR and overall colorectal operations.

This is in concordance with literature as a recent meta-analysis, including 1302 patients, showed low AL rates when FA was applied, especially in rectal cancer surgery (ICG 1.1% vs non-ICG 6.1%; p=0.02)(10). The multicentre phase II trial by Ris et al was published after the execution of the systematic review and meta-analysis, and thus was not concluded. Currently our research group is participating in an international RCT to investigate the effect of FA on anastomotic leakage.

*Surgical site infection*

One of the members of our project group is currently member of the WHO Guidelines Development Group. Recently this group published a WHO recommendation on preoperative measures for SSI prevention(39). On the basis of systematic literature reviews and expert consensus they presented 13

recommendations, including MBP and the use of oral antibiotics. Meta-analysis showed that preoperative MBP combined with oral antibiotics reduces SSI compared with MBP alone (combined OR 0.56, 0.37-0.83). They concluded that preoperative oral antibiotics should be used in combination with MBP in adult patients undergoing elective colorectal surgery to reduce the risk of SSI conditional recommendation, moderate quality of evidence).

## Rationale for the IMARI-study

The IMARI-trial addresses a relevant, frequently occurring, and unresolved clinical problem after rectal cancer surgery. Patients diagnosed with AL often suffer from a complicated, protracted postoperative course, including ICU stay, (non-)surgical reinterventions, resulting in significant physical and psychological distress. Even after initial recovery, a subgroup of patient will develop chronic pelvic infectious complications with a high permanent stoma rate. This heavily affects quality of life, and increases the risk of local recurrence and decreases survival rates. By increasing the chance of long-term anastomotic integrity, the IMARI-trial contributes to more cure and better quality of life.

# OBJECTIVES

The primary objective of the IMARI-trial is to increase the one year anastomotic integrity rate in patients undergoing total mesorectal excision (TME) for rectal cancer by the routine and quality controlled implementation of a multi-interventional program, which includes:

1. MBP with oral antibiotics
2. Tailored full SFM
3. Intraoperative FA using ICG
4. Routine CRP-measurement at day three postoperatively, CT-scan with rectal contrast on indication
5. EVAC with early transanal closure of the anastomotic defect

# STUDY DESIGN

The IMARI-trial is a multicenter prospective clinical effectiveness trial, whereby current local practice (control cohort) will be evaluated, and subsequently compared to the results after implementation of the multi-interventional program (intervention cohort). All participating hospitals will recruit patients for the control cohort. After finishing accrual of the control cohort (N=244), the full multi-interventional program will be implemented in all participating hospitals and checked for quality over a three month period, followed by accrual in the intervention cohort (N=244).

The trial will be carried out in 17 hospitals in the Netherlands. Anastomotic integrity at one year will be determined by a CT-scan in all included patients.

The design of this trial can also be found in the Appendix B ‘Trial design’.

# STUDY POPULATION

## Population (base)

Patients with primary rectal cancer and scheduled for a TME with planned restoration of bowel continuity.

## Inclusion criteria

In order to be eligible to participate in this study, a subject must meet all of the following criteria:

1. Patients with a diagnosis of primary rectal cancer with the lower border below the level of the sigmoid take-off on MRI, or regrowth in a watch and wait protocol, or undergoing completion/salvage surgery after local excision;
2. Age above 18;
3. Able to fill in questionnaires in Dutch and to come to out-patient-clinic visits;
4. Written informed consent.

## Exclusion criteria

A potential subject who meets any of the following criteria will be excluded from participation in this study:

1. Patients not undergoing resection with colo-rectal/anal anastomosis;
2. Local recurrent rectal cancer;
3. Locally advanced rectal cancer requiring extended or multi-visceral excision;
4. Synchronous colonic resections;

## Sample size calculation

In a Dutch nationwide study, the AL rate was 20%, with anastomotic integrity of 90% after one year. Meta-analysis of MBP with oral antibiotics revealed that preoperative antibiotics were associated with lower AL rates (OR 0.59, 0.53-0.67; p<0.001)(12). Pooled analysis of studies using routine FA showed an OR of 0.34 (0.16-0.74;p=0.006)(10). Together with full SFM, the estimated reduction in AL rate is 50%. In the CLEAN-study, treatment with EVAC and early surgical closure resulted in anastomotic healing in two thirds of the patients(11). Therefore, we hypothesized that the combination of all interventions will increase the anastomotic integrity rate from 90% to 97% at one year.

Applying a Chi-square test with a two-sided 0.05 significance level and 80% power, and with an estimated drop-out of 10%, a total number of 488 patients (244 per cohort) are needed to be able to detect a 7% increase in anastomotic integrity by insertion of the combined interventions.

# TREATMENT OF SUBJECTS

## Standard care of the control arm

Patients will receive standard care in all participating hospitals for the control cohort. Patients will be asked to fill in questionnaires at inclusion, 90 days and one year after surgery. Microbiota-analysis will be performed by taking stool samples preoperatively (before start of any MBP or preoperative antibiotics, if part of local protocol) and at day 4. When a patient is discharged before day 4, the sec-ond stool sample will be collected on the day of discharge. During surgery a swab will be taken from the anastomotic site and the donut from the operation will also be used. In the intervention cohort, a blood sample will be taken on day 3. When patients develop an AL, an additional swab of the presa-cral cavity will be taken.

Additional samples will be collected in the Amsterdam UMC locations and OLVG: drain fluid will be collected on day 1 postoperative (when a postoperative drain is placed during surgery) and each subsequent day until the drain is removed. Blood samples will be taken peri-operatively and on day 3 after surgery (in both cohorts). Drain fluid is collected to asses if biomarkers are present in peritoneal drain fluid that can predict AL. Blood samples are collected to asses if biomarkers are present for AL and whether the relative difference between the two measurements has a predictive value for AL.

Patients will be followed for one year, with a CT-scan at one year to meet the primary endpoint.

## Investigational treatment

In the IMARI-trial the multi-interventional program will be implemented within existing institutional enhanced recovery programs and prehabilitation initiatives (i.e. correction of anemia, optimization of nutritional status, cessation of smoking). The trial interventions of the multi-interventional program will be discussed below.

*Mechanical bowel preparation with oral antibiotics*

MBP will be achieved the day before surgery by oral administration of 2 litres of polyethylene glycol (movi-prep) or sodium phosphate.

One of the following two antibiotic schemes will be implemented (40, 41) (also according to unpublished work from the SELECT(42), pre-caution trial(43) and Amsterdam UMC clinical protocol):

1. 10 millilitres of selective digestive decontamination (SDD) solution should be administered four times daily during the three days prior to surgery; every 10 millilitres contains: colistine 100 mg, tobramycine 80 mg, and amphotericine B 500 mg
2. 10 millilitres of SDD solution should be administered three times daily during three days prior to surgery; every 10 millilitres contains: colistine 100 mg, tobramycine 80 mg, and nystatin 2000000IU.

Microbiota-analysis:

Microbiota-analysis will be performed by taking stool samples preoperatively (before start MBP or antibiotics) and 4 days after surgery. During surgery the spare donut of the resection (that is not submitted for pathological evaluation) and a swab from the anastomotic site will be submitted for microbiota-analysis. When patients develop AL, an additional swab from the presacral cavity will be taken. In the intervention cohort, a blood sample will be taken on day 3. When patients develop an AL, an additional swab of the presacral cavity will be taken.

Additional samples will be collected in the Amsterdam UMC locations and OLVG: drain fluid will be collected on day 1 postoperative (when a postoperative drain is placed during surgery) and each subsequent day until the drain is removed. Blood samples will be taken peri-operatively and on day 3 after surgery (in both cohorts).

Drain fluid is used to asses if biomarkers are present in peritoneal drain fluid that can predict AL. Blood samples are collected to asses if biomarkers are present for AL and whether the relative difference between the two measurements has a predictive value for AL.

Samples will be stored at the Tytgat Institute in Amsterdam UMC, location AMC. Microbiota profiling will be done in the Amsterdam UMC, location AMC, using an Illumina Miseq platform. In addition, we intend to perform metatranscriptomics on selected samples to look for presence and activity of collagenolytic Enterococcus faecalis and additional detrimental species for anastomotic integrity.

*Tailored full splenic flexure mobilization*

A full SFM will be routinely performed for low rectal cancer according to the LOREC definition for low rectal cancer(44, 45). In accordance with the LOREC definition of low rectal cancer, a tumor is considered low if the distal border is located distal to the point where the levator ani muscles insert on the pelvic bone on sagittal MRI(45). For all other (mid-)rectal cancers that will be treated by TME, full SFM can be considered to create a tension-free anastomosis.

Surgical procedure:

Before or after ligation of the inferior mesenteric artery (low or high tie, according to the surgeon’s preference) the splenic flexure is fully mobilized. This can be done either from medial to lateral or lateral to medial. For a full SFM the inferior mesenteric vein requires to be divided at the lower border of the pancreas just lateral to the angle of Treitz. Furthermore the mesentery of the distal transverse

colon needs to be completely released from the body and tail of the pancreas with full release of the omentum from the distal transverse colon.

*Intraoperative FA using ICG*

Intraoperative FA using ICG will be performed in all patients to assess perfusion prior to division of the bowel at the planned proximal transection point and after anastomotic construction. ICG will be administered intravenously at least once during the operation for perfusion assessment using nearinfrared laparoscopy. The specifics of each operation, including the decision to make a change to the planned anastomosis following FA assessment, will be at the discretion of the operating surgeon. After mobilization of the rectum, an intracorporeal or extracorporeal assessment technique can be used. The method used will be captured on the intraoperative CRF. First, the proximal colon will be assessed under white light and the point of planned transection marked. For extracorporeal methods, the white light (WL) assessment can be performed under direct vision without the use of the laparoscope if preferred. Additional aides to perfusion assessment, such as evaluation of the marginal artery supply, are allowed during WL assessment. A bolus of 0.1mg/kg of 2.5-5mg/ml ICG (reconstituted as per the manufacturer’s instructions) will be administered intravenously via a peripherally sited cannula.

Proximal transection assessment:

- Intracorporeal: the colonic and rectal stump perfusion will be assessed using near infrared laparoscopy (e.g. Novadaq PINPOINT - laparoscopic surgery; Firefly – robotic surgery etc.). The maximum intensity of fluorescence in the proximal colon and rectal stump will be assessed subjectively as “clearly fluorescent”, “borderline fluorescence”, or “no fluorescence”. Any change in the planned transection level or revision of the rectal stump as a result of FA assessment will be recorded.
- Extracorporeal; with the exteriorized bowel only FA assessment of the proximal bowel is possible. The maximum intensity of fluorescence in the proximal colon will be assessed subjectively as “clearly fluorescent”, “borderline fluorescence”, or “no fluorescence”. Any change in the planned transection level as a result of FA assessment will be recorded.

Anastomosis assessment:

Colo-anal anastomosis will be performed according to surgeon’s preference (hand-sewn, stapled, end-to-end, end-to-side, colo-pouch etc.). This may be followed by assessment of anastomotic perfusion after a second bolus of 0.1mg/kg of ICG administered via a peripheral cannula by discretion of the surgeon, but this is not obliged. The intensity of fluorescence in the proximal colon and rectal stump will be subjectively recorded as “clear fluorescence”, “borderline fluorescence”, or “no fluorescence”.

Any anastomotic revision will be recorded. Use of a deviating stoma will be at the discretion of the surgeon, with the reason for deviation and the relation to FA assessment will be documented.

A third dose of ICG is allowed as preferred by the operating surgeon with, the dose and timing recorded on the CRF.

*Routine CRP-measurement at day three postoperatively and CT-scan with rectal contrast on indication*

In the multi-interventional program the CRP measurement will be performed on day three postoperatively. A cut-off value of 172 mg/l will be maintained. When the value rises above 172 mg/l and there is a clinical suspicion for AL, a CT-scan with rectal contrast will be performed. When there is no clinical suspicion, CRP measurement will be repeated at day four postoperatively. When the value is stable or higher, a CT-scan with rectal contrast will be performed to exclude AL. For the flow diagram see Appendix C ‘Post-operative management algorithm’.

*EVAC with early transanal closure of the anastomotic defect*

If the CT-scan with rectal contrast reveals a leak and/or presacral abscess, all participating centers will contact the initiating center (Amsterdam UMC) for consultation and a deviating ileostomy will be constructed, if not created primarily. Transanal endoscopy will evaluate the characteristics of AL (ischemia, significant retraction of the afferent colon, extent of dehiscence and/or anastomotic fistula). If the cavity is suitable, EVAC treatment will start and endo-sponges will be placed. Every four days the AL and cavity will be evaluated by the gastroenterologist and surgeon, and if necessary new sponges will be placed. When the cavity is clean and there is no/minimal retraction of the afferent loop, the cavity will be closed transanally and re-evaluated after 2 weeks by endoscopy.

If the first endoscopic evaluation shows ischemia, significant retraction of the afferent colon, and extent of dehiscence, a different pathway will be followed. Either an early or late re-do of the anastomosis, with closure of the deviating stoma on the long-term, or a permanent colostomy with intersphincteric proctectomy and omentoplasty will be performed. For more information, see Appendix D ‘Pro-active management algorithm’.

# METHODS

## Study parameters/endpoints

- - 1. **Main study parameter/endpoint**

The primary endpoint of the IMARI-trial is anastomotic integrity at one year postoperative.

## Secondary study parameters/endpoints

The most important secondary aim is to determine the impact on the incidence of AL within 30 and 90 days and one year post-operation.

## Other study parameters

1. Quality of life (EQ-5D, QLQ-C30, QLQ-CR29), functional outcomes (LARS, UDI-6, IIQ-7, IIEF for male and MFSFQ for female),productivity losses and medical consumption (iPCQ, iMCQ) pre-op, 90 days post-op and one year after operation.
2. Protocol compliance to any intervention
3. Compliance in association to AL
4. Changes in rectal microbiome and correlation to AL
5. Change in management due to FA using ICG
   1. Site of proximal bowel division used for anastomosis
   2. Redo anastomosis or reinforcement of anastomosis after construction anastomosis
   3. Decision for diverting stoma
   4. Decision for Hartmann or abdominoperineal resection rather than restorative procedure
6. Diagnostic accuracy of CRP for AL
7. Efficacy of EVAC with early transanal closure of the anastomotic defect
8. Permanent stoma rate
9. Temporary stoma rate and stoma duration
10. Operative and post-operative complications within 30 days of operation (using the Clavien-Dindo classification of surgical complications)
11. Death
12. Hospital stay
13. Reintervention rate
14. Overall and stoma-related readmission
15. Local recurrence at one year post-operation
16. Cost analysis of AL and EVAC therapy

## Study procedures

The intervention group will receive care according to the ERAS protocol including the multi-interventional program, as extensively described in chapter 5.2. The multi-interventional program includes:

1. MBP with oral antibiotics
2. Tailored full SFM
3. Intraoperative FA using ICG
4. Routine CRP-measurement at day three postoperatively, CT-scan with rectal contrast on indication
5. EVAC with early transanal closure of the anastomotic defect

*Questionnaires*

To measure quality of life, several questionnaires will be used. Quality of life questionnaires will be collected through the data collection initiative of the Prospective Dutch ColoRectal Cancer (PLCRC) group (clinicaltrials.gov NCT02070146). Based on patient preference, these questionnaires will either be sent either to the patients’ home addresses, accompanied by a return envelope provided with postage stamps and the address of the hospital or sent digitally through a digital platform (Profiel). Patients will be asked to fill in questionnaires at inclusion and 90 days post-op and 1 year after surgery.

Patients will have to sign a separate informed consent form to participate in the PLCRC. If patients are not willing to participate in the PLCRC, but only want to participate in the IMARI-trial, questionnaires will be send by post (as described above) by the investigators.

The following questionnaires will be used:

EuroQol 5D (EQ-5D): This is a standardized instrument developed by the EuroQol Group as a measure of health-related quality of life.

Global quality of life (EORTC-QLQ-C30): This questionnaire contains the global quality of life dimension in cancer patients.

Global quality of life (EORTC-QLQ-CR29): This questionnaire is developed to assess the quality of life in colorectal patients.

Low Anterior Resection Syndrome score (LARS score): This questionnaire is designed to collect data on bowel dysfunction following a low anterior resection for rectal cancer.

Urogenital Distress Inventory (UDI-6) and Incontinence Impact Questionnaire (IIQ-7): These questionnaires are developed to assess urinary distress and incontinence symptoms in men and women, and coherent quality of life.

International Index of Erectile Function (IIEF): This questionnaire is developed to assess male sexual function.

McCoy Female Sexuality Questionnaire (MFSQ): This questionnaire is developed to assess female sexual function.

iMTA Productivity Cost Questionnaire (iPCQ): This questionnaire is developed to assess productivity losses for socio-economic evaluations.

iMTA Medical Consumption Questionnaire (iMCQ): This questionnaire is complementary to the iPCQ and is developed to assess medical consumption for socio-economic evaluations.

*Other outcomes*

Preoperative, during surgery, post-operative and when patients develop AL, samples will be collected for microbiota-analysis.

Patients will be followed for one year during routine outpatient clinic visits for surgical and oncological follow-up. At 12 months a CT-scan will be performed to meet the primary endpoint. The CT-scan at 12 months is standard of care(46).

For the flow diagram for patients see Appendix E ‘Schema te doorlopen stappen voor deelnemers’.

## Withdrawal of individual subjects

Subjects can leave the study at any time for any reason if they wish to do so without any consequences. The investigator can decide to withdraw a subject from the study for urgent medical reasons.

## Follow-up of subjects withdrawn from treatment

Patients whom have withdrawn from the study, but are still willing in participating in the follow-up will be followed according to the specifications of the patient.

## Premature termination of the study

Premature termination of the study is not expected.

Halfway through the accrual of the intervention group, an interim analysis will assess protocol com-pliance to the multi-interventional program. If protocol compliance is not fully achieved, three months of education and protocol training will follow, during which accrual will be continued.

# SAFETY REPORTING

## Temporary halt for reasons of subject safety

In accordance to section 10, subsection 4, of the WMO, the sponsor will suspend the study if there is sufficient ground that continuation of the study will jeopardize subject health or safety. The sponsor will notify the accredited METC without undue delay of a temporary halt including the reason for such an action. The study will be suspended pending a further positive decision by the accredited METC. The investigator will take care that all subjects are kept informed.

## AEs and SAEs

- - 1. **Adverse events (AEs)**

Adverse events are defined as any undesirable experience occurring to a subject during the study, whether or not considered related to the multi-interventional program. All adverse events reported spontaneously by the subject or observed by the investigator or his staff are recorded.

## Serious adverse events (SAEs)

A serious adverse event is any untoward medical occurrence or effect that

- results in death;
- is life threatening (at the time of the event);
- requires hospitalisation or prolongation of existing inpatients’ hospitalisation;
- results in persistent or significant disability or incapacity;
- is a congenital anomaly or birth defect; or
- any other important medical event that did not result in any of the outcomes listed above due to medical or surgical intervention but could have been based upon appropriate judgement by the investigator.

NOTE: The following situations do not need to be reported as SAEs:

- Any admission unrelated to an AE, e.g., for labour/delivery, cosmetic surgery, social and/or convenience admissions to a hospital.
- Elective hospitalisation (planned before the subject consented for study participation) for pre-existing conditions that did not exacerbate during the study period as judged by the clinical investigator and where admission did not take longer than anticipated.
- Admission for diagnosis or therapy of a condition that existed before the start of the study and has not increased in severity or frequency as judged by the clinical investigator.
- Protocol-specified admission, e.g., for a procedure required by the study protocol.
- Anticipated day-to-day fluctuations of pre-existing disease(s) or condition(s) present at the start of the study that do not worsen.

## Recording procedures for AE’s

All AE’s observed by the investigator or his staff, or reported by the subject, whether or not related to the investigational medicinal product, are recorded in the subject’s medical dossier and in the CRF. AEs need to be recorded till end of study within the Netherlands, as defined in the protocol.

## Reporting procedures for SAEs

In the control cohort, the investigator will not report SAE’s to the sponsor, since patients will receive standard care and any SAE’s occurring in the control cohort can reasonably be expected.

In the intervention cohort, the investigator will report all SAEs to the sponsor without undue delay after obtaining knowledge of the events. The sponsor will report the SAEs through the web portal *ToetsingOnline* to the accredited METC that approved the protocol, within 7 days of first knowledge for SAEs that result in death or are life threatening followed by a period of maximum of 8 days to complete the initial preliminary report. All other SAEs will be reported within a period of maximum 15 days after the sponsor has first knowledge of the serious adverse events.

SAEs in the intervention cohort need to be reported until 30 days after initial surgery.

## Follow-up of adverse events

All AEs are followed until they have abated, or until a stable situation has been reached. Depending on the event, follow up may require additional tests or medical procedures as indicated, and/or referral to the general physician or a medical specialist. AEs that are still ongoing at the end of the study period must be followed up to determine the final outcome.

## Data Safety Monitoring Board (DSMB)

This study is considered a low risk trial, in which patients in both study groups are subjected to operations that are already being performed in clinical practice in the Amsterdam UMC, location AMC. Therefore, no DSMB will be assigned.

# STATISTICAL ANALYSIS

## Primary study parameter(s)

The primary endpoint, anastomotic integrity, will be compared between the two trial cohorts (control and intervention cohort) using a two-sided Chi-square test with a significance level of 0.05. Statistical analyses will be performed using SPSS software for Windows version 25.

## Secondary study parameter(s)

The incidence of AL in each cohort will be summarized for the following time points: within 30 and 90 days post-operation, and one year postoperatively. The analysis will compare leak rates between the cohorts using generalized estimating equations model adjusting for the stratification factors. This approach will be used to test the two-sided hypothesis that the AL rate is equal in both cohorts (i.e. an odds ratio of 1), considering the 95% confidence interval and a p-value of 0.05.

Other secondary endpoints with binary measures (compliance to protocol, change in management due to FA, permanent or temporary stoma rate, complications, rate of re-intervention and death) will be analyzed using multi-variable logistic regression adjusting for the stratification factors.

Secondary endpoints with continuous measures – e.g. length of stay – will be analyzed using linear regression models adjusting for the stratification factors. When the data is not normally distributed, the data will be transformed to achieve normal distribution.

The secondary endpoint ‘duration of temporary stoma’ will be analyzed using a cox-regression model with adjusting for the stratification factors.

*Analysis of quality of life data*

Quality of life data will be graphically represented across all time points and analyzed according to the manuals and will presented as domain and summarized scores. Questionnaire outcome comparisons will be analyzed using linear mixed models.

# ETHICAL CONSIDERATIONS

## Regulation statement

This study will be conducted according to the principles of the Declaration of Helsinki (Fortaleza, Brasil, October 2013) and in accordance with the Medical Research Involving Human Subjects Act (WMO) and other guidelines, regulations and Acts.

## Recruitment and consent

Suitable patients will be approached for entry into the trial at the first outpatient visit at the surgery department after the diagnosis of rectal cancer has been made. The rationale for the trial is explained to the patient. A written patient information sheet is provided and patients will be given the opportunity to ask questions. In the control cohort, the willing patients are asked to sign the informed consent form at the first outpatient visit. If an additional reflection period is required, willing patients will be asked to sign the informed consent form at a later moment. In the intervention cohort, the willing patients are asked to sign the informed consent after a sufficient reflection period. Informed consent will be obtained before any trial intervention in both cohorts. Written informed consent is taken by surgeons, surgical registrars or trained research nurses. When consent has been obtained, the original form is kept in the trial file and a copy is given to the patient. Baseline data as well as baseline questionnaires are collected.

The patient information sheet (PIF) consists of two versions: one for the control cohort and one for the intervention cohort. The PIF for the control cohort will give general information to the patient and asks permission for the use of their data for research purposes. The PIF for the intervention and intervention registration cohort will extensively explain the multi-interventional program. We chose two different PIFs, because patients do not have a choice between the cohorts due to subsequent accrual of the control and intervention cohort.

## Objection by minors or incapacitated subjects (if applicable)

Minors and legally incompetent adults are excluded from the trial.

## Benefits and risks assessment, group relatedness

Patients included in the control cohort do not directly benefit from participation in this study nor will they be exposed to any risks or burden. Patients included in the intervention cohort might benefit from the implementation of the multi-interventional program in the current ERAS protocol. However, the study may generate further insight on the interpretation and support further implementation of the multi-interventional program, with the aim to decrease AL in future patients undergoing TME with anastomotic reconstruction.

## Compensation for injury

The sponsor/investigator has a liability insurance which is in accordance with article 7 of the WMO.

The sponsor (also) has an insurance which is in accordance with the legal requirements in the Netherlands (Article 7 WMO). This insurance provides cover for damage to research subjects through injury or death caused by the study.

The insurance applies to the damage that becomes apparent during the study or within 4 years after the end of the study.

## Incentives (if applicable)

Enrolled patients will not receive any special incentives, compensation or treatment through participation in this trial.

# ADMINISTRATIVE ASPECTS, MONITORING AND PUBLICATION

## Handling and storage of data and documents

Every included patient will be assigned a three digit study number. Communication occurs only with this number. The full name and birth date of the patient will only be recorded on the informed consent form.

A study coordinator coordinates the study, monitors patient inclusion and protocol steps, data collection, data entry, preparation and performs analyses and will report the data. Continuous data monitoring, and data collection on a CRF will guarantee complete and real-time prospective recording of data. Data will be collected and stored at the AMC in a separate, closed room.

The samples for the microbiota analysis will be stored in an Tytgat Institute freezer (-80), with full certification. The samples will be labelled with the three digit study number. The samples will be stored for 10 years after the end of the study and may be used for additional analyses concerning the role of microbiota on the mucosa in the future.

## Monitoring and Quality Assurance

*Monitoring*

The study will be monitored. Monitoring is requested at the Clinical Research Unit (see document ‘K5 Bevestiging aanvraag centrale CRU-monitoring IMARI trial’). The monitoring plan will be determined after the first intake for initiation of monitoring.

*Educational program*

To ensure quality of the multi-interventional program, an educational program prior to the start of the inclusion of intervention cohort will be organized. Educational videos will be provided on full SFM and EVAC with early transanal closure. Random checks of procedural videos will ensure

quality of the full flexure mobilization. Two workshops on EVAC with early transanal closure will be given for both surgeons and gastro-enterologists. EVAC and early transanal closure in the first two to five patients in every center will be guided on site by the surgeons of this project group. All EVAC procedures and early transanal closures will be recorded and checked for quality by the surgeons of this project group. A system for remote proctoring will ensure quality throughout the entire trial period.

*Description of work*

The execution of central data management will be performed by a PhD-student and a research nurse. In addition, the local data management will be performed by the local investigator and monitored by the research nurse. The continuous data monitoring and data collection based on high quality eCRFs

guarantees complete and timely recording, handling and storage of data and documents. The PhD-student and research nurse will also be responsible for collecting the quality of life questionnaires.

*Central Data management*

The central data manager will maintain quality of documentation by local data managers in the eCRF, and clarify mistakes where necessary. The central data manager develops the eCRFs, adds participating hospitals to the database, tests the database, and informs the local data managers about how to use the database. Furthermore, the central data manager keeps the Trial Master File

according to GCP guidelines. In case of uncertainties or questions in the eCRF, additional queries for the local data managers may be formulated by the central data manager.

*Local Data management*

Data is registered by the treating physician in the patient file, and registered in the eCRF by the local data manager.

## Amendments

Amendments are changes made to the research after a favourable opinion by the accredited METC has been given. All amendments will be notified to the METC that gave a favourable opinion.

All substantial amendments will be notified to the METC and to the competent authority.

Non-substantial amendments will not be notified to the accredited METC and the competent authority, but will be recorded and filed by the sponsor.

A ‘substantial amendment’ is defined as an amendment to the terms of the METC application, or to the protocol or any other supporting documentation, that is likely to affect to a significant degree:

- the safety or physical or mental integrity of the subjects of the trial;
- the scientific value of the trial;
- the conduct or management of the trial; or
- the quality or safety of any intervention used in the trial.

## Annual progress report

The sponsor/investigator will submit a summary of the progress of the trial to the accredited METC once a year. Information will be provided on the date of inclusion of the first subject, numbers of subjects included and numbers of subjects that have completed the trial, serious adverse events/ serious adverse reactions, other problems, and amendments.

## Temporary halt and (prematurely) end of study report

The investigator/sponsor will notify the accredited METC of the end of the study within a period of 8 weeks. The end of the study is defined as the last patient’s last visit.

The sponsor will notify the METC immediately of a temporary halt of the study, including the reason of such an action.

In case the study is ended prematurely, the sponsor will notify the accredited METC within 15 days, including the reasons for the premature termination.

Within one year after the end of the study, the investigator/sponsor will submit a final study report with the results of the study, including any publications/abstracts of the study, to the accredited METC.

The sponsor will notify the accredited METC and the competent authority of the end of the study within a period of 90 days. The end of the study is defined as the last patient’s last visit.

## Public disclosure and publication policy

Patients are entitled to public disclosure of the results of the trial on the basis of their participation in it. The results of research will be submitted for publication to peer-reviewed scientific journals. Agreements with respect to participation in publication were made before the start of the trial. Authorship is granted to all people of the project group. Besides the project group and research fellows, authorship is granted to the local investigator of each center when at least ten patients are included in the trial and when substantial contribution to the trial (e.g. full completion of CRF or intellectual input) is made. Every other people who made substantial contribution to the trial will be added to the collaborator list.

# REFERENCES

1. Hain E, Maggiori L, Manceau G, Mongin C, Prost AlDJ, Panis Y. Oncological impact of anastomotic leakage after laparoscopic mesorectal excision. The British journal of surgery. 2017;104(3):288-95.
2. Borstlap WAA, Westerduin E, Aukema TS, Bemelman WA, Tanis PJ. Anastomotic Leakage and Chronic Presacral Sinus Formation After Low Anterior Resection: Results From a Large Cross-sectional Study. Annals of surgery. 2017;266(5):870-7.
3. Penna M, Hompes R, Arnold S, Wynn G, Austin R, Warusavitarne J, et al. Incidence and Risk Factors for Anastomotic Failure in 1594 Patients Treated by Transanal Total Mesorectal Excision: Results From the International TaTME Registry. Annals of surgery. 2018.
4. Kulu Y, Tarantio I, Warschkow R, Kny S, Schneider M, Schmied BM, et al. Anastomotic leakage is associated with impaired overall and disease-free survival after curative rectal cancer resection: a propensity score analysis. Annals of surgical oncology. 2015;22(6):2059-67.
5. Alverdy JC, Hyoju SK, Weigerinck M, Gilbert JA. The gut microbiome and the mechanism of surgical infection. The British journal of surgery. 2017;104(2):e14-e23.
6. Gaines S, Shao C, Hyman N, Alverdy JC. Gut microbiome influences on anastomotic leak and recurrence rates following colorectal cancer surgery. The British journal of surgery. 2018;105(2):e131-e41.
7. Shogan BD, Belogortseva N, Luong PM, Zaborin A, Lax S, Bethel C, et al. Collagen degradation and MMP9 activation by Enterococcus faecalis contribute to intestinal anastomotic leak. Science translational medicine. 2015;7(286):286ra68.
8. Flanagan L, Schmid J, Ebert M, Soucek P, Kunicka T, Liska V, et al. Fusobacterium nucleatum associates with stages of colorectal neoplasia development, colorectal cancer and disease outcome. European journal of clinical microbiology & infectious diseases : official publication of the European Society of Clinical Microbiology. 2014;33(8):1381-90.
9. Chen M, Song X, Chen LZ, Lin ZD, Zhang XL. Comparing Mechanical Bowel Preparation With Both Oral and Systemic Antibiotics Versus Mechanical Bowel Preparation and Systemic Antibiotics Alone for the Prevention of Surgical Site Infection After Elective Colorectal Surgery: A Meta-Analysis of Randomized Controlled Clinical Trials. Diseases of the colon and rectum. 2016;59(1):70-8.
10. Blanco-Colino R, Espin-Basany E. Intraoperative use of ICG fluorescence imaging to reduce the risk of anastomotic leakage in colorectal surgery: a systematic review and meta-analysis. Techniques in coloproctology. 2018;22(1):15-23.
11. Borstlap WAA, Musters GD, Stassen LPS, van Westreenen HL, Hess D, van Dieren S, et al. Vacuum-assisted early transanal closure of leaking low colorectal anastomoses: the CLEAN study. Surgical endoscopy. 2018;32(1):315-27.
12. McSorley ST, Steele CW, McMahon AJ. Meta-analysis of oral antibiotics, in combination with preoperative intravenous antibiotics and mechanical bowel preparation the day before surgery, compared with intravenous antibiotics and mechanical bowel preparation alone to reduce surgical-site infections in elective colorectal surgery. BJS open. 2018;2(4):185-94.
13. Vo E, Massarweh NN, Chai CY, Tran Cao HS, Zamani N, Abraham S, et al. Association of the Addition of Oral Antibiotics to Mechanical Bowel Preparation for Left Colon and Rectal Cancer Resections With Reduction of Surgical Site Infections. JAMA surgery. 2018;153(2):114-21.
14. Uchino M, Ikeuchi H, Bando T, Chohno T, Sasaki H, Horio Y, et al. Efficacy of Preoperative Oral Antibiotic Prophylaxis for the Prevention of Surgical Site Infections in Patients with Crohn Disease: A Randomized Controlled Trial. Annals of surgery. 2017.
15. Midura EF, Jung AD, Hanseman DJ, Dhar V, Shah SA, Rafferty JF, et al. Combination oral and mechanical bowel preparations decreases complications in both right and left colectomy. Surgery. 2018;163(3):528-34.
16. Leenen JPL, Hentzen J, Ockhuijsen HDL. Effectiveness of mechanical bowel preparation versus no preparation on anastomotic leakage in colorectal surgery: a systematic review and meta-analysis. Updates in surgery. 2018.
17. Koller SE, Bauer KW, Egleston BL, Smith R, Philp MM, Ross HM, et al. Comparative Effectiveness and Risks of Bowel Preparation Before Elective Colorectal Surgery. Annals of surgery. 2018;267(4):734-42.
18. Devane LA, Proud D, O'Connell PR, Panis Y. A European survey of bowel preparation in colorectal surgery. Colorectal disease : the official journal of the Association of Coloproctology of Great Britain and Ireland. 2017;19(11):O402-o6.
19. Buunen M, Lange MM, Ditzel M, Kleinrensink GJ, van de Velde CJ, Lange JF. Level of arterial ligation in total mesorectal excision (TME): an anatomical study. International journal of colorectal disease. 2009;24(11):1317-20.
20. Reddy SH, Gupta V, Yadav TD, Singh G, Sahni D. Lengthening of left colon after rectal resection: What all is adequate? A prospective cohort study. International journal of surgery (London, England). 2016;31:27-32.
21. Kye BH, Kim HJ, Kim HS, Kim JG, Cho HM. How much colonic redundancy could be obtained by splenic flexure mobilization in laparoscopic anterior or low anterior resection? International journal of medical sciences. 2014;11(9):857-62.
22. Araujo SE, Seid VE, Kim NJ, Bertoncini AB, Nahas SC, Cecconello I. Assessing the extent of colon lengthening due to splenic flexure mobilization techniques: a cadaver study. Arquivos de gastroenterologia. 2012;49(3):219-22.
23. Mangano A, Gheza F, Giulianotti PC. Iatrogenic spleen injury during minimally invasive left colonic flexure mobilization: the quest for evidence-based results. Minerva chirurgica. 2018;73(5):512-9.
24. Mouw TJ, King C, Ashcraft JH, Valentino JD, DiPasco PJ, Al-Kasspooles M. Routine splenic flexure mobilization may increase compliance with pathological quality metrics in patients undergoing low anterior resection. Colorectal disease : the official journal of the Association of Coloproctology of Great Britain and Ireland. 2018.
25. Jafari MD, Wexner SD, Martz JE, McLemore EC, Margolin DA, Sherwinter DA, et al. Perfusion assessment in laparoscopic left-sided/anterior resection (PILLAR II): a multi-institutional study. Journal of the American College of Surgeons. 2015;220(1):82-92.e1.
26. Schaafsma BE, Mieog JS, Hutteman M, van der Vorst JR, Kuppen PJ, Lowik CW, et al. The clinical use of indocyanine green as a near-infrared fluorescent contrast agent for image-guided oncologic surgery. Journal of surgical oncology. 2011;104(3):323-32.
27. Ris F, Liot E, Buchs NC, Kraus R, Ismael G, Belfontali V, et al. Multicentre phase II trial of near-infrared imaging in elective colorectal surgery. The British journal of surgery. 2018.
28. van den Bos J, Al-Taher M, Schols RM, van Kuijk S, Bouvy ND, Stassen LPS. Near-Infrared Fluorescence Imaging for Real-Time Intraoperative Guidance in Anastomotic Colorectal Surgery: A Systematic Review of Literature. Journal of laparoendoscopic & advanced surgical techniques Part A. 2018;28(2):157-67.
29. Singh PP, Zeng IS, Srinivasa S, Lemanu DP, Connolly AB, Hill AG. Systematic review and meta-analysis of use of serum C-reactive protein levels to predict anastomotic leak after colorectal surgery. The British journal of surgery. 2014;101(4):339-46.
30. Adamina M, Steffen T, Tarantino I, Beutner U, Schmied BM, Warschkow R. Meta-analysis of the predictive value of C-reactive protein for infectious complications in abdominal surgery. The British journal of surgery. 2015;102(6):590-8.
31. Warschkow R, Beutner U, Steffen T, Muller SA, Schmied BM, Guller U, et al. Safe and early discharge after colorectal surgery due to C-reactive protein: a diagnostic meta-analysis of 1832 patients. Annals of surgery. 2012;256(2):245-50.
32. van Koperen PJ, van der Zaag ES, Omloo JM, Slors JF, Bemelman WA. The persisting presacral sinus after anastomotic leakage following anterior resection or restorative proctocolectomy. Colorectal disease : the official journal of the Association of Coloproctology of Great Britain and Ireland. 2011;13(1):26-9.
33. Musters GD, Borstlap WA, Bemelman WA, Buskens CJ, Tanis PJ. Intersphincteric completion proctectomy with omentoplasty for chronic presacral sinus after low anterior resection for rectal cancer. Colorectal disease : the official journal of the Association of Coloproctology of Great Britain and Ireland. 2016;18(2):147-54.
34. van Koperen PJ, van Berge Henegouwen MI, Rosman C, Bakker CM, Heres P, Slors JF, et al. The Dutch multicenter experience of the endo-sponge treatment for anastomotic leakage after colorectal surgery. Surgical endoscopy. 2009;23(6):1379-83.
35. Gardenbroek TJ, Musters GD, Buskens CJ, Ponsioen CY, D'Haens GR, Dijkgraaf MG, et al. Early reconstruction of the leaking ileal pouch-anal anastomosis: a novel solution to an old problem. Colorectal disease : the official journal of the Association of Coloproctology of Great Britain and Ireland. 2015;17(5):426-32.
36. Ris F, Hompes R, Lindsey I, Cunningham C, Mortensen NJ, Cahill RA. Near infra-red laparoscopic assessment of the adequacy of blood perfusion of intestinal anastomosis - a video vignette. Colorectal disease : the official journal of the Association of Coloproctology of Great Britain and Ireland. 2014;16(8):646-7.
37. Ris F, Hompes R, Cunningham C, Lindsey I, Guy R, Jones O, et al. Near-infrared (NIR) perfusion angiography in minimally invasive colorectal surgery. Surgical endoscopy. 2014;28(7):2221-6.
38. James DR, Ris F, Yeung TM, Kraus R, Buchs NC, Mortensen NJ, et al. Fluorescence angiography in laparoscopic low rectal and anorectal anastomoses with pinpoint perfusion imaging--a critical appraisal with specific focus on leak risk reduction. Colorectal disease : the official journal of the Association of Coloproctology of Great Britain and Ireland. 2015;17 Suppl 3:16-21.
39. Allegranzi B, Bischoff P, de Jonge S, Kubilay NZ, Zayed B, Gomes SM, et al. New WHO recommendations on preoperative measures for surgical site infection prevention: an evidence-based global perspective. The Lancet Infectious diseases. 2016;16(12):e276-e87.
40. Mulder T, Crolla R, Kluytmans-van den Bergh MFQ, van Mourik MSM, Romme J, van der Schelling GP, et al. Preoperative oral antibiotic prophylaxis reduces surgical site infections after elective colorectal surgery: results from a before-after study. Clinical infectious diseases : an official publication of the Infectious Diseases Society of America. 2018.
41. Roos D, Dijksman LM, Tijssen JG, Gouma DJ, Gerhards MF, Oudemans-van Straaten HM. Systematic review of perioperative selective decontamination of the digestive tract in elective gastrointestinal surgery. The British journal of surgery. 2013;100(12):1579-88.
42. Abis GS, Oosterling SJ, Stockmann HB, van der Bij GJ, van Egmond M, Vandenbroucke-Grauls CM, et al. Perioperative selective decontamination of the digestive tract and standard antibiotic prophylaxis versus standard antibiotic prophylaxis alone in elective colorectal cancer patients. Danish medical journal. 2014;61(4):A4695.
43. Mulder T, Kluytmans-van den Bergh MFQ, de Smet A, van 't Veer NE, Roos D, Nikolakopoulos S, et al. Prevention of severe infectious complications after colorectal surgery using preoperative orally administered antibiotic prophylaxis (PreCaution): study protocol for a randomized controlled trial. Trials. 2018;19(1):51.
44. Moran BJ, Holm T, Brannagan G, Chave H, Quirke P, West N, et al. The English national low rectal cancer development programme: key messages and future perspectives. Colorectal disease : the official journal of the Association of Coloproctology of Great Britain and Ireland. 2014;16(3):173-8.
45. Roodbeen SX, Penna M, Mackenzie H, Kusters M, Slater A, Jones OM, et al. Transanal total mesorectal excision (TaTME) versus laparoscopic TME for MRI-defined low rectal cancer: a propensity score-matched analysis of oncological outcomes. Surgical endoscopy. 2018.
46. Bastiaenen VP, Hovdenak Jakobsen I, Labianca R, Martling A, Morton DG, Primrose JN, et al. Consensus and controversies regarding follow-up after treatment with curative intent of nonmetastatic colorectal cancer: a synopsis of guidelines used in countries represented in the European Society of Coloproctology. Colorectal disease : the official journal of the Association of Coloproctology of Great Britain and Ireland. 2018.

**Appendix:**

**A: Organogram B: Trial design**

**C: Post-operative algorithm**

**D: Pro-active management algorithm**

**E: Schema te doorlopen stappen voor deelnemers F: Risk assessment**

**NL67600.018.18 IMARI-trial**

## Appendix A: Organogram


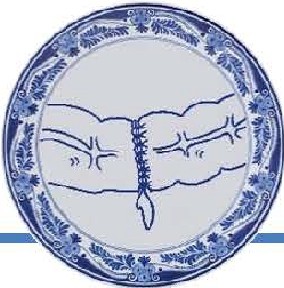


**Organogram**

**IMARI**

Amsterdam UMC, location AMC


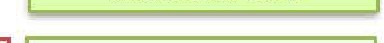


**Dr. J.H. Wijsman** Surgeon Amphia

**Dr. E.C.J. Consten**

Surgeon Meander MC

| **Drs. e.v. Nio**  Radiologist  - |  | **Prof. dr. C.I.J. Pensioen**  Gastro-enterologist |  | **Prof. dr. W.A. Bemelman**  Surgeon | **Prof. dr. M.A. Boermeester** Surgeon |
| --- | --- | --- | --- | --- | --- |
|  |  |  |  |  |  |
| **Drs. M.D. Slooter**  PhD candidate |  | **Dr. G.O. Musters**  Surgeon in training |  | **Dr. P.J. Tanis**  Surgeon | **Drs. R. Hompes**  Surgeon |

Coordinating researcher

**Dr. S. van Dieren**

Statisticus

Local data manager

Amphia Hospita!, Breda Jeroen Bosch Hospita 1, Den Bosch Amsterdam UMC, location AMC, Amsterdam Maastricht UMC, Maastricht Amsterdam UMC, location VUMC, Amsterdam Meander Medical Center, Amersfoort Bernhoven Hospita!, Uden, Oss Medical Spectrum Twente, Enschede

Catharina Hospita!, Eindhoven OLVG, Amsterdam

Flevo Hospita!, Almere Radboud UMC, Nijmegen

Hospita! Gelderse Vallei, Ede Spaarne Gasthuis, Haarlem, Hoofddorp Hospita! Group Twente, Almelo, Hengelo IJsselland Hospita!, Capelle aan **de** IJssel Isa la, Zwolle, Meppel, Kampen, Steenwijk, Heerde

**Project**


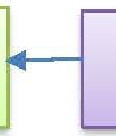


**group**

---� ,�-

**Project Dl**

**leader** &

**PI**

**Coordinating**

i**nvestigato r**

�----�

**Participating centers**

-------

**NL67600.018.18 IMARI-trial**

## Appendix B: Trial Design

**
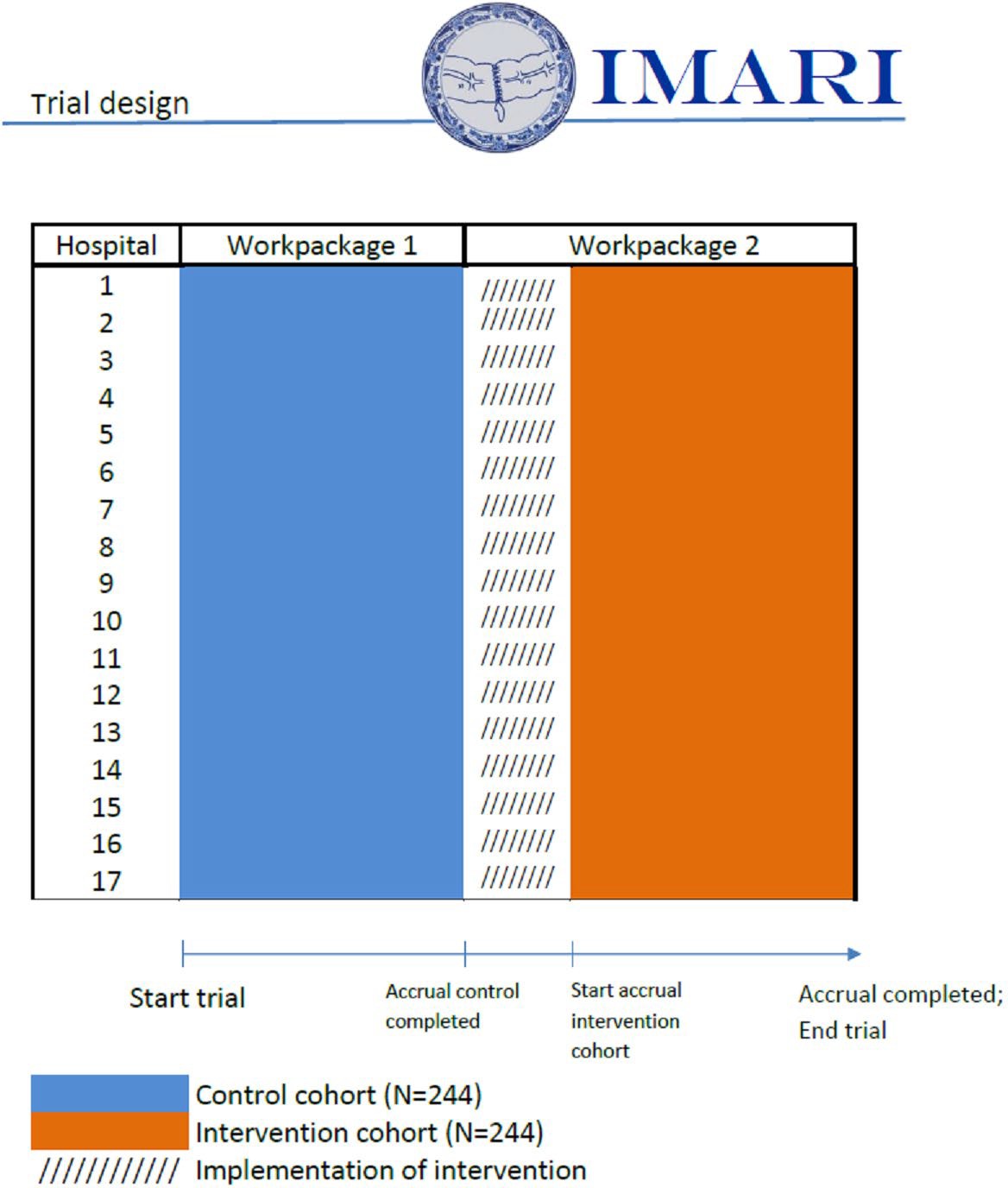
**

## Appendix C: Post-operative algorithm


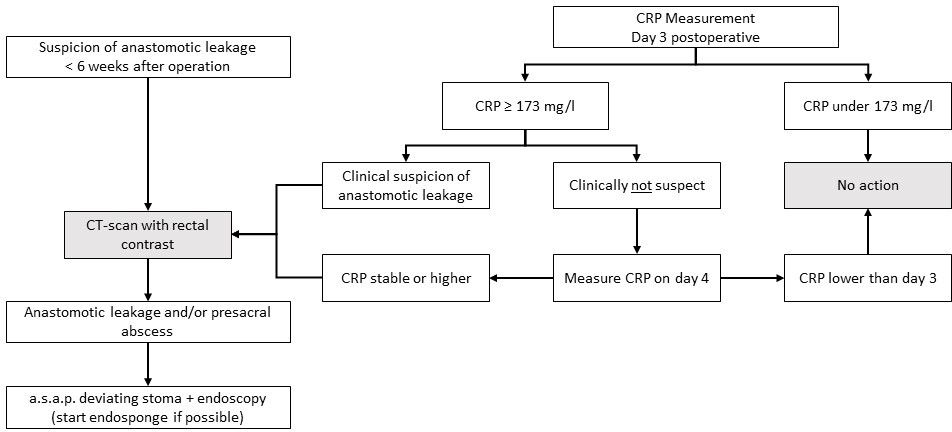


## Appendix D: Pro-active management algorithm

**
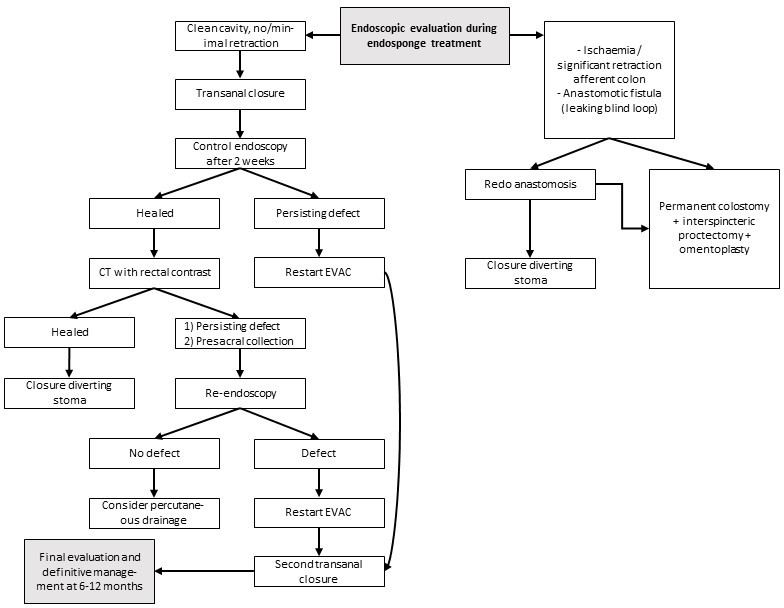
**

## Appendix E: Schema te doorlopen stappen voor deelnemers


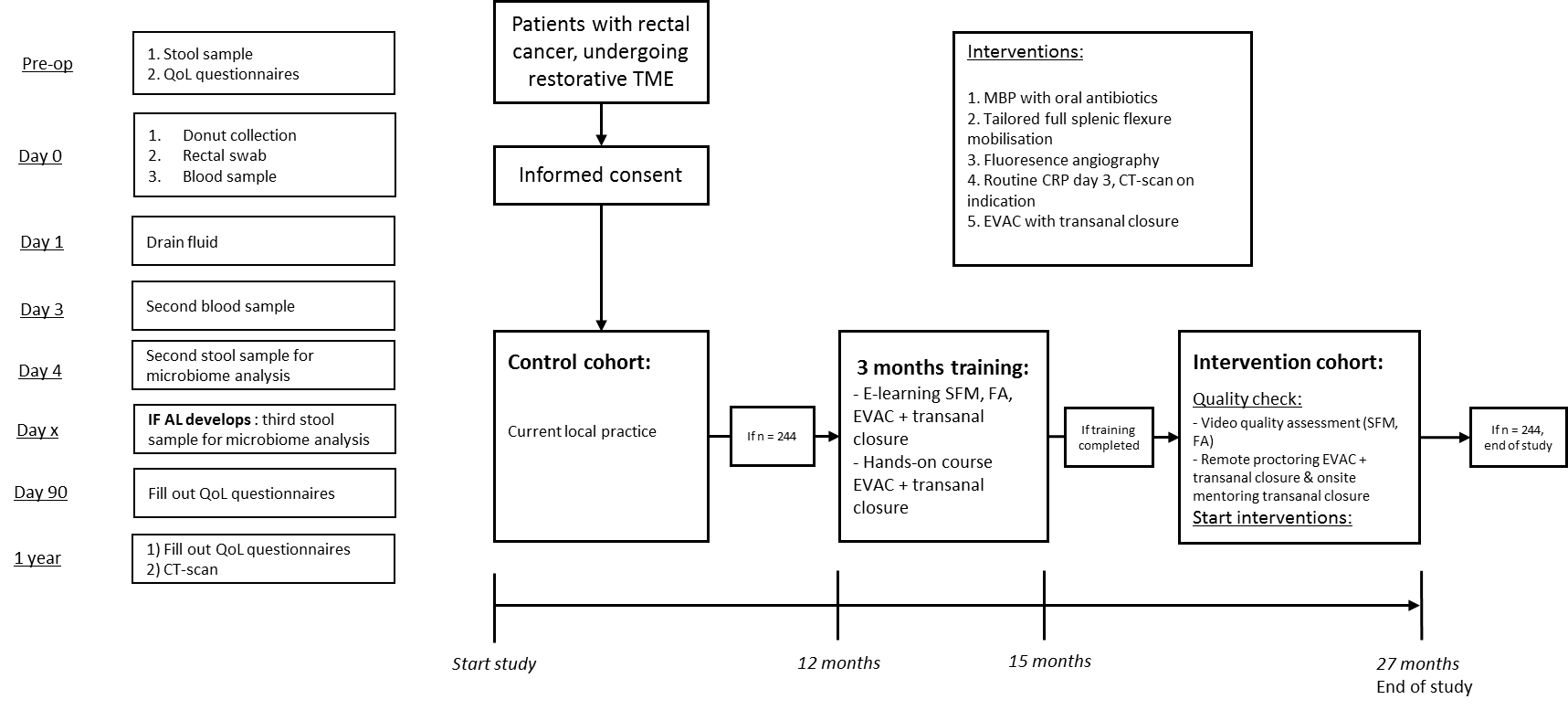


**NL67600.018.18 IMARI-trial**

## F: Risk assessment

Clinical; Research Unit


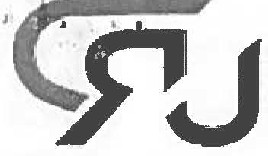


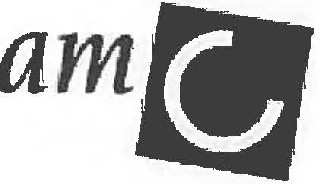


**Risk assessment in clinical research projects regarding the required management and monitoring strategy**

*Thisform can befi/led in electronical/y. The available text fields can be extended as needed.*

| **Full title of the study:** Multi-lnterventional program for prevention and early Management of Anastomotic leakage after total mesorectal excision in Rectal cancer patlents, the IMARl-trial |
| --- |
| **Principal lnvestigator** *(In Dutch: Hoofdonderzoeker)*  Name: drs. R. Hompes Department: Surgery |
| **Risk analysis conducted by:** *(til/ in all project team members that contributed)*  Name, department, project role: drs. M.D. Slooter, surgery, coordinating investigator  Name, department, project role: drs. S. Sharabiany, surgery, coordination investigator  Name, department, project role: dr. P.J. Tanis, surgery, project leader and principal investigator  Name, department, project role: |
| **Date of completlon:** *<dd-mmm-yyyy>* 5th of March 2019 |

*This tool is based on other risk assessment approaches^1^-3 and adapted for Academie Medica/*

*Center (AMC) investigator-initated research projects by the AMC-Clinica/ Research Unit (AMC-CRU).*

1 ***This tool Is developed by the AMC-CRU and is the confidential information of the AMC-CRU. lt Is*** 1

**NL67600.018.18 IMARI-trial**


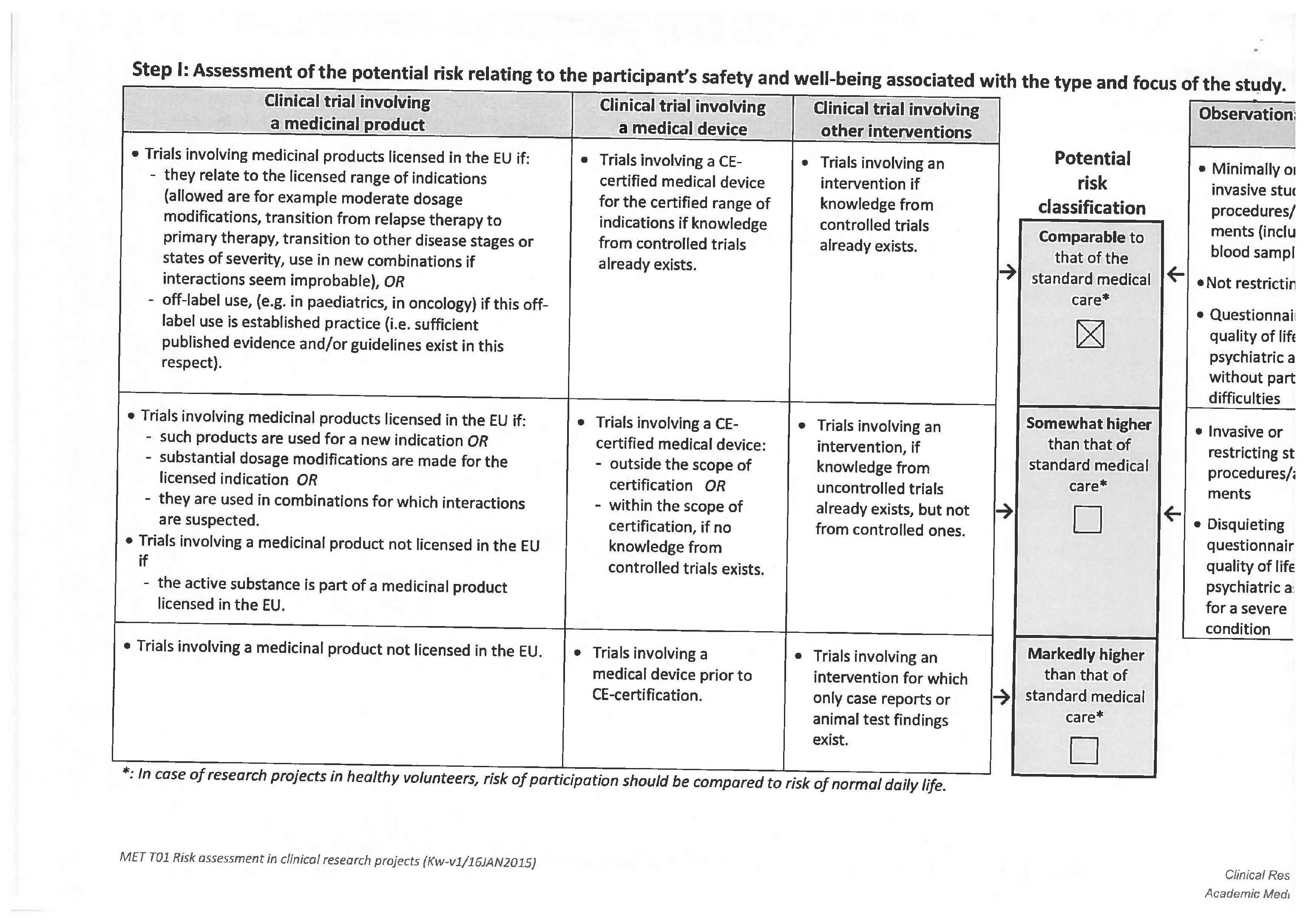


**Step** Il: **ldentification of further study specific factors.**

*Review the research protocol to identify specificfactors that are critica/ for the participants' safety and we/1-being and/or rlghts and/or the va/idity of the resu/ts.*

*Here again, for participant-related risks, the risks identified should be balanced against the risks a participant would run if treated outside a study protocol. For each risk identified, consider the appropriate study management and/or monitoring strategy.*

*For guidance for each specific factor see Addendum.*

| **Fl. Potentially vulnerable populatlon?** |
| --- |
| **a. Will a vulnerable population be lncluded?**  D YES: *Continue with b;* C8;l NO: *Continue with F2.* |
| **b. Does it mean a higher risk?** *(check all that apply)*  D YES, for participant's safety, well-being D YES, for participant's rights D YES, for data validity  C8;] NO  *lf 'YES' was chosen at least once, continue with c and d;*  *IJ 'NO' was chosen, continue with F2.* |
| **c. Which study management measures will be taken to control thls risk?** |
| **d. Could on-site monitoring independently contribute to risk management in conjunction with the above mentioned control measures?**  0 YES 0 NO |

| **F2. Emergency medica! treatment?** |
| --- |
| **a. Will trial participants be recruited within the scope of emergency medical treatment?**  D YES: *Continue with b;* C8;l NO: *Continue with F3.* |
| **b. Does it mean a higher risk?** *(check all that apply)*  D YES, for partlcipant's safety, well-being D YES, for participant's rights D YES, for data validity  C8;] NO  *Jf 'YES' was chosen at least once, continue with c and d; lf 'NO' was chosen, continue with F3.* |
| **c. Which study management measures will be taken to control thls risk?** |
| **d. Could on-site monitoring independently contribute to risk management in conjunction with the above mentioned control measures?**  0 YES 0 NO |

| **F3. Eligibility criteria** |
| --- |
| **a. Are there any critica! ellglbility criteria?**  D YES: *Continue with b;* 12] NO: *Continue with F4.* |
| **b. Does lt mean a higher risk?** *(check all that apply)*  D YES, for participant's safety, well-being D YES, for participant's rights D YES, for data validity 12] **NO**  *IJ'YES' was chosen at least once, continue with c and d; lf 'NO' was chosen, continue with F4.* |
| **c. Which study management measures will be taken to control this risk?** |
| **d. Could on-site monitoring independently contribute to risk management in conjunction with the above mentioned control measures?**  0 YES 0 NO |

| **F4. Addltional prescription medication for concomitant dlseases/symptoms** |
| --- |
| **a. Is it likely that participants receive additional medication for concomitant diseases/symptoms?**  D YES: *Continue with b;* 12] NO: *Continue with FS.* |
| **b. Does it mean a higher risk?** *(check all that apply)*  D YES, for participant's safety, well-being D YES, for participant's rights D YES, for data validity 12] **NO**  *IJ 'YES' was chosen at least once, continue with c and d; lf 'NO' was chosen, continue with FS.* |
| **c. Which study management measures will be taken to control thls risk?** |
| **d. Could on-site monitoring independently contribute to risk management** in **conjunction with the**  above mentioned control measures?  0 YES 0 NO |

| **FS. Lack or limited knowledge about the (combination of) intervention(s)** |
| --- |
| **a. Is there a lack of or only very limited knowledge about the (combination) of intervention(s) being investigated?**  � YES: *Continue with b;* 0 NO: *Continue with F6.* |
| **b. Does it mean a higher risk?** *(check all that apply)*  0 YES, for participant's safety, well-being O YES, for participant's rights O YES, for data validity  �NO  *lf 'YES' was chosen at least once, continue with c and d; lf 'NO' was chosen, continue with F6.* |
| **c. Which study management measures will be taken to control this risk?** |
| **d. Could on-site monitoring independently contribute to risk management** in **conjunction with the above mentioned control measures?**  0 YES 0 NO |

| **F6. Risks due to other study related procedures** |
| --- |
| **a. Are any additional study procedures performed that carry significant risk, i.e. other than the interventlon(s) belng tested, and that are not part of standard care?**  � YES: *Continue with b;* 0 NO: *Continue with F7.* |
| **b. Does it mean a higher risk?** *(check all that apply)*  0 YES, for participant's safety, well-being O YES, for participant's rights O YES, *tor* data validity  �NO  *lf 'YES' was chosen at least once, continue with c and d; lf 'NO' was chosen, continue with Fl.* |
| **c. Which study management measures will be taken to control this risk?** |
| **d. Could on-site monitoring independently contrlbute to risk management in conjunction with the above mentioned control measures?**  0 YES 0 NO |

.. 9

| **F7. Risks due to harriers to compliance with the study protocol** |
| --- |
| **a. Is the study complex and/or unusual compared to standard medica! care, so compliance to the study protocol may be difficult for the site and/or participant? And/or any other harriers for compliance?**  IZI YES: *Continue with b;* 0 NO: *Continue with FB.* |
| **b. Does it mean a higher risk?** *(check all that apply)*  0 YES, for participant's safety, well-being O YES, for participant's rights O YES, for data validity  IZI NO  *lf 'YES' was chosen at least once, continue with c and d; lf 'NO' was chosen, continue with FB.* |
| **c. Which study management measures will be taken to control this risk?** |
| **d. Could on-site monitoring independently contribute to risk management** in **conjunction with the above mentioned control measures?**  0 YES 0 NO |

| FS. Risks due to participating sites |
| --- |
| **a. Are sites** included **that introduce particular vulnerabilitles, e.g. inexperienced/under• resourced sites/research teams?**  0 YES: *Continue with b;* IZI NO: *Continue with F9.* |
| **b. Does it mean a higher risk?** *(check all that apply)*  0 YES, for participant's safety, well-being O YES, for participant's rights D YES, for data validity  IZI NO  *lf 'YES' was chosen at least once, continue with c and d;*  */f 'NO' was chosen, continue with F9.* |
| **c. Which study management measures will be taken to controi this risk?** |
| **d. Could on-site monitoring independently contrlbute to risk management in conjunction with the above mentioned control measures?**  0 YES 0 NO |

| **F9. Risks from data collection and handling methods** |
| --- |
| **a. Are data collection and handling methods complex/under-resourced and/or are any particularly sensitive data being collected?**  0 YES: *Continue with b;* � NO: *Continue with F10.* |
| **b. Does it mean a higher risk?** *(check all that apply)*  0 YES, for participant's safety, well-being O YES, for participant's rights O YES, **for** data validity  �NO  *lf 'YES' was chosen at least once, continue with c and d;*  */f 'NO' was chosen, continue with F10.* |
| **c. Which study management measures will be taken to control this risk?** |
| **d. Could on-site monitoring independently contribute to risk management** in **conjunction with the other above mentioned measures?**  0 YES 0 NO |

| **FlO. Risks to the AMC organisation** |
| --- |
| **a/b. Will the study concern aspects that carry a risk to the AMC organisation for the reputation and/or liability and/or financials?** *(check all that apply)*  0 YES, for the reputation 0 YES, for liability 0 YES, for financia\s  � **NO**  *Jf 'YES' was chosen at least once, continue with c and d; lf 'NO' was chosen, continue with F11* . |
| **c. Which study management measures will be taken to control this risk?** |
| **d. Could on-site monitoring independently contribute to risk management in conjunction with the above mentioned control measures?**  0 YES 0 NO |


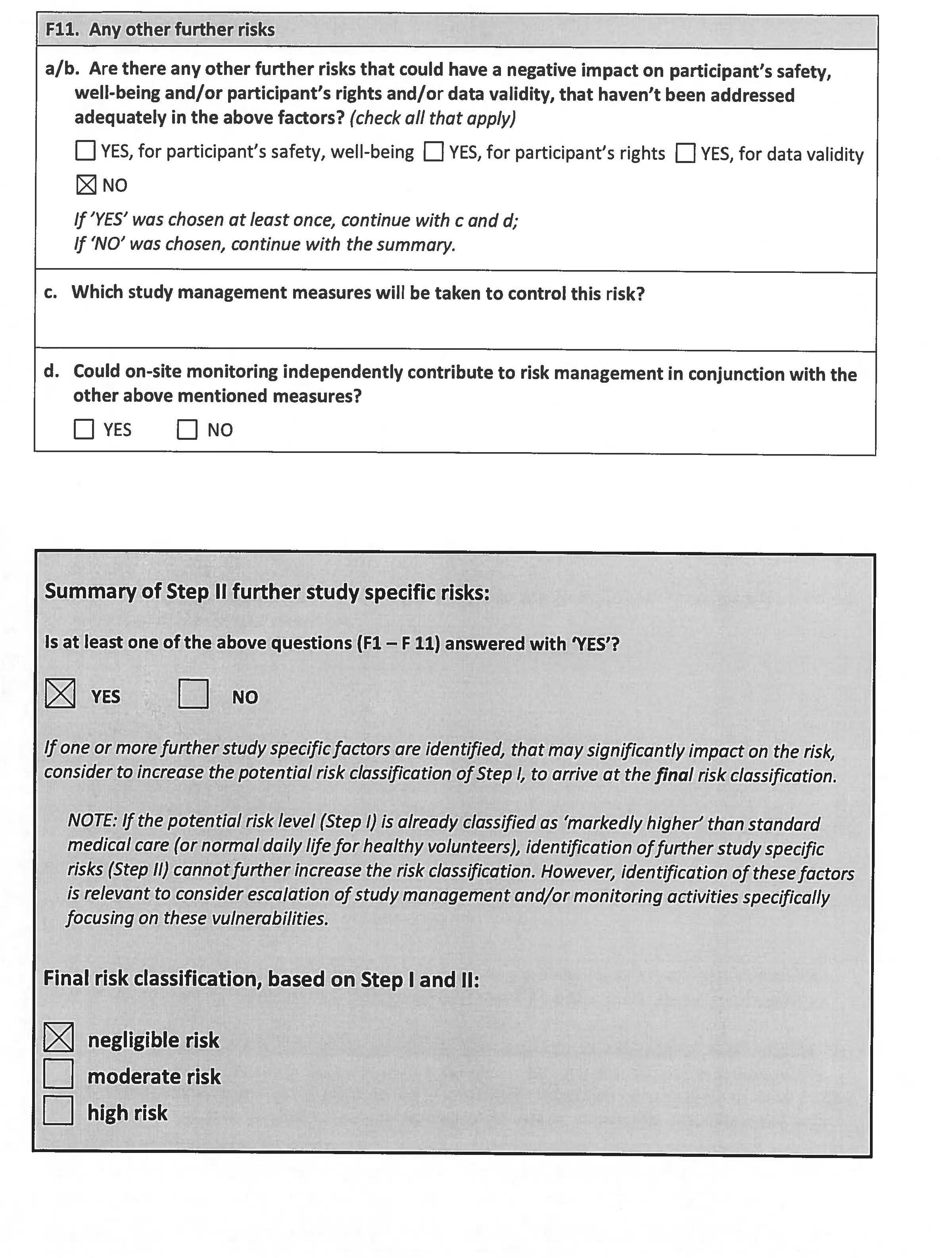

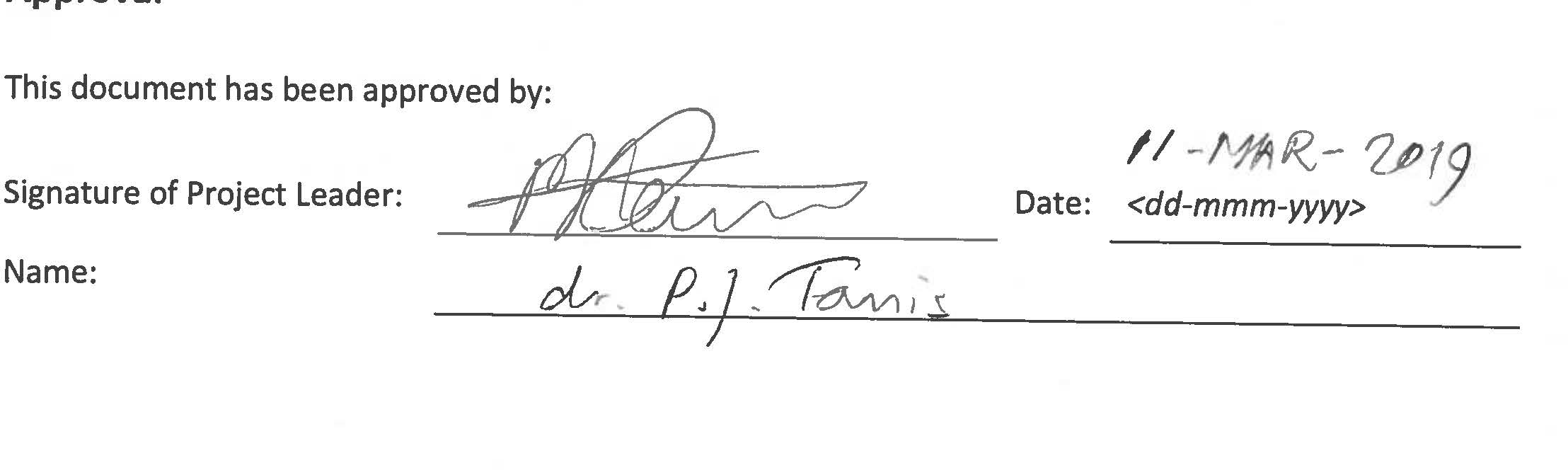

Supplement: zrag046_Supplementary_Data [file zrag046_supplementary_data.docx]
